# Synthesis, Radiolabelling and In Vitro Imaging of Multifunctional Nanoceramics

**DOI:** 10.1002/cnma.201700378

**Published:** 2018-02-08

**Authors:** Marina Lledos, Vincenzo Mirabello, Sophia Sarpaki, Haobo Ge, Hubert J. Smugowski, Laurence Carroll, Eric O. Aboagye, Franklin I. Aigbirhio, Stanley W. Botchway, Jonathan R. Dilworth, David G. Calatayud, Pawel K. Plucinski, Gareth J. Price, Sofia I. Pascu

**Affiliations:** ^1^ Department of Chemistry University of Bath, Claverton Down BA2 7AY Bath UK; ^2^ Department of Surgery and Cancer, Faculty of Medicine, Commonwealth Building, Hammersmith Campus Imperial College London Du Cane Road London W12 0NN UK; ^3^ Wolfson Brain Imaging Centre, Department of Clinical Neurosciences University of Cambridge Cambridge UK; ^4^ Central Laser Facility, Rutherford Appleton Laboratory Research Complex at Harwell STFC Didcot OX11 0QX UK; ^5^ Department of Chemistry University of Oxford South Parks Road Oxford OX1 3PA UK; ^6^ Department of Electroceramics Instituto de Ceramica y Vidrio – CSIC Kelsen 5, Campus de Cantoblanco 28049 Madrid Spain; ^7^ Department of Chemical Engineering University of Bath, Claverton Down BA2 7AY Bath UK

**Keywords:** core-shell nanoparticles, cellular bioimaging, hypoxia, radiochemistry, self-assembly

## Abstract

Molecular imaging has become a powerful technique in preclinical and clinical research aiming towards the diagnosis of many diseases. In this work, we address the synthetic challenges in achieving lab‐scale, batch‐to‐batch reproducible copper‐64‐ and gallium‐68‐radiolabelled metal nanoparticles (MNPs) for cellular imaging purposes. Composite NPs incorporating magnetic iron oxide cores with luminescent quantum dots were simultaneously encapsulated within a thin silica shell, yielding water‐dispersible, biocompatible and luminescent NPs. Scalable surface modification protocols to attach the radioisotopes ^64^Cu (t_1/2_=12.7 h) and ^68^Ga (t_1/2_=68 min) in high yields are reported, and are compatible with the time frame of radiolabelling. Confocal and fluorescence lifetime imaging studies confirm the uptake of the encapsulated imaging agents and their cytoplasmic localisation in prostate cancer (PC‐3) cells. Cellular viability assays show that the biocompatibility of the system is improved when the fluorophores are encapsulated within a silica shell. The functional and biocompatible SiO_2_ matrix represents an ideal platform for the incorporation of ^64^Cu and ^68^Ga radioisotopes with high radiolabelling incorporation.

## Introduction

The self‐assembly and subsequent surface functionalization of mesoporous silica nanoparticles enable the formation of new materials with highly controllable properties for theranostic nanomedicine.^[1],[2]^ Molecular imaging plays a key role in personalized and targeted medicine.^3^ Each type of *in‐vivo* imaging technique has its own advantages and limitations such as spatial and/or temporal resolution, sensitivity, signal‐to‐noise ratio SNR, penetration depth in tissue, quantitative accuracy^4^ and differentiating postsurgical residual disease and postchemotherapy/postradiation lesions.^5^ It has been acknowledged that there is no single modality, available amongst current molecular imaging techniques, capable to acquire alone all of the essential information across length scales of molecules to tissues and organs. Positron emission tomography (PET) is very sensitive and highly quantitative but has a poor spatial resolution. Magnetic resonance imaging (MRI) has superior resolution compared to PET as well as good soft tissue contrast, but low sensitivity. Optical fluorescence imaging cannot accurately quantify *in‐vivo* fluorescence signals in large (more than 10 mm) living subjects. Thus, a combination of techniques (‘multimodal imaging’) is an essential tool in imaging at the research stage and in translational studies to a clinical setting.^6^,^7^,^8^,^9^ Simultaneous PET‐MR imaging is one example,^10^ which shows promise for the next generation of dual‐modality medical imaging.^11^ This requires reliable and batch‐to‐batch reproducible synthetic methods for new classes of imaging probes.^12^ Currently, the most studied dual‐modal imaging agent is based on a PET isotope combined with gadolinium for MRI,^13^ but longer‐term concerns include the deposition of Gd from linear chelators.^14^ Insoluble deposits of gadolinium phosphate have been found in many organs such as skin, liver, lungs, ileum, kidney, skeletal muscles and brain.14b,^15^

Recently developed dual‐mode contrast agents are based on benign magnetic nanoparticles (MNPs) and are starting to be used in clinical trials for MRI.^16^,^17^,^18^ Such MNPs have iron oxide cores that are superparamagnetic and so enable tracking of theranostic nanomedicines by MRI.^19^,^20^ Stoddart *et al*. created a targeted drug delivery system for the treatment of cancer and degenerative diseases.^21^,^22^ To date, examples of fully characterised dual‐(multi)‐mode imaging probes have been described for simultaneous PET/MRI or PET/MRI/NIRF (near infrared fluorescence).^23^ The preparation of serum albumin modified MnFe_2_O_4_ nanoparticles conjugated with ^124^I has been reported.^7^ Amino acid modified MNPs coupled to cyclic arginine‐glycine‐aspartic (RGD) peptides for integrin α_v_β_3_ targeting with macrocyclic 1,4,7,10‐tetraazacyclododecane‐N,N′,N′′,N′′′‐tetraacetic acid (DOTA) chelators labelled with ^64^Cu (t_1/2_=12.7 h) for PET were also described.^24^ A route to radiolabelling of dextran sulphate‐coated superparamagnetic iron oxide nanoparticles with ^64^Cu^25^ was developed where the radioactive centres were coordinated to the chelating bifunctional ligand, S‐2‐(4‐isothiocyanatobenzyl)‐1,4,7,10‐tetraazacyclododecane‐1,4,7,10‐tetraacetic acid (p‐SCN‐Bz‐DOTA), and subsequently conjugated to the surface of the MNPs. Devaraj *et al*. reported the synthesis and *in vivo* characterisation of ^18^F modified tri‐modal MNPs^26^ consisting of a shell of cross‐linked dextran, around a core of iron oxide and functionalized with the radionuclide ^18^F in high yields *via* click chemistry. Iron oxide MNPs with a compact human serum albumin coating (HSA‐IONPS), dually labelled with [^64^Cu]Cu‐DOTA and Cy 5.5, were synthesised^27^ as a tri‐modal imaging agent for PET/MRI/NIRF. The synthesis of a probe consisting of a superparamagnetic iron oxide (SPIO) core coated with PEGylated phospholipids has been reported.^11^ PEG ends were conjugated to DOTA to allow labelling with ^64^Cu. PEG chains were also used together with bisphosphonate anchors to SPIO NPs with [^99m^Tc] complexes, resulting in a multimodal in vivo imaging agent.^28^ Bisphosphonate ligands were also designed to bridge ^64^Cu and SPIO NPs.^29^ Green and Blower reported the synthesis, characterisation of a series of Co_x_Fe_(3‐x)_O_4_@NaYF_4_ core‐shell NPs doped with ^18^F, offering PET/SPECT, MR and up‐conversion fluorescence imaging.^30^ However, in most of these cases, complex chemical reactions are needed to radiolabel the MNPs or to anchor the fluorescent dyes. A potential problem is a possible solvolysis and/or MNP degradation that may occur over time in aqueous media and, to a higher extent, in a biological environment.^29^ In order to extend the use of the new functional MNPs to the radiopharmaceuticals, these synthetic challenges at their assembly need to be urgently addressed. Encapsulation of a molecular imaging probe for functional imaging and/or therapeutic applications within a magnetic nanoparticle represents an excellent method for kinetic stabilisation and successful delivery of the probe to the targeted tissue.^31^ Coating MNPs with silica is a promising approach since it is readily derivatised and it also reduces non‐target cytotoxicity.^11^,^32^,^33^,^34^,^35^,^36^,^37^ Furthermore, the use of quantum dots (QDs) in live‐cell imaging studies has been shown to be valuable due to both their capacity as luminescent agents capable of absorption across broad wavelengths but sharp emission bands and their superior resistance to photo‐bleaching compared with most commercial dyes.^38^,^39^,^40^,^41^ However, it has been reported that, although core‐shell QDs can be more robust than those with organic coatings, the thickness of the inorganic shell (ZnS) can degrade the optical properties of the core.^42^ Their usefulness as imaging agents has also been hampered by controversies related to the potential toxicity of the shell materials. There is a need for encapsulation techniques using benign materials with retention of efficient emission in biological media and provision of highly kinetically stable species.^43^,^44^ Silica is an attractive alternative in this regard since, as noted above, it is biocompatible and can be easily functionalized.^45^

**Figure 1:**
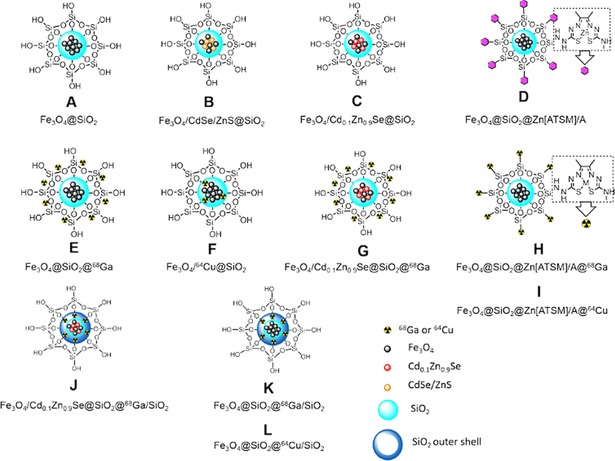
Schematic representation of the magnetic nanocomposites studied in this work.

This work aims to overcome the limitations of current systems to develop a novel series of MNPs for imaging purposes (Figure 1). Reproducible synthesis and characterisation protocols of such MNPs incorporating imaging agents such as QDs (Cd_0.1_Zn_0.9_Se and CdSe/ZnS) or Zn(II)[ATSM]/A, [^68^Ga]Ga[ATSM]/A (ATSM/A=diacetyl‐2‐(4‐N‐methyl‐3‐thiosemicarbazonato)‐3‐(4‐N‐amino‐3‐thiosemicarbazonato) and hypoxia tracers [^64^Cu]Cu(OAc)_2_ and [^64^Cu]Cu[ATSM]/A^46^ are reported.

The radioactive tracers [^64^Cu]Cu(OAc)_2_ and [^64^Cu]Cu[ATSM]/A display appreciable hypoxia selectivity versus normoxia, and can be used for the delineation of tumour cells deprived of oxygen,^47^ as well as ischemic and hypoxic myocardium.^48^ The metal complex was first used in clinical trials in 2000 as an imaging agent for tumor hypoxia.^49^ Ga[ATSM]/A is a new system that so far has not shown sufficient kinetic stability in the radiolabelled form.^50^,^51^,^52^ We and others^31^ have also designed and developed new methods to incorporate radioisotopes to chelator‐free nanoparticles, and characterised emerging nanomedicines *in vitro* and *in vivo*. New core‐shell nanomaterials with high kinetic stability in aqueous media are reported hereby.

New nanoceramics based on hybrid stystems denoted NPs and MNPs were generated *via* self‐assembled techniques, using a novel microemulsion method while encapsulating a range of imaging agents, including: nano‐materials‐based species (QDs), model inorganic systems for SPECT tracers such as 99mTc‐pertechnetate and/or radiotherapy (Re(VII) oxo‐anionic species, ReO_4_^‐^_,_ see ESI), radioactive ions for PET (^64^Cu(II) and ^68^Ga(III)) and corresponding radiotracer metal complexes and precursors ([^64^Cu]Cu(ATSM) and also their Ga(III) analogues). Fluorophores were encapsulated within a silica shell to investigate the *in vitro* uptake in prostate cancer cells (PC‐3), a non‐cancerous fibroblast cell line (FEK‐4) and Chinese hamster ovary (CHO). Multiphoton fluorescence spectroscopy and imaging techniques were used for the first time to describe the environmental changes in the nature of the fluorophore upon encapsulation in NPs/MNPs and upon internalisation in cells.The potential for simultaneous and versatile derivatisation of NPs and MNPs whilst retaining their kinetic stability in the aqueous and biological environment are probed extensively by a range of spectroscopic and imaging techniques and discussed hereby for the first time.

## Results and Discussion

### Incorporation of Optical Imaging Agents into Core‐shell Fe_3_O_4_@SiO_2_ Magnetic Nanoparticles (MNPs)

**Figure 2:**
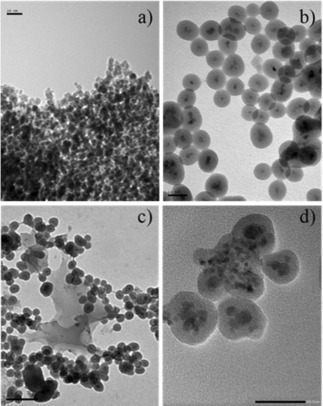
TEM micrographs of a) Fe_3_O_4_, b) Fe_3_O_4_@SiO_2_ (**A**), c) Fe_3_O_4_@SiO_2_@Zn[ATSM]/A (**D**), and d) Fe_3_O_4_/Cd_0.1_Zn_0.9_Se@SiO_2_ (**C**).

Optimising the efficacy of a (dark) contrast MRI agent has been one of the challenges that attracted the attention of many research groups.^53^ Over the course of the years, nanotechnology offered solutions to generate contrast agents able to yield high R2/R1 or R2*/R1 ratios.^54^ Very efficient routes to enhance R2/R2* values are provided by the synthesis and use of SPIO NPs.^55^ To address such challenges, novel magnetic iron oxide nanoparticles were synthesised *via* a co‐precipitation method and encapsulated with silica *via* a microemulsion coating method. Similar techniques have been used to synthesise uniformly sized and high quality, silica coated MNPs using a water‐in‐oil (W/O) microemulsion method.^56^,^57^ The formation of magnetic, crystalline Fe_3_O_4_ nanoparticles was confirmed by powder X‐ray diffraction (ICDD file no. 86‐2368, see ESI). The presence of silica was confirmed by FTIR vibrations at around 1100 cm^−1^ (Si−O symmetric stretch) and 820 cm^−1^ (asymmetric Si−O stretch)^58^. Magnetisation curves obtained by SQUID (Superconducting Quantum Interference Device) showed the typical characteristics of superparamagnetic behavior, zero coercivity and no remanence on hysteresis, for the NPs samples. The magnetisation (normalised per gram of sample) observed for Fe_3_O_4_@SiO_2_ (**A**) was one‐third of that measured for pure Fe_3_O_4_ MNPs. The core‐shell structure of the MNPs was confirmed by TEM (Figure 2). These MNPs have consistent, almost spherical morphology and the particles are well dispersed with sizes in the range of 5–10 nm for the iron oxide cores and 50–60 nm when encapsulated by silica. To address the optical imaging modalities, initial attempts to incorporate optical imaging agents focused on encapsulation of fluorescent organic dyes such as methylene blue MB, fluorescein FL, rhodamine B, Ru(bpy) into the silica shell and comparing their fluorescence efficiencies with the same NPs containing two classes of QD fluorophores; Lumidot 480 and Cd_0.1_Zn_0.9_Se nanocrystals.

Adapting the methods established by Wu *et al*.^64^ and Jacinto *et al*.,^61^ the fluorophores were incorporated hereby using a rapid and simple one‐step microemulsion method. TEM micrographs of such NPs are illustrated in Fig. S9‐S13 (ESI). However, none of the nanoparticles prepared using organic fluorophores exhibited significant luminescence. A reverse microemulsion method was employed to encapsulate Lumidot 480 QDs, resulting in core‐shell fluorescent Fe_3_O_4_/CdSe/ZnS@SiO_2_ (**B**) composite nanoparticles, the morphology of which can be seen by TEM (Figure S14). It is well known that QDs have higher extinction coefficients and quantum yields compared with organic dyes.^41^,^59^,^60^

**Figure 3:**
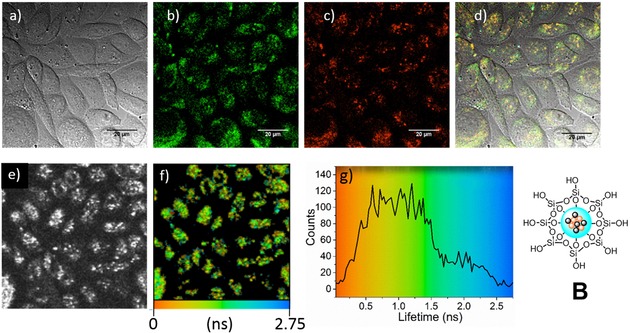
Laser‐scanning confocal microscopy (a‐d) of CHO cells treated with 10 μg/mL Fe_3_O_4_/CdSe/ZnS@SiO_2_ (**B**) in water, incubated for 15 minutes at 37 °C and two‐photon fluorescence lifetime imaging (e‐g) of the same area. a) DIC image; b) green channel; c) red channel; d) overlay of the green‐red channels; λ_ex_=488 nm; e–f) two‐photon fluorescence lifetime map; g) associated profile distribution. Colors provide a direct correlation between the lifetime maps and the lifetime histograms. Laser power: 2.0 mW at 910 nm wavelength. a–f) Scale bar: 20 μm; e and f show the same field of view.

Two‐photon laser scanning confocal microscopy was performed on Fe_3_O_4_/CdSe/ZnS@SiO_2_ (**B**) NPs in order to establish their potential as fluorescent imaging agents for *in vitro* cells and tissue studies. Chinese hamster ovary (CHO) cells were grown according to standard protocols, placed onto glass bottom dishes and allowed to grow to suitable confluence (see SI for cell culture and plating details). Figure 3a–d shows bright field confocal images, the overlay of green‐red channels and individual channel emissions (green: λ_em_=515–530 nm; red: λ_em_=605–675 nm). The images prove that, over a period of 15 minutes, the nanocomposite particles are taken up into cells and distributed across the cytoplasm with a large majority of emission lying in the green and red wavelength ranges. The fluorescence lifetime map distribution in CHO cells and the associated distribution profile of a water suspension of Fe_3_O_4_/CdSe/ZnS@SiO_2_ (**B**) are shown in Figure 3e–f and Figure 3g respectively. Fluorescence decays were fitted to two components. The major component (τ1, a1=88.2%) had a lifetime of 230 ps. A minor and longer second component (τ2=3216.5 ps, a2=11.8%) was also detected. As previously reported, the presence of a second longer component may suggest the presence of NP aggregates in suspension.^61^ However, the percentage of such secondary component, although significant, remains lower (11.8%) than that previously reported for nanocomposite materials (31.1%).^61^ These studies suggest, therefore, that simultaneous encapsulation of QDs and magnetic Fe_3_O_4_ nanocrystals into a SiO_2_ shell produces a nanocomposite material capable of being internalized and producing a stable fluorescent environment within cells. The predominance of the major component, measured by time‐correlated single photon counting (TCSPC) is also observed when the NPs are suspended in DMSO (τ1=24 ps, a1=97.3%; τ2=611.7 ps, a2=2.7%). The major component, τ1, is likely derived mainly from the core of the iron oxide nanocrystals. The difference in lifetimes suggests that the intra‐cellular environment may influence the aggregation of the fluorescent NPs within cellular compartments.^62^

### Encapsulation of Cadmium Quantum Dots into MNPs

Cadmium‐containing quantum dots are known to be toxic, which is in large part attributed to the presence of free Cd^2+^ ions released when non‐coated QDs are exposed to the acidic microenvironment after cellular uptake.^63^ Soenen and collaborators^64^ employed the degradation of cadmium quantum dots to correlate the loss of fluorescence intensity the number of cancer cells. Recent studies have shown that the toxicity of NPs can also be exploited as a novel means of providing cancer therapeutic effects. Pompa *et al*.^65^ reported that ion‐releasing NPs may induce high levels of cytotoxicity due to the so‐called lysosome‐enhanced Trojan horse effect. Furthermore, recent studies have demonstrated that the surface coating of QDs can substantially influence the toxicity of the particles.^66^ Particularly, Chu and collaborators demonstrated that the silica coating of CdSe QDs reduces the release of Cd ions by up to 99.45%.^67^

We also report the synthesis of a less‐common species of quantum dots, Cd_0.1_Zn_0.9_Se. Cd_0.1_Zn_0.9_Se nanocrystals were synthesised as reported by Knoll *et al*.^68^ by doping CdSe with Zn(II) cations and Se(0) stabilised in solution *via* organic phosphines.^[68],[69]^ Unlike the commercially available CdSe/ZnS QDs used above, in this QD system the Zn(II) and Se are not part of the protective shell of CdSe but are effective elements of the quantum dot core, capable of emitting simultaneously in the blue and red regions of the spectrum. Encapsulation of Fe_3_O_4_ and quantum dots with a silica shell to form Fe_3_O_4_/Cd_0.1_Zn_0.9_Se@SiO_2_ (**C**) proceeded *via* a microemulsion method. While Ying *et al*. reported the synthesis of the first QD doped MNPs encapsulated within silica,^32^ their method required 48 h for completion.

Our method (see ESI) produces effective nanomaterials suitable for optical biosensing in a considerably shorter period of time (16 h or less) which is fully adaptable to work under the confinements of a radiochemical laboratory.

Figure 2d shows a typical TEM micrograph of core‐shell Fe_3_O_4_/Cd_0.1_Zn_0.9_Se@SiO_2_ (**C**) NPs, indicating dimensions around 50–60 nm. Dynamic light scattering (DLS) showed that dispersions in chloroform Cd_0.1_Zn_0.9_Se and of Fe_3_O_4_, Fe_3_O_4_/Cd_0.1_Zn_0.9_Se@SiO_2_ (**C**) NPs in water had size distributions centered at 25, 45 and 300 nm respectively (Figure S23, ESI). Although the DLS suggests a small amount of aggregation in solution, it is known that this technique yields higher diameters than those observed by TEM.^70^,^71^,^72^ TEM and HRTEM demonstrate that the NPs have a core‐shell structure with a dark contrast metal core and a light contrast silica shell. The successful incorporation of Fe_3_O_4_ and Cd_0.1_Zn_0.9_Se into silica shell NPs was confirmed by optical spectroscopy and the presence of Fe, Zn, Cd and Se peaks (Figure S17–S20) in energy‐dispersive X‐ray (EDX) spectra. The atomic percentage of Fe and Se were 5.98% and 1.01%, respectively, which approximately corresponds to a 2:1 Fe_3_O_4_:Cd_0.1_Zn_0.9_Se ratio. The FTIR spectrum and magnetization curves are shown in Figure S22 (ESI).

**Figure 4:**
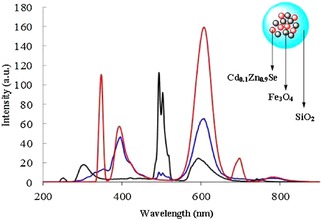
Fluorescence spectra of Cd_0.1_Zn_0.9_Se (hexane: λ_ex_=350 nm, red line; λ_ex_=252 nm, blue line) Fe_3_O_4_/Cd_0.1_Zn_0.9_Se@SiO_2_ (**C**) (methanol: λ_ex_=252 nm, black line).

Both the freshly synthesised Cd_0.1_Zn_0.9_Se and the corresponding nanocomposites Fe_3_O_4_/Cd_0.1_Zn_0.9_Se@SiO_2_ (**C**) exhibit fluorescence emission assignable to these QDs. Figure 4 shows the fluorescence spectrum of Cd_0.1_Zn_0.9_Se QDs recorded in dilute hexane dispersions (λ_ex_=350 nm and λ_ex_=252 nm) and Fe_3_O_4_/Cd_0.1_Zn_0.9_Se@SiO_2_ (**C**) in methanol dispersions. Free, non‐encapsulated QDs emit between 360–460 nm and 535–670 nm with λ_max_ at 395 nm and 607 nm, respectively. The relative fluorescence quantum yield (QY) for Cd_0.1_Zn_0.9_Se was determined before and after silica encapsulation and estimated with respect to anthracene in cyclohexane as a standard.^73^,^74^ The quantum yield of freshly prepared Cd_0.1_Zn_0.9_Se QDs was 0.15 (λ_ex_=350 nm) and 0.14 (λ_ex_=252 nm). It is interesting to note that Fe_3_O_4_ and SiO_2_ present simultaneously act as passivating agents for the Cd_0.1_Zn_0.9_Se nanocrystals.^11^,^75^,^76^,^77^ The fluorescence spectrum (λ_ex_=252 nm) of silica‐encapsulated Fe_3_O_4_/QDs shows the characteristic emission peaks in the red (552–687 nm) and blue (277–368 nm) regions with λ_max_ at 301 nm and 593 nm, respectively. UV‐Visible spectra of the QDs before and after silica encapsulation are shown in Figure S24 and S25 (ESI) respectively. The presence of Fe_3_O_4_ and the silica shell around Cd_0.1_Zn_0.9_Se decreased the QY. Indeed, for Fe_3_O_4_/Cd_0.1_Zn_0.9_Se@SiO_2_ (**C**) NPs the fluorescence was reduced by a factor of 100 (QY=0.0014). Despite showing a significantly reduced fluorescence QY, the Fe_3_O_4_/Cd_0.1_Zn_0.9_Se@SiO_2_ (**C**) NPs retained a luminescence emission profile which was traceable in dried thin films by confocal fluorescence microscopy.

**Figure 5:**
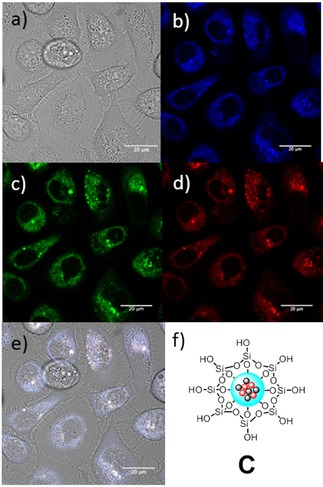
a–e) Single‐photon laser‐scanning confocal microscopy of PC‐3 cells incubated for 15 minutes with Fe_3_O_4_/Cd_0.1_Zn_0.9_Se@SiO_2_ (C) NPs. Final concentration: 10 μg/mL in 1:99 DMSO:serum free medium at 37 °C. a) DIC channel; b) blue channel (λ_em_=417–477 nm); c) green channel (λ_em_=500–550 nm); d); red channel (λ_em_=570–750 nm); e) overlapping of the DIC, blue, green and red channels. λ_ex_=405.0 nm. Scale bar: a–e) 20 μm.

Epi‐fluorescence microscopy (Figure S50a–e) was used for PC‐3 cells incubated with a DMSO:RPMI serum free medium (1:99) suspension (10 μg/mL) of Fe_3_O_4_/Cd_0.1_Zn_0.9_Se@SiO_2_ (**C**) in order to establish the potential of such NPs as cancer cell imaging agents. PC‐3 cells were grown according to standard serial passage protocols, plated onto glass bottom dishes and allowed to grow up to a suitable confluence (see SI for cell culture and plating details).The Fe_3_O_4_/Cd_0.1_Zn_0.9_Se@SiO_2_ (**C**) NPs are up taken in PC‐3 cells, with higher emission in the blue and green wavelengths and lower emission in the red wavelengths. Figure 5 shows confocal microscopy imaging of PC‐3 cells incubated for 15 minutes with Fe_3_O_4_/Cd_0.1_Zn_0.9_Se@SiO_2_ (C) NPs. Again, most emission was seen in the blue and green regions. However, there is broad emission across the visible spectrum when the probe is exited at 405 nm, while only green‐red emission is visible when excited with 488 or 561 nm lasers (Figure S47–S50). This is consistent with the fluorescence studies presented earlier where the Fe_3_O_4_/Cd_0.1_Zn_0.9_Se@SiO_2_ (**C**) NPs emitted blue and red wavelengths. The confocal microscopy images suggest that the Fe_3_O_4_/Cd_0.1_Zn_0.9_Se@SiO_2_ (**C**) NPs are located throughout the cell cytoplasm and no emission comes from the nuclear region of the cells. Therefore, we conclude that while the Fe_3_O_4_/Cd_0.1_Zn_0.9_Se@SiO_2_ (**C**) NPs are effectively internalised within the cell, they do not penetrate the nuclear membrane.

Cellular uptake of these nanocomposite particles was also conducted using a non‐cancerous fibroblast cell line, FEK‐4, (Figure S34–S38, ESI). No alteration in the morphology of the cells was apparent up to 6 hours after addition, a timescale far exceeding most common cellular imaging experiments in living cells.^78^,^79^ After 4 h incubation, a z‐stacking experiment showed that the NPs are evenly distributed throughout the cytoplasm in addition to adhering to the outer cellular membrane. It is frequently assumed that adherence to the cell surface may be the beginning of uptake *via* endocytosis; in our hands, however, internalisation was not observed within a standard time‐point imaging experiment (e. g. 1 h incubation at either 37 °C or 4 °C).

The biocompatibility of mesoporous silica NPs has been extensively studied in the past^92^ and as such it is known that the toxicity of silica NPs is a concentration‐dependent factor.^80^ While silica NPs are deemed biocompatible towards HeLa and CHO cells at concentrations below 100 μg mL^−1^, concentrations above 200 μg mL^−1^ are believed to cause cellular damage.^81^ Our experiments are carried out by using final concentrations (10 μg mL^−1^) that are 200 times lower that one resulting in cellular degradation. Furthermore, over the course of four hours, the morphology of FEK‐4 is largely unchanged. Such results and considerations seem to suggest that the concentration of the silica NPs used during our experiments are unlikely to produce cellular damage. Therefore, the internalization should not be considered as a result of cellular degradation.

The observation that these nanoparticles are fully dispersible and kinetically stable in aqueous media, yet do not appear to penetrate the cellular membrane over short timescale in such a typical (healthy) cell line is promising for their use in future bioimaging applications of gallium‐68. They are potentially valuable as imaging probes where it is desirable that nanoimaging agents do not enter healthy (non‐diseased) cells in an uncontrolled manner. They are stable over lifetimes compatible with the short half‐lives of gallium‐68 and with optical imaging experiments.

By encapsulating the Cd_0.1_Zn_0.9_Se nanocrystals in a thin silica matrix along with magnetic nanoparticles, we achieved a water dispersible system that retains the optical features of the free QDs as well as the magnetic properties of the iron oxide core. To the best of our knowledge, such NPs represent the first example of coated QDs which emit in two different and distinct regions of the spectrum. These fluorescent features potentially make such inorganic NPs a unique and versatile device for biological applications. Therefore, we explored the possibility that this may render these core‐shell materials as a tool for *in vitro* fluorescence imaging or ratiometric uses in environmental sensing.^30^

### Functionalisation of the MNPs Surface with a Known Hypoxia Tracer

**Figure 6:**
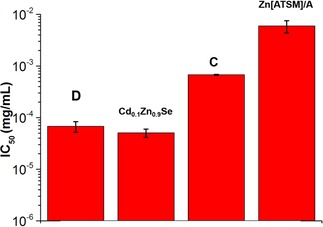
IC_50_ in PC‐3 cells after 48 hours treatment with Fe_3_O_4_@SiO_2_@Zn[ATSM]/A (D), Cd0.1Zn0.9Se and Fe_3_O_4_/Cd_0.1_Zn_0.9_Se@SiO_2_ (C) nanoparticles. (D) (IC_50_=6.77 ⋅ 10^−5^±1.60 ⋅ 10^−5^ mg/mL; Cd_0.1_Zn_0.9_Se IC_50_=5.07 ⋅ 10^−5^±9.40 ⋅ 10^−6^ mg/mL; (C) IC_50_=6.76 ⋅ 10^−4^±1.25 ⋅ 10^−5^ mg/mL; Zn[ATSM]/A=5.96 ⋅ 10^−3^±1.57 ⋅ 10^−3^ mg/mL.

The presence of a silica layer provides an opportunity for further functionalization being easily decorated with ligands such as biomolecules or drugs. Lu *et al*. reported that the monoclonal antibody to the β unit of human chorionic gonadotropin (anti‐β‐hCG) is adsorbed on the silica surface, adopting a “flat‐on” conformation at the interface.^82^ Moreover, silica is biocompatible and protects the iron oxide from degradation or aggregation, while eliminating the toxicity of the core, as proven by our MTT assays (Figure 6).^1^,^83^

To illustrate one possibility for exploiting this potential functionalization, we incorporated into the design metal‐ligand complexes that have potential selectivity for hypoxic tissue *in vivo* and *in vitro* and which were studied extensively in itsradiocopper bis(thiosemicarbazonato) forms. Functionalization methodologies of zinc bis(thiosemicarbazonato) complex precursors were reported: Dilworth *et al*.^84^ investigated the reaction of amino‐tagged bis(thiosemicarbazonato) complexes of the ligand denoted [ATSM]/A with α‐β‐D‐glucose as proof of principle of bioconjugation processes. To demonstrate that the outer surface of our Fe_3_O_4_@SiO_2_ (**A**) NPs can be functionalized *via* a condensation reaction, we investigated the reaction between Fe_3_O_4_@SiO_2_ (**A**) and [ATSM]/A metal complexes with a pendant amino group. For example, the amino group of Zn[ATSM]/A can react to form Si−N bonds at the surface. (Figure 1). Fe_3_O_4_@SiO_2_ (A) NPs and Zn[ATSM]/A were heated under reflux overnight in methanol. Single‐photon confocal microscopy images of PC‐3 cells incubated with Fe_3_O_4_@SiO_2_@Zn[ATSM]/A (D) NPs are shown in Figure S51–S52. While a homogeneous distribution of the particles is seen in the blue and green channels with 405 nm excitation, aggregates of the fluorescent probes are particularly visible in the green and red channels using 488 nm and 561 nm excitation. Such aggregates might be caused by a dynamic interaction between the particles and endosomes/lysosomes, as recently reported by Gu and collaborators.^62^

### Cellular Viability Tests

Standard MTT assays were performed in order to investigate the effect of silica encapsulation and surface functionalization on the cellular viability compared with pristine QDs (Figure 6). Fe_3_O_4_/Cd_0.1_Zn_0.9_Se@SiO_2_ (**C**) NPs have similar IC_50_ to cisplatin which is used to treat a variety of tumours (IC_50_=7.20 ⋅ 10^−4^ mg/mL measured over 72 h).^85^ The IC_50_ had lower (up to ten times) cytotoxicity (IC_50_=6.76 ⋅ 10^−4^ mg/mL) than the Cd_0.1_Zn_0.9_Se (IC_50_=5.07 ⋅ 10^−5^ mg/mL) nanocrystals (Figure 6). The functionalization of the silica shell lowers IC_50_ (6.77 ⋅ 10^−5^ mg/mL) from that of the unmodified surface. This might be caused by loss of metal cations. However, the results demonstrate that encapsulation of Cd_0.1_Zn_0.9_Se and F_3_O_4_ NPs within a silica shell improves the *in vitro* biocompatibility of the QDs used in this work.

### Radiolabelling of Nanocomposites using PET Isotopes ^64^Cu and ^68^Ga

The ability to functionalise the surface of the nanocomposite particles offers the possibility of supramolecular trapping or encapsulation of a radionuclide under the limited or no‐carrier‐added radiochemistry conditions. Various approaches have been used to devise radiolabelling protocols but this remains a synthetic challenge since the processes involved are under subtle kinetic control.

In this work, we demonstrate that it is possible to radiolabel the magnetic and fluorescent nanocomposites with both ^68^Ga and ^64^Cu radioisotopes *via* the general methods developed, in most cases *via* chelator‐free methods. ^68^Ga and ^64^Cu offer different advantages. They both undergo β^+^ decay, which allows *in vivo* PET imaging. ^64^Cu also has a characteristic low energy β^−^ emission which is effective for radiotherapy of tumour lesions with minimal effect on surrounding tissues.^[86],[87]^ Regarding their production, ^64^Cu is available through a cyclotron, whilst ^68^Ga can be obtained from a commercial generator.^[88],[89]^

**Figure 7:**
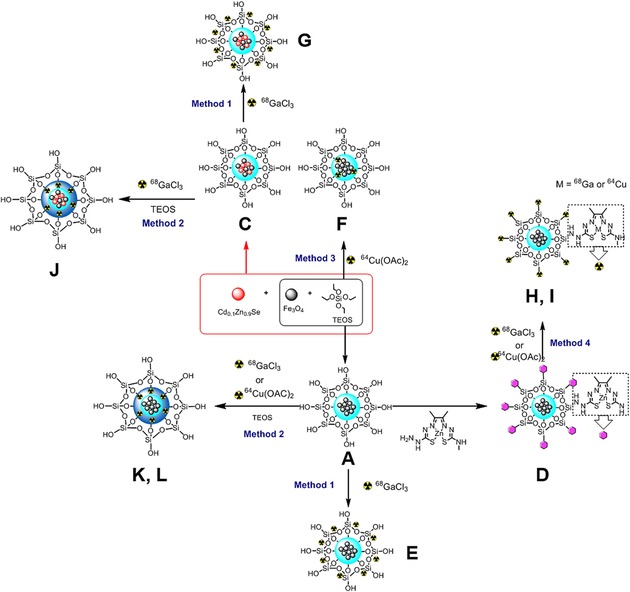
Synthesis and encapsulation methods for radiolabelled and non‐radiolabelled MNPs imaging probes.

To address radioactivity incorporation challenges, four radiolabelling methods were devised as summarised in Figure 7. **Method 1** involves non‐covalent adsorption of [^68^Ga]GaCl_3_ onto the silica surface of Fe_3_O_4_@SiO_2_ (**A**) and Fe_3_O_4_/Cd_0.1_Zn_0.9_Se@SiO_2_ (**C**). **A** and **C** NPs were dispersed in DMSO and heated to 95 °C for 40 min with the pH adjusted to 4.5. For all radiolabelling experiments, a sodium acetate buffer was used as an adjuvant to improve the encapsulation yield and to adjust the pH. The resulting magnetic nanomaterials were separated using a permanent magnet and centrifuged. The particles were then washed with methanol and water.

**Figure 8:**
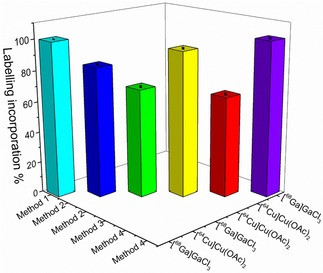
Optimised radio‐incorporation results emerging for each labelling method reported, using aqueous [^64^Cu]Cu(OAc)_2_ and [^68^Ga]GaCl_3_ precursors (see ESI).

The formation of a second silica shell around these nanocomposites which trapped the radionuclides was explored using **method 2** with [^68^Ga]GaCl_3_ and [^64^Cu]Cu(OAc)_2_. By following **method 2**, [^68^Ga]GaCl_3_ was added to a DMSO suspension of Fe_3_O_4_@SiO_2_ (**A**) or Fe_3_O_4_/Cd_0.1_Zn_0.9_Se@SiO_2_ (**C**) NPs with TEOS and cyclohexane. The resulting mixture was heated to 95 °C for 68 min. The added TEOS generated a second layer of silica trapping the radioisotope. The resulting radiochemical incorporation was found to be 70%, and due to the short half‐life of ^68^Ga, the reaction conditions could not be further optimised. However, the longer half‐life of ^64^Cu allowed us to validate the radiolabelling protocol in water at room temperature (see ESI). [^64^Cu]Cu(OAc)_2_ was incorporated as the radioactive material following **methods 2** and **3**. In order to optimise the radiolabelling protocols, six different parallel assembly routes were used (details are given in ESI). Different amounts of ^64^Cu radiotracer were added during the coating process at pH 8.

The longer half‐life of [^64^Cu]Cu(OAc)_2_ allowed for a 7 h, one‐pot encapsulation protocol (**method 3**) using Fe_3_O_4_, [^64^Cu]Cu(OAc)_2_ and TEOS. These three methods give a general approach which is also valid for other metal isotopes in aqueous media. The methods can be adjusted to suit the half‐life of the particular radionuclide, such as ^89^Zr (t_1/2_=78.4 h). While **methods 1**, **2** and **3** are based on the self‐assembly of the precursors within a silica network, we also investigated in **method 4** (details in ESI) the possibility of radiolabelling Fe_3_O_4_@SiO_2_@Zn[ATSM]/A (**D**) NPs *via* a transmetalation reaction involving Zn and the relevant radionuclide (e. g. ^68^Ga ions) in aqueous media.

Taking into account the half‐life of both radioisotopes, the decay‐corrected radiolabel incorporations were estimated. Over 99.9% incorporation was achieved from the non‐covalent adsorption of aqueous [^68^Ga]GaCl_3_ onto the silica surface. A value of 94% was achieved using [^64^Cu]Cu(OAc)_2_ in a one‐pot protocol (**method 3**), and 84% was obtained by following a step‐wise reaction with the same radioisotope (**method 2**). Using [^68^Ga]GaCl_3_ in **method 2** led to 70% incorporation of the radionuclide. The method using transmetalation of Fe_3_O_4_@SiO_2_@Zn[ATSM]/A (**D**) NPs (**method 4**) gave 65% with [^64^Cu]Cu(OAc)_2_ although with ^68^Ga the radiochemical incorporation increased to > 99.9%. However, this remarkable value may well be explained by a surface adsorption process which facilitates the radiochemical incorporation of [^68^Ga]GaCl_3_ as in **method 1**. The maximum efficiency was reached when ^68^Ga was injected into a suspension of silica coated NPs, following **method 1**. As a control, free Zn[ATSM]/A in ethanol was subjected to the same experimental conditions used for radiolabelling Fe_3_O_4_@SiO_2_@Zn[ATSM]/A (**D**) NPs. According to previous work by Pascu and co‐workers,^49^ the labelling of Zn[ATSM] complexes with ^68^Ga was unsuccessful. Therefore, we conclude that the covalent bond between the ATSM ligand and the silica surface plays a crucial role in the ^68^Ga radiolabelling process.

Virtually no loss of the radioactivity of the ^64^Cu radiolabelled nanoparticles dispersed in water was noted 7 h after encapsulation, showing that the encapsulated material does not leak out. The loading mechanism of ^64^Cu acetate (a well‐known monohydrate complex which adopts the paddle‐wheel structure^90^ of ca. 9 Å diameter) is likely based on solvophobic interactions reinforced by H‐bonds within the [SiO_2_]_n_ matrix formed in the microemulsion environment, efficiently trapping the radionuclide and resulting in leak‐free radioactive nanoparticles which are stable in aqueous media for weeks. After decay (3 weeks), TEM micrographs of the ^64^Cu radiolabelled MNPs were compared with freshly prepared, non‐radioactive nanoparticles. When ^64^Cu solution was added after the pre‐coating period (**method 2**), the shape of the core‐shell NPs was maintained (ESI) and no significant changes in shape and size or in aggregation were observed. These results demonstrate the possibility of synthesising a trimodal imaging agent which is highly stable with respect to radioactive agent loss. The results with the highest labelling incorporation percentage following each different radiolabelling method are reported in Table 1 and Figure 8.

**Table 1:** Summary of optimised radiolabelling methods and associated radio‐incorporation (%) with respect to precursors.

Procedure	Compound	Radioisotope	(%)
**Method 1** (Non‐covalent adsorption)	(**G**); (**E**)	[^68^Ga]GaCl_3_	>99.9±0.1
**Method 2** (Encapsulation of the radioisotope within a second layer of silica)	(**L**)	[^64^Cu]Cu(OAc)_2_	84±0.5
**Method 2** (Encapsulation of the radioisotope within a second layer of silica)	(**J**)	[^68^Ga]GaCl_3_	70±0.5
**Method 3** (One pot encapsulation reaction)	(**F**)	[^64^Cu]Cu(OAc)_2_	94±0.5
**Method 4** (Transmetallation reaction with Zn[ATSM]/A)	(**H**)	[^64^Cu]Cu(OAc)_2_	65±0.5
**Method 4** (Transmetallation reaction with Zn[ATSM]/A)	(**I**)	[^68^Ga]GaCl_3_	>99.9±0.1

## Conclusions

In conclusion, magnetic iron oxide nanoparticles incorporating Fe_3_O_4_ cores and Cd_0.1_Zn_0.9_Se quantum dots were simultaneously encapsulated within ca. 10 nm silica shell, giving rise to water‐dispersible, biocompatible and luminescent NPs with dimensions, ranging between 30–50 nm, which retained the magnetic properties. These were found to retain some of the emissive characteristics of the encapsulated QDs while still exhibiting magnetic properties due to the Fe_3_O_4_ core. Cellular interactions over a timescale of 30 min–6 h and incubation at 37 °C were visualised by epi‐ and confocal fluorescence microscopies. Surface modification allowed incorporation of two radiolabelled, Gallium‐68 and Copper‐64. The materials appear to be non‐toxic and constitute a promising, safe‐to‐handle and benign hybrid nanoparticles of potential utility as tri‐modal medical imaging probes that allow a simultaneous PET, MRI and fluorescence imaging. However, the optimization of the formulation for an effective and simultaneous use of these nanocomposites for multimodal imaging remains a challenge. While this study has focused mainly on new fine‐tuning encapsulation methods, preliminary experiments suggest that this approach can be extended to biologically or medically relevant nanoscaffolds and offers the potential for measuring the speciation of such systems in living cells. In future we envisage to extend this work towards other relevant PET radionuclides such as fluorine‐18.^91^

## **Experimental Section**

All manipulations were carried out by using standard Schlenk glassware and glove box techniques. All solvents were used as purchased and degassed by bubbling nitrogen for 30 min. Trioctylphosphine oxide (TOPO, 99%), trioctylphosphine (TOP, 90%), °octadecylamine (ODA, 90%), stearic acid (95%), diethylzinc (ZnEt_2_, 1.0 M solution in heptane), and Se powder (99.999%) were purchased from Aldrich. Cadmium stearate was purchased from Greyhound Chromatography. Zn[ATSM]/A was synthesised in accordance with a previously reported procedure.^84^ Further details of the experimental synthetic procedures used to synthesise compounds and materials mentioned in this work can be found in the supporting information. TEM micrographs, IR spectra, DLS, UV‐vis, 2D fluorescence spectra, as well as information regarding cell culture, MTT assays, epi‐fluorescence and confocal microscopy imaging are also given in the SI.

### Synthesis of Fe_3_O_4_ Nanoparticles

10 mL of 1 M FeCl_3_ were mixed with 2.5 mL of 2 M FeCl_2_ dissolved in 2 M HCl. Both solutions were freshly prepared with deoxygenated water before use. Immediately after being mixed under nitrogen, the solution containing iron chlorides was added to 125 mL of potassium hydroxide solution (0.7 M) under vigorous mechanical stirring and under a nitrogen atmosphere. After 30 min, the black precipitate formed was separated magnetically using a standard permanent magnet and washed with water (3×250 mL). Finally, oleic acid (5 mmol) was dissolved in 5 mL of acetone and was dropwise added.

### Coating of MNPs with a Silica Shell using Microemulsion Method

44.60 g of polyoxyethylene(5)isooctylphenyl ether (IGEPAL CA‐520) was dispersed in 700 mL of cyclohexane. Then, 200 mg of Fe_3_O_4_ nanoparticles dispersed in cyclohexane (20 mg mL^−1^) was added. The mixture was stirred until it became transparent. After this step, 9.44 mL of ammonium hydroxide (29% aqueous solution) was added to form a reverse microemulsion. Finally, 7.70 mL of tetraethylorthosilicate (TEOS) was added. The solution was gently stirred for 16 h. The nanocomposite was precipitated with methanol and separated by magnetic decantation.

### Synthesis of Cd_0.1_Zn_0.9_Se QDs

Stock solutions for Se and ZnEt_2_ were prepared in a glovebox under argon atmosphere. Cadmium stearate (0.2044 g, 0.3 mmol), stearic acid (0.1707 g, 0.6 mmol), TOPO (5.0 g), and ODA (5.0 g) were added to a flask, and the mixture was heated, under stirring, to 330 °C under a flow of argon until a clear solution formed. At this temperature, a solution containing 0.1184 g of Se (1.5 mmol) dissolved in TOP was injected into the reaction flask and the temperature was set to 290 °C. After 5–10 min. under stirring, the heating was removed to stop the reaction and allow the flask to cool to room temperature. After 1 h, the mixture of CdSe and organic ligands was heated up to 300 °C again. An aliquot (3 mL) of the as‐prepared crude CdSe reaction mixture, containing 0.1 mmol of CdSe, were transferred to a three‐neck Schlenk flask and heated at 300 °C. At this temperature, 0.450 mL of ZnEt_2_ (TOP solution, 0.2 M) and 0.450 mL of Se (TOP solution, 0.2 M) were injected. T the reaction mixture was heated for 6 min. Once the mixture reached room temperature, 9 mL of chloroform was added under stirring. Quantum dots were precipitated in a mixture 1:1 of methanol/acetone and isolated by centrifugation and decantation. A mixture of methanol/acetone (5×25 mL) was used to wash the QDs from the excess of organic ligands. Finally, Cd_0.1_Zn_0.9_Se nanocrystals were dispersed in 9 mL of n‐hexane and characterized by optical spectroscopy.

### Synthesis of Fe_3_O_4_/Cd_0.1_Zn_0.9_Se@SiO_2_ (C) NPs

In a typical experiment, 0.223 g of polyoxyethylene(5)isooctylphenyl ether was dispersed in 3.5 mL of cyclohexane. Then, 1.0 mg of Fe_3_O_4_ dispersed in cyclohexane (20 mg/mL) was added, followed by 50 μl of QD (1.50 mg/mL). The mixture was stirred until it became transparent. After this step, 45 μl of ammonium hydroxide (29% aqueous solution) was added to form a reverse microemulsion. Finally, 39 μL of TEOS was added. The solution was gently stirred for 16 hours. The nanocomposite was precipitated with methanol and separated by magnetic decantation.

### Synthesis of Fe_3_O_4_@SiO_2_@Zn[ATSM]/A (D)

30 mg of Fe_3_O_4_@SiO_2_ nanoparticles was dispersed in 100 mL of methanol, followed by 30 mg of Zn[ATSM]/A. The mixture was mechanically stirred and heated to 80 °C under reflux. After 16 h, the nanocomposite was separated by magnetic decantation and washed with further methanol (3×25 mL).

### Synthesis of ^68^Ga Radiolabelled MNPs

[^68^Ga]GaCl_3_ was produced and eluted through a ^68^Ge/^68^Ga generator in a saline/HCl (0.02 M) solution. *Method 1*. 50 μL of MNPs (A and C) (1 mg/mL DMSO stock solution) was dispersed in 0.4 ml of ethanol. 100 μL of [^68^Ga]GaCl_3_ stock solution (37 MBq in 500 μL) was added to the suspension, and the pH was adjusted to 5 by adding 1 mL of sodium acetate buffer solution (pH 4.5). The reaction was carried out for 40 min at 90 °C (using the vortex every 10 minutes). *Method 2*. 0.223 g of polyoxyethylene (5) isooctylphenyl ether (IGEPAL CA‐520) was dispersed in 3.5 mL of cyclohexane. Then, 30 μL of MNPs (A and C) (dispersed in DMSO (1 mg/mL)) was added and the mixture was stirred in a vortex. Next, 45 μL of ammonium hydroxide (29% aqueous solution) was added to form a reverse microemulsion, followed by 15 μL of TEOS. Finally, 50 μL [^68^Ga]GaCl_3_ stock solution was added to the reaction vial, the solution was heated to 90 °C and reacted for 68 min (sonicated every 10 min). *Method 4*. 50 μL of Fe_3_O_4_@SiO_2_@Zn[ATSM]/A (D) NPs (2 mg/mL DMSO stock solution) were dispersed in 0.4 mL of ethanol. 100 μL of [^68^Ga]GaCl_3_ stock solution (37 MBq in 500 μL) was added to the suspension, and the pH was adjusted to 5 by adding 1 mL of sodium acetate buffer solution (pH 4.5). The reaction was carried out for 40 minutes at 90 °C (using the vortex every 10 minutes). For all the experiments, the nanocomposite was transferred to centrifuging vials and washed with 0.5 mL of methanol and water (3×0.5 mL) and separated by magnetic decantation or using centrifugal filters. Each experiment was carried out three times.

### Synthesis of ^64^Cu Radiolabelled MNPs

[^64^Cu]CuCl_2_ was produced on a medical cyclotron using the ^64^Ni(p,n)^64^Cu nuclear reaction and purified using a using an ion‐exchange column. A stock solution of [^64^Cu]Cu(OAc)_2_ was prepared at pH 8.4, using 10 mM NaOAc solutions. *Method 2 and 3*. 0.223 g of polyoxyethylene (5) isooctylphenyl ether was dispersed in 3.5 mL of cyclohexane. Then, 1.0 mg of Fe_3_O_4_ (or A) dispersed in cyclohexane (20 mg/mL) was added. The mixture was stirred until it became transparent. After this step, 45 μL of ammonium hydroxide (29% aqueous solution) was added to form a reverse microemulsion, followed by 15 μL of TEOS. The reaction was carried out for 2 hours and subsequently, 25 μL of [^64^Cu]Cu(OAc)_2_ (aq) (from a 100 MBq stock solution) was added together with an additional 24 μL of TEOS. The solution was gently stirred for 5 hours. *Method 4*. 50 μL of D (1 mg/mL DMSO stock solution) were dispersed in 0.4 mL of H_2_O. 0.2 mL of [^64^Cu]Cu(OAc)_2_ (aq) solution were added to the suspension. The reaction was carried out for 40 minutes at 90 °C (using the vortex every 10 min). For all the experiments, the nanocomposite was precipitated with methanol (3×0.5 mL) and separated by magnetic decantation. Each experiment was carried out three times.

## Conflict of interest

The authors declare no conflict of interest.

## Supporting information

As a service to our authors and readers, this journal provides supporting information supplied by the authors. Such materials are peer reviewed and may be re‐organized for online delivery, but are not copy‐edited or typeset. Technical support issues arising from supporting information (other than missing files) should be addressed to the authors.

Supplementary
