# Supplementary material for: Synthesis, Radiolabelling and In Vitro Imaging of Multifunctional Nanoceramics
Source: ChemNanoMat. 2018 Feb 8;4(4):361–72. doi: 10.1002/cnma.201700378 (PMC5993288; doi:10.1002/cnma.201700378)
Supplement: Supplementary file 1 — Supplementary [file CNMA-4-361-s001.pdf]

Supporting Information

Synthesis, radiolabelling and *in vitro* imaging of multifunctional nanoceramics

Marina Lledos,[a] Vincenzo Mirabello, [a] Sophia Sarpaki,[a] Haobo Ge,[a] Hubert J. Smugowski,[a] Laurence Carroll,[b] Eric O. Aboagye,[b] Franklin I. Aigbirhio,[c] Stanley W. Botchway,[d] Jonathan R. Dilworth,[e] David G. Calatayud,[a,,f] Pawel K. Plucinski,[g] Gareth J. Price [a]* and Sofia I. Pascu [a]**

M. Lledos, Dr. V. Mirabello, Dr. D. G. Calatayud, S. Sarpaki, Dr. H. Ge, Dr. H. Smugowski, Prof. G. Price, Prof. S. I. Pascu
a. Department of Chemistry, University of Bath, Claverton Down, BA2 7AY, Bath, UK

Prof. E. O. Aboagye, Dr. L. Carroll
b. Department of Surgery and Cancer, Faculty of Medicine, Commonwealth Building, Hammersmith Campus, Imperial College London, Du Cane Road, London W12 0NN, UK

Prof. F. I. Aigbirhio
c. Wolfson Brain Imaging Centre, Department of Clinical Neurosciences, University of Cambridge, Cambridge, UK

Prof. S. W. Botchway
d. Central Laser Facility, Rutherford Appleton Laboratory, Research Complex at Harwell, STFC Didcot OX11 0QX, UK

Prof. J. R. Dilworth
e. Department of Chemistry, University of Oxford, South Parks Road, Oxford, OX1 3PA, UK

Dr. D. G. Calatayud
f. Department of Electroceramics, Instituto de Ceramica y Vidrio – CSIC, Kelsen 5, Campus de Cantoblanco, 28049, Madrid, Spain

Dr Pawel Plucinski
g. Department of Chemical Engineering, University of Bath, Claverton Down BA2 7AY, Bath, UK

Table of content

1. General Information

2. Experimental Section

3. TEM imaging and EDX

4. IR and Magnetisation curves

5. Dynamic Light Scattering (DLS)

6. UV-vis Spectroscopy

7. Fluorescence Spectroscopy

8. Cell Imaging and MTT assays

9. *In vitro* confocal imaging

10. Fluorescence-lifetime imaging microscopy

11. Kinetic stability tests

1. General Information

Characterisation of small molecule compounds was carried out using ^{1}H NMR and Mass Spectrometry. The ^{1}H NMR spectra were obtained on a 300 MHz Bruker Ultra Shield Spectrometer. All compounds for NMR analysis were dissolved in DMSO-d_6- and referenced according to the residual solvent peak. Mass spectrometry was carried out using the microTOF (ESI-TOF) at the University of Bath.

Powder X-ray diffraction

The analyses of the crystalline structure and the phase identification were performed by X-ray diffraction (XRD Bruker D8 ADVANCE, Madison, WI) with a monochromatized source of Cu Kα_1 radiation (λ = 1.5406 nm) at 1.6 kW (40 kV, 40 mA); samples were prepared by placing a drop of a concentrated ethanol dispersion of particles onto a single crystal silicon plate.

TEM/EDX

All images were taken on a JEOL JEM1200, a transmission electron microscope with an operating voltage ranging from 40 to 120 keV and all elemental analysis were carried out on an Oxford Energy Dispersive X-ray Spectrometer (EDS). All spectra were calibrated against a copper standard. In EDX, copper and nickel peaks were consistently observed between 7 and 10 keV as a result of the grid used to place the samples on.

***In vitro* fluorescence imaging**

The cells were cultured at 37 ºC in a humidified atmosphere in air and diluted once confluence had been reached. Culture occurred in Eagle's Minimum Essential Medium (EMEM) for FeK4, HeLa, CHO cells. The media contained foetal calf serum (FCS) (15% for FeK4), 0.5% penicillin/streptomycin and 1% L-Glutamine. Surplus supernatant containing dead cell matter and the excess protein was aspirated. The live adherent cells were then washed with 2 x 10 mL aliquots of phosphate buffer saline (PBS) solution to remove any remaining media containing FCS, which inactivates trypsin. Cells were resuspended in solution by incubation in 3 mL of trypsin-PBS solution (0.25% trypsin) for 5 min at 37 ºC. After trypsinisation, 5 mL of medium containing serum was added to inactivate the trypsin and the solution was centrifuged for 5 min (1000 rpm, 25 ºC) to remove any remaining dead cell matter. The supernatant liquid was aspirated and 5 mL of medium was added to the cell matter left behind. Cells were counted using a haemocytometer and then seeded as appropriate.

Cells were seeded as 10,000 cells per well and incubated for at least 48 h prior to microscopy. The wells were twice washed either PBS or SFM warmed to 37 ºC.
The working distance of a 60x objective lens is 0.17 mm (40x = 0.2 mm); therefore cells were seeded onto wells of which the coverslip was the bottom of the well. Wells were pre-coated with poly-d-lysine to equally maximise cell attachment and minimise detachment during washing steps.
PC-3 cells were cultured at 37 OC in 5 % CO_2 atmosphere and diluted once a suitable confluency had been obtained. PC-3 cells were cultured in RPMI 1640 containing 10 % heat activated foetal calf serum (FCS), 0.5 % penicillin/streptomycin (10000 mg/ml) and 200 mM L-glutamine. The medium contained no fluorescent indicator dyes such as phenol red and was therefore suitable for use in fluorescence imaging studies. The excess supernatant containing dead cell constituents and excess proteins and metabolites was aspirated. The viable live adherent cells were washed with 2x10 ml aliquots of PBS to remove residual media containing FCS. Cells were then re-suspended in 10 ml PBS with additional 2-5 ml Trypsin and incubated for a further 5 minutes at 37 OC. After trypsinisation, 5 ml of medium containing 10 % serum was added to inactivate the trypsin and the suspension centrifuged for 5 minutes (1000 rpm) to remove residual dead cell constituents. The resulting supernatant was aspirated and 5 ml of medium was added. Cells were counted using a haemocytometer and seeded as appropriate.
Fresh DMEM (10% FCS) was added to the suspended cells to give a sufficient concentration of cells (ca. 300000 cells/mL). The cells were plated in a Petri dish with a glass coverslip (MaTek) and left for 24 h to adhere before fluorescence imaging measurements were made.

Confocal Microscopy was performed using a Nikon A1Rsi Laser Scanning Confocal Microscope System fitted with 60X oil objective lens, equipped with three lasers (405.0, 488.0, and 561.0 nm). The microscope was also fitted with a motorized piezo z-stage, halogen lamp and mercury lamp for epifluorescence microscopy. All images were processed using functions within the NIS elements software package.
Two-photon excitation experiments were performed at the Rutherford Appleton Laboratory A mode locked Mira titanium sapphire laser (Coherent Lasers Ltd, USA), generating 180 fs pulses at 75 MHz and emitting light at a wavelength of 710–970 nm was used for the 2-photon excitation. The laser was pumped by a solid state continuous wave 532 nm laser (Verdi V18, Coherent Laser Ltd), with the oscillator fundamental output of 915 ± 2 nm or 810 ± 2 nm. The laser beam was focused to a diffraction limited spot through a water immersion

ultraviolet corrected objective (Nikon VC x60, NA1.2) and specimens illuminated at the microscope stage of a modified Nikon TE2000-U with UV transmitting optics. The focused laser spot was raster scanned using an XY galvanometer (GSI Lumonics). Fluorescence emission was collected and passed through a coloured glass (BG39) filter and detected by fast microchannel plate photomultiplier tube used as the detector (R3809-U, Hamamatsu, Japan). These were linked via a TCSPC PC module SPC830. Lifetime calculations were obtained using SPCImage analysis software (Becker and Hickl, Germany) or Edinburgh Instruments F900 TCSPC analysis software.

MTT assays

PC3 cells (3×103 per well) were seeded on a sterile 96 well plate and incubated for 48 hours to adhere. Coronene, NDI-Coronene, TRGO and NDI-TRGO were subsequently loaded at different concentration into wells and cultured for 48 hours. The Concentration used ranged between 250 µM (1% DMSO, 99% Eagle's Modified Essential Medium (10% FCS)) 100 µM, 50 µM, 10 µM, 1 µM, 0.5 µM, 100 nM and 1 nM. Each concentration was repeated six time in the experiment. Subsequently, cells were washed three times with PBS and 100 µL 3-(4, 5-dimethylthiazol-2-yl)-2, 5-diphenyltetrazolium bromide (MTT) was added (0.5 mg/ mL, 10% serum-free medium (SFM)) followed by a two-hour incubation. After aspiration, 100 µl of DMSO was added and 96 well plates were read by an ELISA plate reader, Molecular Devices Versa Max (BN02877). The absorption wavelength was at 570 nm and 630 nm wavelength was used as a reference.

Relative fluorescence quantum yields (QY) determination

UV/visible and fluorescence spectra were recorded on Perkin Elmer Lambda S50 UV/Vis and a Perkin Elmer LS55 Luminescence spectrometer, respectively. Room temperature fluorescence QY was calculated according to the following equation:

$$\Phi s = \Phi r * \frac{Ar}{As} * \frac{Es}{Er} * \frac{Ir}{Is} * \frac{n_s{}^2}{n_r{}^2}$$

In this equation subscripts *r* refers to the reference (anthracene), while *s* is referred to $Cd_{0.1}Zn_{0.9}Se$ and $Fe_3O_4/Cd_{0.1}Zn_{0.9}Se@SiO_2$. Φr and Φs are the fluorescence QY of anthracene (0.36), and unknown ($Cd_{0.1}Zn_{0.9}Se$ and $Fe_3O_4/Cd_{0.1}Zn_{0.9}Se@SiO_2$, respectively). A is the absorbance of the solution, E is the corrected emission intensity,

I is the relative intensity of the exciting light and n is the average refractive index of the solutions.

2. Experimental Section

All manipulations were carried out by using standard Schlenk glassware and glove box techniques. All solvents were used as purchased and degassed by bubbling nitrogen for 30 min. Trioctylphosphine oxide (TOPO, 99 %), trioctylphosphine (TOP, 90 %), octadecylamine (ODA, 90 %), stearic acid (95 %), diethylzinc ($ZnEt_2$, 1.0 M solution in heptane), and Se powder (99.999%) were purchased from Aldrich. Cadmium stearate was purchased from Greyhound Chromatography.

Synthesis of iron oxide nanoparticles for a microemulsion method of coating

10 ml of 1 M $FeCl_3$ were mixed with 2.5 ml of 2 M $FeCl_2$ dissolved in 2 M HCl. Both solutions were freshly prepared with deoxygenated water before use. Immediately after being mixed under nitrogen, the solution containing the iron chlorides was added to 125 ml of potassium hydroxide solution (0.7 M) under vigorous mechanical stirring, and under nitrogen atmosphere. After 30 min, the black precipitate formed was separated magnetically using a standard permanent magnet and washed with water (3×250 ml). Finally, oleic acid (5 mmol) was dissolved in 5 ml of acetone and was dropwise added. The crystal structure of the black powder was investigated by X-ray diffraction. **Figure S1** shows a crystalline single-phase pattern corresponding to Fe_3O_4 (ICDD file no. 86–2368) with no perceivable traces of other phases.

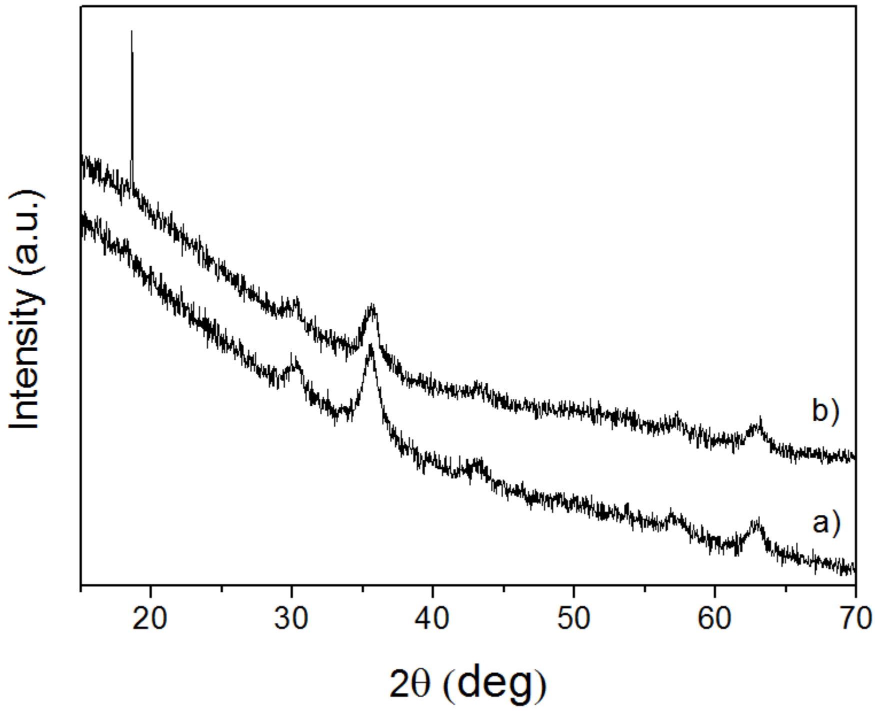

Figure S1. X-ray diffraction pattern for **a)** the synthesised Fe_3O_4 nanoparticles and **b)** $Fe_3O_4@SiO_2$ MNPs.

Coating of MNPs with a silica shell using microemulsion method

44.60 g of polyoxyethylene(5)isooctylphenyl ether (IGEPAL CA-520) was dispersed in 700 ml of cyclohexane. Then, 200 mg of Fe_3O_4 nanoparticles dispersed in cyclohexane (20 mg·ml^{-1}) was added. The mixture was stirred until it became transparent. After this step, 9.44 ml of ammonium hydroxide (29% aqueous solution) was added to form a reverse microemulsion. Finally, 7.70 ml of tetraethylorthosilicate (TEOS) was added. The solution was gently stirred for 16 h. The nanocomposite was precipitated with methanol and separated by magnetic decantation. The X-ray diffractogram of the obtained powder confirms that the Fe_3O_4 phase remains unaffected by the microemulsion method (**Figure S1b**).

Synthesis of dye-doped silica coated MNPs

0.223 g of polyoxyethylene(5)isooctylphenyl ether was dispersed in 3.5 ml of cyclohexane. Then, 1.0 mg of Fe_3O_4 dispersed in cyclohexane (20 mg·ml^{-1}) was added. The mixture was stirred until it became transparent. After this step, 45 µl of ammonium hydroxide (29 % aqueous solution), containing different amounts of dye **(**methylene blue 10 mg·ml^{-1}, fluorescein 20 mg·ml^{-1}, rhodamine B 10 mg·ml^{-1}, Rubpy 40 mg·ml^{-1}) was added to form a reverse microemulsion. Finally, 39 µl of TEOS was added. The solution was gently stirred for 16 h. The nanocomposite was precipitated with methanol and separated by magnetic decantation.

Synthesis of hydrophobic CdSe QDs (Lumidot CdSe 480) modified silica coated MNPs

0.223 g of polyoxyethylene(5)isooctylphenyl ether was dispersed in 3.5 ml of cyclohexane. Then, 1.0 mg of Fe_3O_4 dispersed in cyclohexane (20 mg·ml^{-1}) was added, followed by 50 µl of QD (Lumidot CdSe 5 mg·ml^{-1}). The mixture was stirred until it became transparent. After this step, 45 µl of ammonium hydroxide (29% aqueous solution) was added to form a reverse microemulsion. Finally, 39 µl of TEOS was added. The solution was gently stirred for 16 h. The nanocomposite was precipitated with methanol and separated by magnetic decantation.

Synthesis of $Cd_{0.1}Zn_{0.9}Se$ QDs

Stock solutions for Se and $ZnEt_2$ were prepared in a glovebox under atmosphere of argon (Ar). Cadmium stearate (0.2044 g, 0.3 mmol), stearic acid (0.1707 g, 0.6 mmol),

TOPO (5.0 g), and ODA (5.0 g) were added to a flask, and the mixture was heated, under stirring, to 330 °C under Ar flow until a clear solution formed. At this temperature, a solution containing 0.1184 g of Se (1.5 mmol) dissolved in TOP was injected into the reaction flask and the temperature was set at 290 °C. After 5-10 min. under stirring, the heating was removed to stop the reaction and allow the flask to cool to room temperature. After 1h, the mixture of CdSe and organic ligands was heated up to 300 °C again. An aliquot (3 mL) of the as-prepared crude CdSe reaction mixture, containing 0.1 mmol of CdSe, were transferred in a three-neck Schlenk flask and heated at 300 °C. At this temperature, 0.450 mL of $ZnEt_2$ (TOP solution, 0.2 M) and 0.450 mL of Se (TOP solution, 0.2 M) were injected. After the addition, the reaction mixture was heated for 6 min, and then heat was removed to stop the reaction. Once the mixture reached room temperature, 9 mL of chloroform was added under stirring. Quantum dots were precipitated in a mixture 1:1 of methanol/acetone and isolated by centrifugation and decantation. The same mixture of methanol/acetone (5×25 mL) was used to wash the QDs from the excess of organic ligands. Finally, $Cd_{0.1}Zn_{0.9}Se$ nanocrystals were dispersed in 9 ml of n-hexane and characterized by optical spectroscopy.

Synthesis of hydrophilic $Cd_{0.1}Zn_{0.9}Se$ QDs modified silica coated MNPs

In a typical experiment, 0.223 g of polyoxyethylene(5)isooctylphenyl ether was dispersed in 3.5 ml of cyclohexane. Then, 1.0 mg of Fe_3O_4 dispersed in cyclohexane (20 $mg \cdot ml^{-1}$) was added, followed by 50 µl of QD (water dispersible, 1.50 mg/ml). The mixture was stirred until it became transparent. After this step, 45 µl of ammonium hydroxide (29% aqueous solution) was added to form a reverse microemulsion. Finally, 39 µl of TEOS was added. The solution was gently stirred for 16 h. The nanocomposite was precipitated with methanol and separated by magnetic decantation.

Zn[ATSM]/A modified silica coated MNPs

30 mg of $Fe_3O_4@SiO_2$ nanoparticles were dispersed in 100 ml of methanol, followed by 30 mg of Zn[ATSM]/A. The mixture was mechanically stirred and heated to 80 °C under reflux. After 16 h, the nanocomposite was separated by magnetic decantation and washed with further methanol

Synthesis of ^{64}Cu modified and silica-coated MNPs

In this experiment, the positron-emitting radiotracer [^{64}Cu]Cu(OAc)$_2$ (prepared by Drs Paul Burke and Frank Aigbirhio from Wolfson Brain Imaging Centre in Cambridge using a standard method) was used in order to establish a general method to incorporate radioactive materials in silica shells. Different amounts of [^{64}Cu]Cu(OAc)$_2$ (from 100 µl of 100 MBq stock solutions at pH 8.4, using 10 mM NaOAc solutions as the support for the carrier-added radioactivity) was added (in different addition sequences) at several different stages during the coating process in order to optimize the encapsulation conditions, followed by aqueous washing and magnetic separation and evaluation of the radioactivity associated with the MNPs and residual phases. Taking into account the half-life (12.701 h) of the ^{64}Cu radionuclide, the radiolabeling yield, denoted here 'encapsulation factor', for all samples were estimated. The reaction conditions used and corresponding results are summarized in Tables S1 and S2 below. There is a difference between samples **a-c** (method 1) and **d-f** (method 2): smaller encapsulation factor can be simply explained by shorter time of reaction of [^{64}Cu]Cu(OAc)$_2$ in the reverse microemulsion system.

Table S1. Summary of the procedures for rapid preparation of ^{64}Cu-modified MNPs and reaction conditions for the 6 different approaches to encapsulation, resulting in Samples **a-f** after magnetic separation.

**Sample**	**a**	**b**	**c**	**d**	**e**	**f**
Cyclohexane / ml	3.5	3.5	3.5	3.5	3.5	3.5
IGEPAL 520 / g	0.223	0.223	0.223	0.223	0.223	0.223
Fe_3O_4 / µl	50	50	50	50	50	50
NH_4OH / µl	25	35	5	45	45	45
[^{64}Cu]Cu(OAc)$_2$ (aq) / µl	40	100	50	-	-	-
TEOS / µl	39	39	39	15	15	15
Time / hours	-	-	-	2	2	2
[^{64}Cu]Cu(OAc)$_2$ (aq) / µl	-	-	-	50	25	100
TEOS / µl	-	-	-	24	24	24
Total radiosynthesis time / hours	7	7	7	7	7	7

Table S2. Decay-corrected ^{64}Cu radiolabeling yield (denoted here as 'encapsulation' factor).

Experiment	a	b	c	d	e	f
Radio-incorporation factor	88%	91%	94%	53%	84%	78%

NMR Spectroscopy

Table S3. T1 and T2 values estimated by ^{1}H NMR for residual $Si(OH)_2$ and CH_3COONa resonances with respect to pure water for sample '**e**', estimated post-radioactive decay

Peak	T1	T2
5.129 ppm (δH_2O)	2.57 +/-0.06	0.026 +/-0.003
3.352 ppm ($\delta Si(OH)_2$)	3.18 +/-0.07	0.011 +/-0.001
0.916 ppm (δNaOAc)	3.55 +/-0.08	0.012 +/-0.002

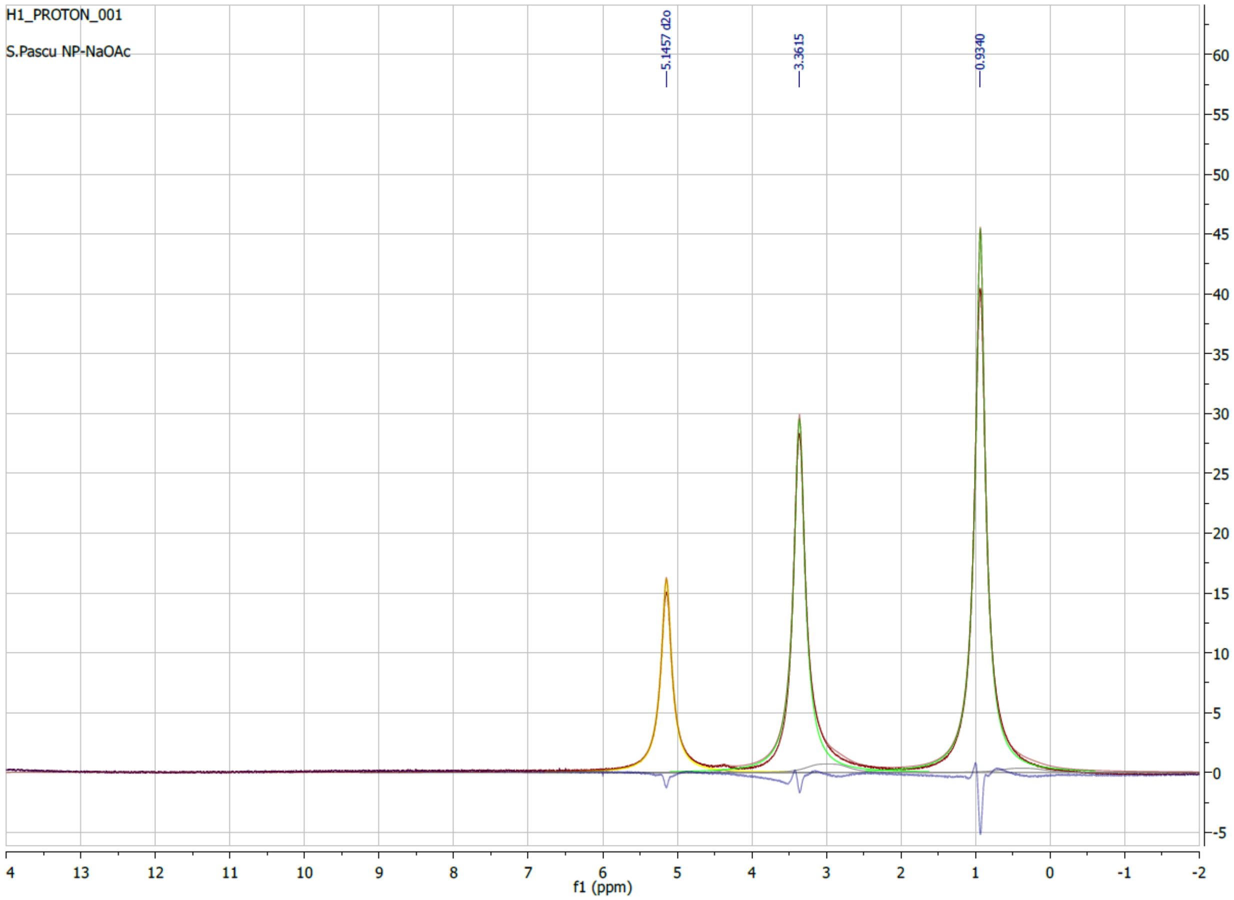

Figure S2.

Radiosynthetic protocols:

^{64}Cu was produced on a medical cyclotron using the $^{64}Ni(p,n)^{64}Cu$ nuclear reaction, and distributed in the form of $[^{64}Cu]CuCl_2$. In a typical encapsulation experiment (method 2 and 3), 0.223 g of polyoxyethylene (5) isooctylphenyl ether was dispersed in 3.5 ml of cyclohexane. Then, 1.0 mg of Fe_3O_4 dispersed in cyclohexane (20 mg/mL) was added. The mixture was stirred until it became transparent. After this step, 45 μL of ammonium hydroxide (29 % aqueous solution) was added to form a reverse

microemulsion, followed by 15 μL of tetraethylorthosilicate (TEOS). The reaction was carried out for 2 hrs and subsequently, 25 μL of [^{64}Cu]Cu(OAc)$_2$ (aq) (from a 100 MBq stock solution) was added together with an additional 24 μL of tetraethylorthosilicate (TEOS). The solution was gently stirred for 5 hrs. In a typical transmetallation experiment (method 4), 1.0 mg of Fe_3O_4@SiO_2 NPs containing Zn[ATSM]/A dispersed in DMSO (1 mg/mL) was added. The mixture was centrifuged and 50 μL of the precipitate was added in a Wheaton vial along with 0.2 mL of [^{64}Cu]Cu(OAc)$_2$ (aq) solution, and 0.4 mL of H_2O. The reaction was carried out for 45 min at room temperature (sonicated every 10 min). For all the experiments, the nanocomposite was precipitated with methanol and separated by magnetic decantation.

Table S4. Summary of the activity found under the different experiments regarding the transmetallation reaction (method 3) with [^{64}Cu]Cu(OAc)$_2$ (aq), and of the control experiments.

Sample	Initial Activity / MBq (time)	Corrected Activity / MBq (time)	Reaction Vial / MBq (time)	Waste vial / MBq (time)	ROI / %
Fe_3O_4@SiO_2@Zn[ATSM]/A	5.03 (19:46)	4.79 (20:41)	3.1 (20:41)	1.7 (20.42)	65
Fe_3O_4@SiO_2	5.33 (18:21)	4.64 (20:54)	2.37 (20:54)	2.2 (20.52)	51
Fe_3O_4@SiO_2@Zn[ATSM]/A	5.44 (18:20)	5.13 (19:24)	0.34 (19:24)	4.84 (14:15)	0.07

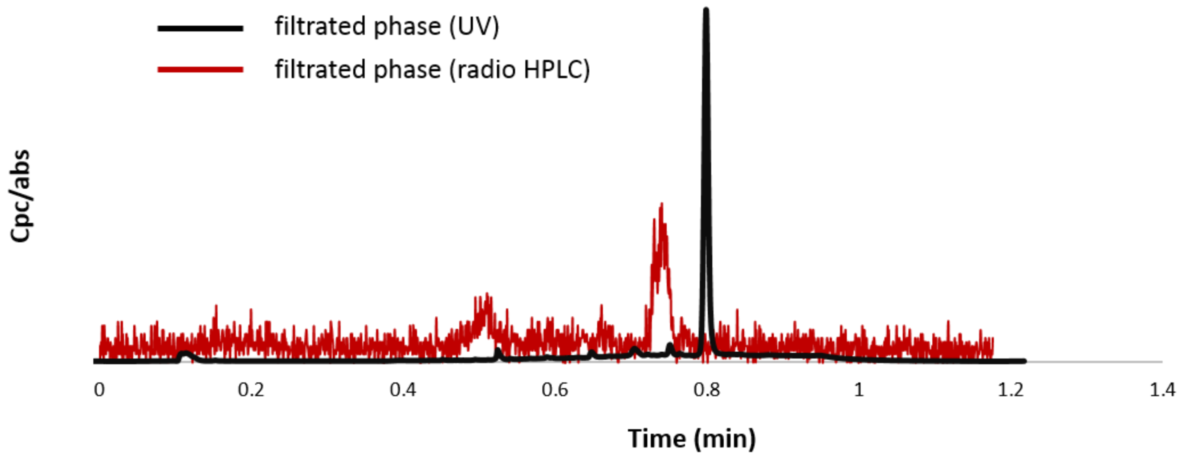

Figure S3. Radio and UV HPLC chromatograms of Fe_3O_4@SiO_2@[^{64}Cu]CuATSM/A in the filtrated phase.

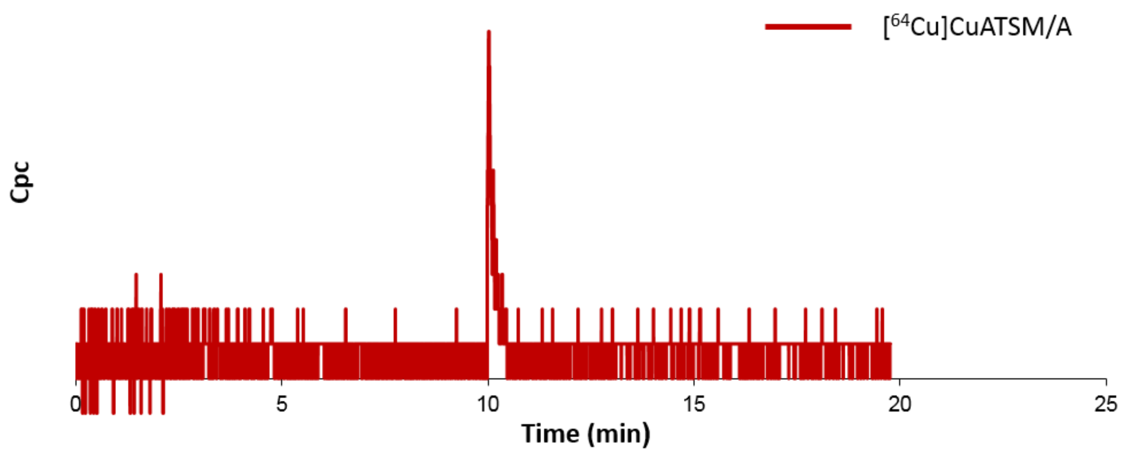

Figure S4. Radio HPLC chromatogram of the [^{64}Cu]CuATSM/A precursor used.

Synthesis of ^{68}Ga-modified and silica-coated MNPs

The positron emitting radiotracer ^{68}Ga(III) (prepared at the Department of Surgery and Cancer of Imperial College in London) was obtained using either a TiO_2 or a SnO_2-based column matrix ^{68}Ge/^{68}Ga generator. These experiments were carried out in order to confirm the general method established above for incorporation of radioactive Ga (^{68}Ga) in the silica shell of iron oxide nanoparticles. Taking into account the half-life (67.629 min) of the ^{68}Ga(III) radionuclide, the radiolabeling incorporation was estimated for all samples, denoted here 'encapsulation factor'. Because of its relatively shorter half-life and its different character, the reaction times and the buffer solution used were adjusted accordingly. The reaction conditions used and corresponding results are summarized in **Tables S5** and **S6** below. There is a difference between samples **a-b** (method 1), **c-d** (method 2) and **e** (method 4): the first two experiments regard the non-covalent adsorption of ^{68}Ga onto the surface of the silica network of the $Fe_3O_4@SiO_2$ and $Fe_3O_4/Cd_{0.1}Zn_{0.9}Se@SiO_2$ NPs; the next two involve the encapsulation reaction of the radioactive isotope using two different types of nanoparticles, whilst the last one regards the reaction of ^{68}Ga in a $Fe_3O_4@SiO_2$ NPs system containing Zn[ATSM]/A.

Table S5. Summary of the procedures of rapid preparation of ^{68}Ga (III) modified MNPs and reaction conditions.

**Sample**	**a**	**b**	**c**	**d**	**e**
Cyclohexane / ml	-	-	3.5	3.5	-
$Fe_3O_4@SiO_2$ / µl	-	-	-	30	-
$Fe_3O_4/Cs_{0.1}Zn_{0.9}Se$ @SiO_2/ µl	-	-	30	-	-
TEOS / µl	-	-	24	24	-
$^{68}Ga(III)$ / µl	100	1000	50	50	100
IGEPAL CA-520 / g	-	-	0.223	0.223	-
NH_4OH / µl	-	-	45	45	-
$Fe_3O_4@SiO_2@$ Zn[ATSM]/A / µl	50	50	-	-	50
NaOAc buffer (pH 4.5) / µL	100	100	-	-	100
Ethanol / µL	400	400	-	-	400
Total radiosynthesis time / min	40	40	68	68	40

Table S6. Decay-corrected radiolabeling yield (denoted here as the encapsulation, or radio-incorporation, factor).

Experiment	a	b	c	d	c
Radio-incorporation factor	>99.9%	>99.9%	70%	66%	>99.9%

^{68}Ga was eluted through the generator in a saline/HCl (0.02 M) solution. In a typical non-covalent radiolabeling experiment (method 1: **a,b**) 100 µl $^{68}Ga(III)$ of a 37 MBq (in 500 µl) stock solution were added to a Wheaton vial along with 0.4 ml of ethanol ,0.1 ml of buffer solution [(pH 4.5) in order to adjust the pH to approximately 5] and 50 µL of MNPs (1 mg/mL DMSO stock solution).

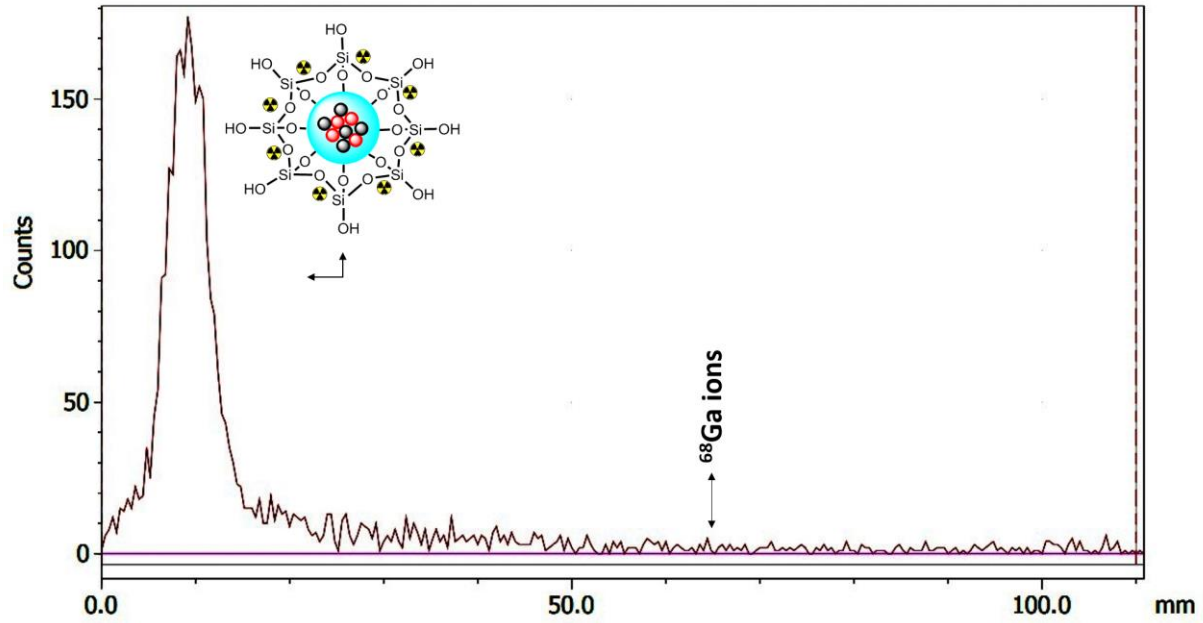

Figure S5. Radio-TLC chromatogram of labelling $Fe_3O_4/Cd_{0.1}Zn_{0.9}Se@SiO_2$ with $^{68}Ga(III)$ suggesting an ROI of > 99.9%. TLC was carried out using 0.25 M ethylenediaminetetraacetic acid (EDTA) as the mobile phase.

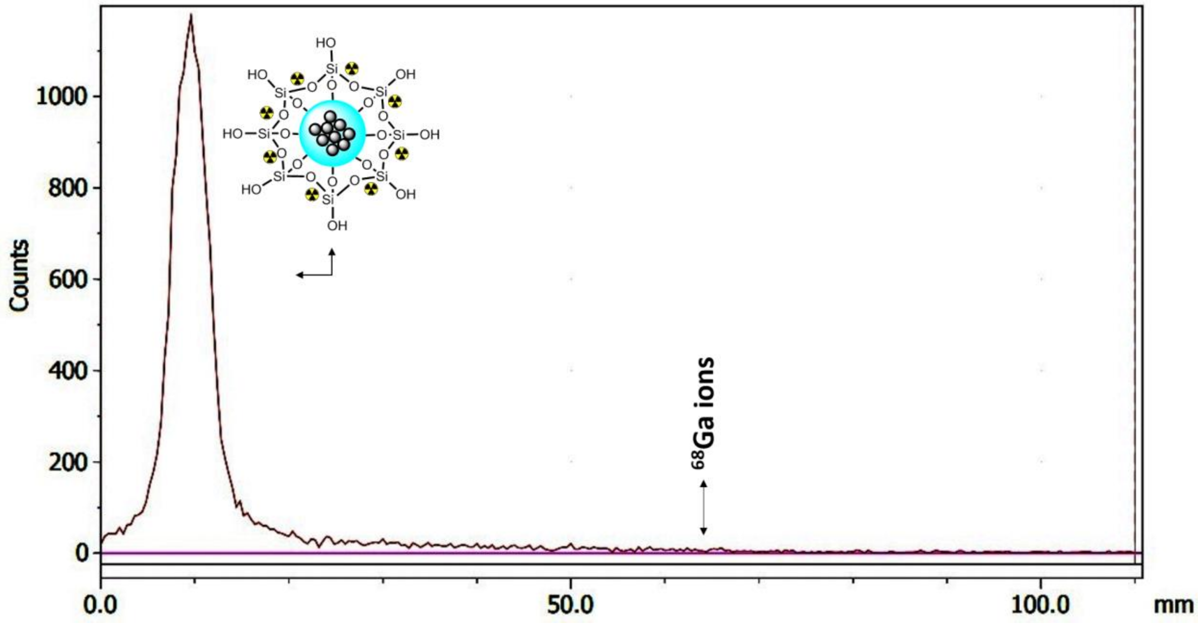

Figure S6. Radio-TLC chromatogram of labelling $Fe_3O_4@SiO_2$ with $^{68}Ga(III)$ suggesting an ROI of > 99.9%. TLC was carried out using 0.25 M ethylenediaminetetraacetic acid (EDTA) as the mobile phase.

The reaction was carried out for 40 min at 90 °C (using the vortex every 10 min). In a typical encapsulation experiment (method 2: **c,d**), 0.223 g of polyoxyethylene (5) isooctylphenyl ether (IGEPAL CA-520) was dispersed in 3.5 ml of cyclohexane. Then, 30 μl of NPs (dispersed in DMSO (1 mg/ml)) were added and the mixture was stirred

in a vortex. Next, 45 μl of ammonium hydroxide (29 % aqueous solution) was added to form a reverse microemulsion, followed by 15 μl of tetraethylorthosilicate (TEOS). Lastly, 50 μl ^{68}Ga from a 37 MBq (in 500 μl) stock solution was added in the reaction vial and the solution was heated to 90 °C and reacted for 68 min (sonicated every 10 min). For experiment **e** 1.0 mg of $Fe_3O_4@SiO_2@Zn$[ATSM]/A NPs dispersed in DMSO (1 mg/ml) was added. The mixture was centrifuged and 50 μl of the precipitate was added to a Wheaton vial along with 0.1 ml of ^{68}Ga solution, 0.4 ml of ethanol and 0.1 ml of buffer solution (pH 4.5) in order to adjust the pH to approximately 5. The reaction was carried out for 40 min at 90 °C (using the vortex every 10 min). For all the experiments, the nanocomposite was transferred to centrifuging vials and washed once with methanol and three more times with water.

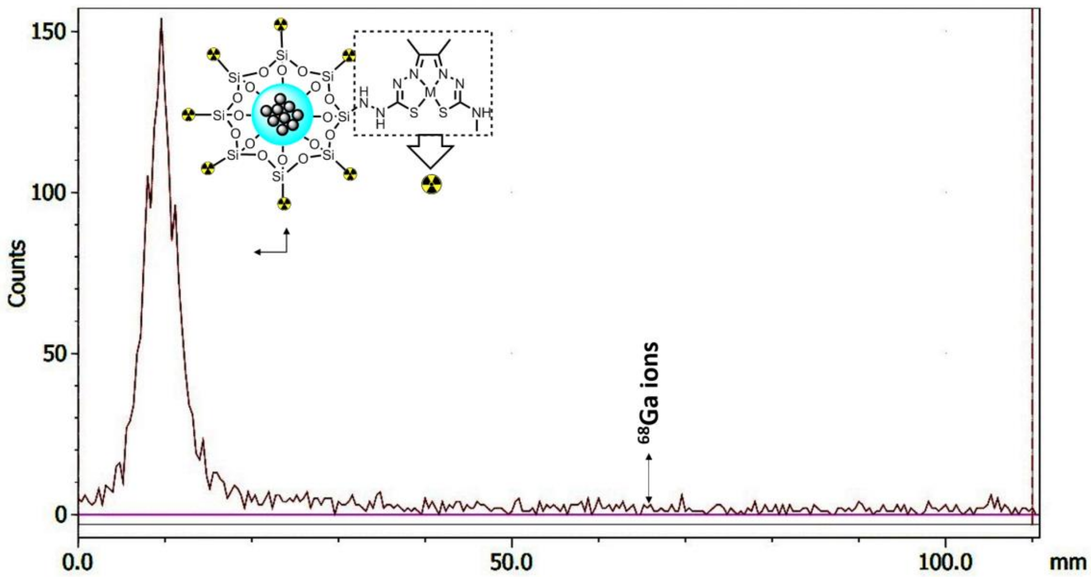

Figure S7. Radio-TLC chromatogram of labelling $Fe_3O_4@SiO_2@Zn$[ATSM]/A with ^{68}Ga(III) suggesting an ROI of > 99.9%. TLC was carried out using 0.25 M ethylenediaminetetraacetic acid (EDTA) as the mobile phase.

As reported in the manuscript, the radiochemical incorporation of ^{68}Ga(III) in $Fe_3O_4@SiO_2@Zn$[ATSM]/A (**D**) NPs by following method 4 yielded >99%. However, the radiolabelling ATSM/A with Ga-68 has never been achieved before, and the surface absorption may have contributed to the incorporation of the radioisotope. We also undertook a control experiment by exposing the non-anchored free Zn[ATSM]/A to the same experimental conditions reported for method 4. The reaction mixture was monitored by radio-HPLC, where only Ga-68 was traced (Figure S8). This experiment

confirmed that the radiolabelling ATSM/A ligand with Ga-68 is not achievable. Thus, the covalent attachment of ATSM onto the silica surface is crucial for the ^{68}Ga radiolabelling process.

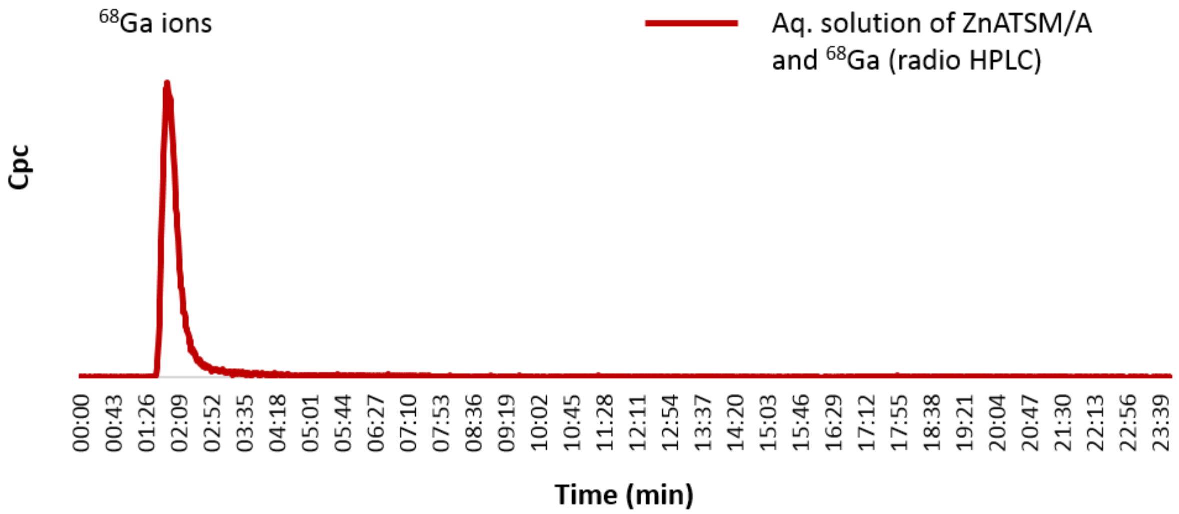

Figure S8. Radio-HPLC chromatogram of the aqueous solution formed from the reaction between ^{68}Ga(III) and ZnATSM/A. From this trace, no product could be identified except from the free hydrated ^{68}Ga ions present at approximately 2 min.

Synthesis of metal(thiosemicarbazone) complexes

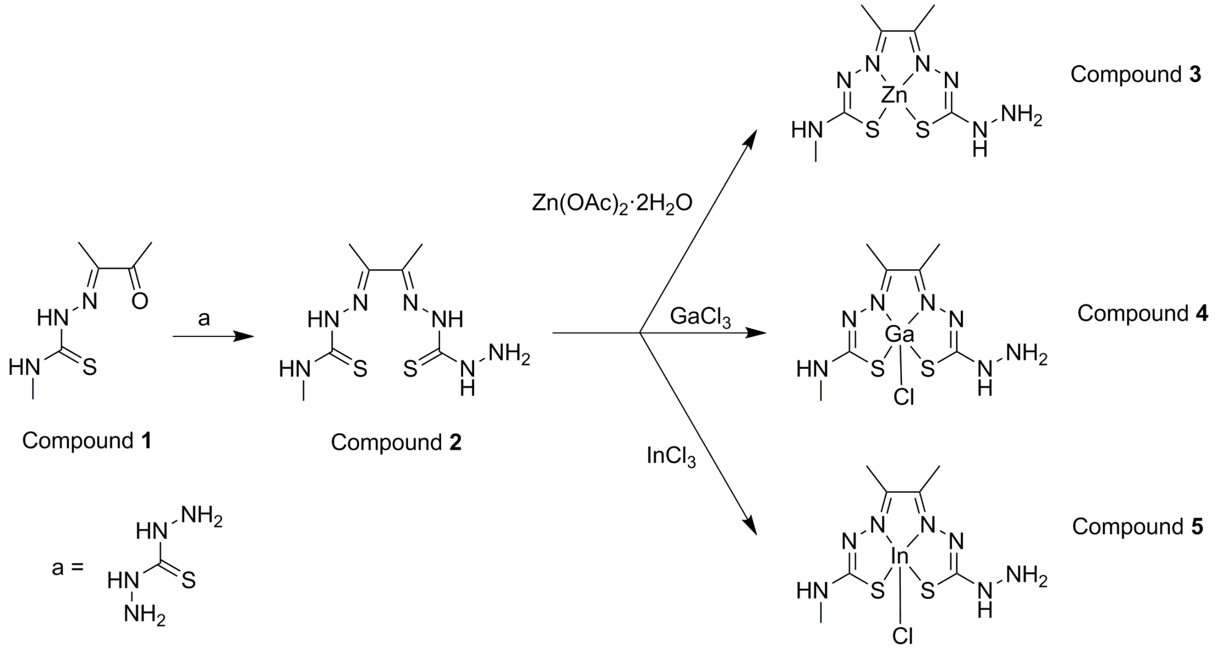

Scheme S1. Synthesis of Compounds **3**–**5**.

General synthesis

Compounds **1-3** were synthesised using a method described by Dilworth *et al.* [*Inorg. Chem.* **2007**, *46*, 465−485]. For Compounds **4** and **5**, the synthesis was simply adapted to suit the change in metal centres (e.g. Ga and In), the reactions are depicted in **Scheme S1**. Compound **2** was reacted with the zinc, gallium and indium reagents in the appropriate ratios as described in the experimental section, in methanol, refluxed for four hours and then collected by filtration. For compounds **4** and **5** the reagents $GaCl_3$ and $InCl_3$ were weighed out in a glove box to prevent hydrolysis or H_2O complexation of the anhydrous reagents used.

Upon the addition of $Zn(OAc)\cdot 2H_2O$ to the Compound **2** there was an immediate colour change to yellow, which was retained in the solid product for Compound **3**. For Compound **4** a bright orange solid was collected and a further fraction isolated as a brown/orange oil. For Compound **5** again a small amount of solid was collected and a second fraction was obtained, an orange/brown oil from the filtrate.

Synthesis of ligands and metal complexes

Adapted synthesis of Compound 2

Step (1):

4-methyl thiosemicarbazone (1.25 g, 11.9 mmol) was added to 100 ml of deionised water and vigorously stirred at 0 ºC. 5 drops of concentrated HCl was then added followed by the rapid

addition of 1.2 equivalents of 2,3-butadione (1.23 g, 14.3 mmol). The yellow solution became opaque as a white precipitate formed. After 1 hour the precipitate was collected by filtration and washed with water (3 x 30 ml). The solid was dried overnight in the air and then further dried in vacuo to give the desired species. Yield = 68%

^{1}H NMR: (300MHz, d^6-DMSO, 25ºC) δ 10.60 (s, 1H, NN*H*), δ 8.62 (s, 1H, CH_3NH), δ 3.05 (dd, 3H, CH_3NH), δ 2.42 (s, 3H, $CH_3C=O$), δ 1.96 (s, 3H, C*H*3C=N)
ES MS: M/z = 195.9 $[M + Na]^+$

Step (2):
Thiocarbohydrazide (0.3664 g, 3.5 mmol) was added to 20 ml of ethanol and the suspension stirred at 50 ºC. 1 equivalence of compound emerging from Step (1), above, (0.6059 g, 3.5 mmol) was slowly added in portions over 2 hours to the suspension. After the final addition, 5 drops of 10% HCl was added and the reaction refluxed for five hours during which time a cream/white suspension formed. The reaction mixture was allowed to cool to room temperature and the precipitate was then collected by filtration, washed with ethanol (2 x 30 ml) and diethyl ether (5 x 30 ml). The solid was then dried in vacuo to give Compound **2**. Yield = 82%

^{1}H NMR: (300MHz, d^6-DMSO, 25ºC) δ 10.50 (s, 2H, $NHNH_2$), δ 9.65 (s, 1H, $NHNH_2$), δ 8.35 (s, 1H, $NHCH_3$), δ 4.98 (s, 2H, N*H*N=CC=NN*H*), δ 3.0 (d, 3H, CH_3NH), δ 2.70 (s, 6H, CH_3CCCH_3)
ES-MS: M/z = 284.1 $[M + Na]^+$

Synthesis of Compound 3
1.2 equivalents of Zinc (II) diacetate dihydrate (0.2122 g, 0.81 mmol) were added to a stirred suspension of Compound 2 in 30 ml of methanol, which caused the reaction mixture to turn yellow immediately. The mixture was then heated under reflux for four hours. A yellow/orange precipitate formed, which after cooling was collected by filtration, washed thoroughly with 100 ml of hexane and then dried in vacuo. Yield = 45%

^{1}H NMR: (300MHz, d^6-DMSO, 25ºC) δ 8.24 (s, 1H, NH_2NH), δ 7.20 (s, 1H, CH_3NH), δ 4.45 (s, 2H, $NHNH_2$), δ 2.83 (d, 3H, $NHCH_3$), δ 2.22 (d, 6H, CH_3CCCH_3)
ES MS: M/z = 324.0 $[M]^+$

Synthesis of Compound 4

3 equivalents of $GaCl_3 \cdot 6H_2O$ (0.5436 g, 1.9 mmol) were weighed out into a round-bottom flask. Due to the air sensitivity of this reactant, the Gallium compound was weighed out in a glove box. To this reaction mixture compound 2 (0.1916 g, 0.74 mmol) was added with 30 ml of methanol. This was added as quickly as possible due to the air sensitivity of the Gallium compound. The mixture was then left to reflux for four hours. An orange precipitate formed which after cooling was collected by filtration. The filtrate was reduced under vacuum, diethyl ether was added and solid scratched off which could then be filtered and left with a solid that was then dried in vacuo. The little solid that was collected from the original filtration was washed with THF, transferred to a round-bottom flask and reduced under vacuum leaving an oil which was then dried in vacuo to give a thicker oil. Solid yield = 43%

Fraction collected from filtrate:

^{1}H NMR: (300MHz, d^6-DMSO, 25ºC) δ 10.25 (s, 1H, NH_2NH), δ 10.19 (s, 1H, CH_3NH), δ 8.40 (m, 2H, NH_2NH), δ 2.94 (d, 3H, $NHCH_3$), δ 1.9 (D, 6H, CH_3CCCH_3)

ES MS: M/z = 368.02 $[M+H]^+$, M/z = 368.02 $[M+Cl]^+$

Fraction collected from solid:

^{1}H NMR: (300MHz, d6-DMSO, 25ºC) δ 10.32 (s, 1H, NH_2NH), δ 10.24 (s, 1H, CH_3NH), δ 5.76 (d, 2H, NH_2NH), δ 2.95 (d, 6H, CH_3CCCH_3)

ES MS: M/z = 551.02 $[2M -2CH_3,-2NH_2-2NH]^+$

Synthesis of Compound 5

5 equivalents of $InCl_3 \cdot 6H_2O$ (0.641 g, 1.9 mmol) were weighed out into a round-bottom flask. Due to the air sensitivity of this reactant, the Indium compound was weighed out in a glove box. To this reaction mixture Compound 2 (0.100 g, 0.38 mmol) was added with 30 ml of methanol. This was added as quickly as possible due to the air sensitivity of the Indium compound. The mixture was then left to reflux for four hours. The reaction mixture was filtered, again leaving very little solid, this was collected with THF, transferred to a round-bottom flask and concentrated under vacuum before drying in vacuo leaving a solid. The filtrate was reduced under vacuum and an oil was left which was then dried in vacuo.

^{1}H NMR: (300MHz, d^6-DMSO, 25ºC) δ 10.8 (s, 1H, $NH_2N\mathit{H}$), δ 9.9 (s, 1H, $CH_3N\mathit{H}$), δ 5.00 (m, 3H, $N\mathit{H}_2NH$), δ 3.02 (m, 3H, $C\mathit{H}_3CCCH_3$), δ 2.20 (m, 3H, $CH_3CCC\mathit{H}_3$), δ 2.10 (m, 3H, $NHC\mathit{H}_3$)

ES MS: M/z = 372.98 $[M+H]^+$, M/z = 409.63 $[M+Cl]^+$, M/z = 685.07 $[2M-2NH_2-2CH_3]^+$

Synthesis of Compound 6

Compound 3 acted as a precursor to the analogous copper complex in a transmetallation reaction. One equivalent of Compound 3 (0.034 g, 0.12 mmol) and two equivalents of copper acetate (0.044 mg, 0.24 mmol) were stirred in a methanol under reflux for 24 hours and the product was isolated by filtration.

^{1}H NMR: (300MHz, d6-DMSO, 25ºC) No observable peaks due to the paramagnetic nature of Cu(II)

ES MS: M/z = 304.25 $[M-NH_2]^+$

Synthesis of Copper(II) (thiosemicarbazone) complex

Compound **6** was synthesised using a transmetallation reaction from the analogous Compound **3**. Two equivalents of $Cu(OAc)_2$ were added to Compound **3** in methanol and then stirred under reflux for 24 hours. As Compound **3** was the precursor to this reaction and Compound **3** had not been produced in any considerable quantity very little solid was collected from this reaction.

3. TEM imaging and EDX analysis of organic and inorganic compounds-dopped silica NPs and MNPs

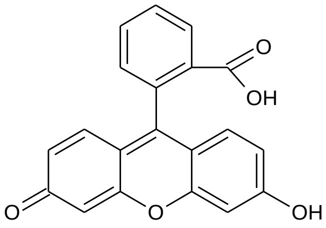

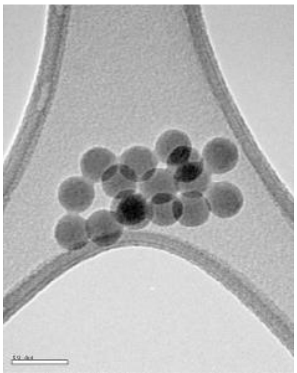

a) b)

Figure S9. a) Structural representation of fluorescein (FL), b) TEM micrograph of Fluorescein-doped silica NPs. Scale bar: 50 nm.

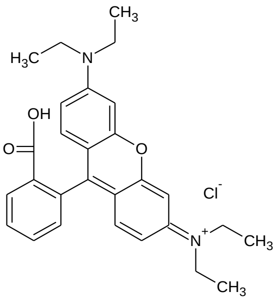

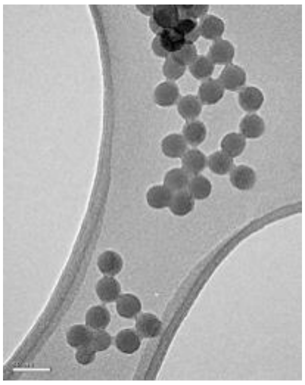

a) b)

Figure S10. a) Structural representation of rhodamine B, b) TEM micrograph of rhodamine B doped silica NPs. Scale bar: 30 nm.

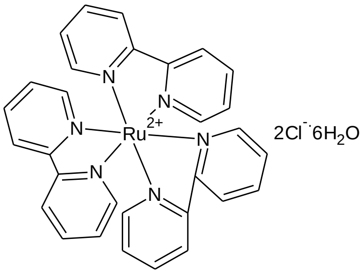

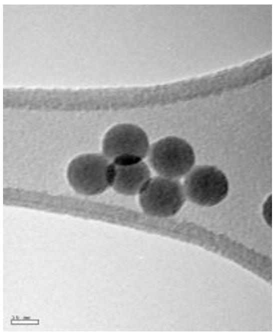

a) b)

Figure S11. a) Structural representation of [tris(2,2′-bipyridyl)ruthenium (II) chloride hexahydrate)] (Rubpy), b) TEM micrograph of Rubpy-doped silica NPs. Scale bar: 50 nm.

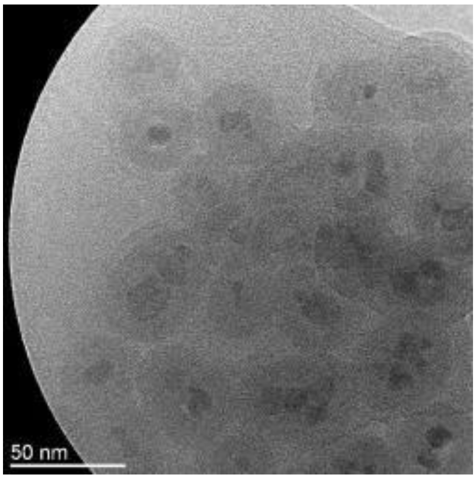

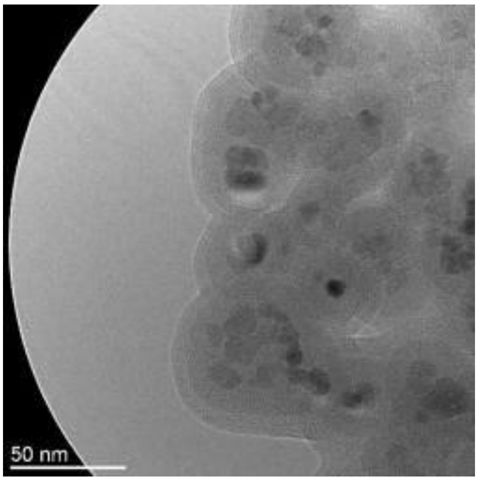

a) **b)**

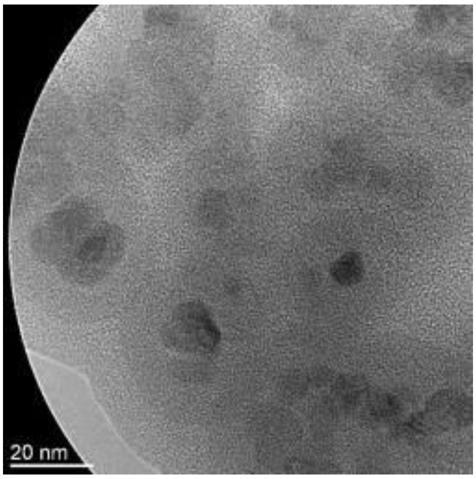

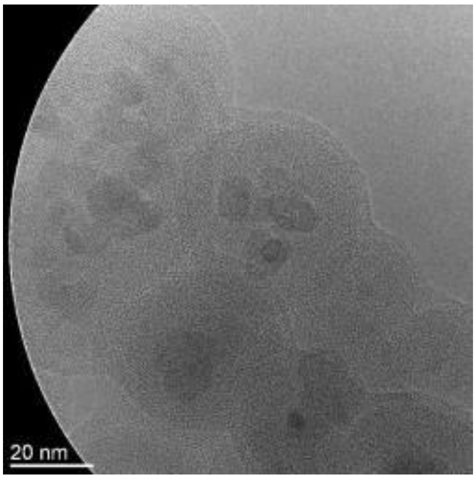

c) **d)**

Figure S12. HR TEM micrographs of ammonium perrhenate-doped silica coated MNPs. Scale bar: a-b) 50 nm; c-d) 20 nm.

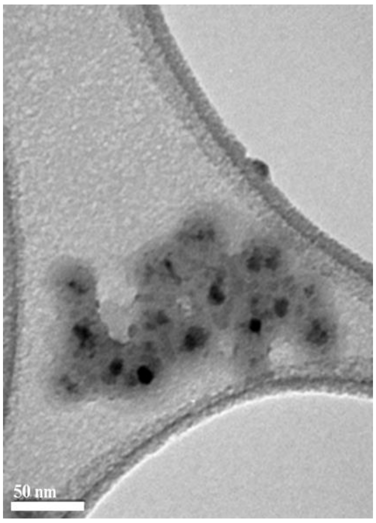

Figure S13. TEM micrograph of MNPs which comprise Fe_3O_4 cores, coated with silica and doped with a commercial CdSe QDs (Lumidot CdSe 480, Sigma-Aldrich). Scale bar: 50 nm.

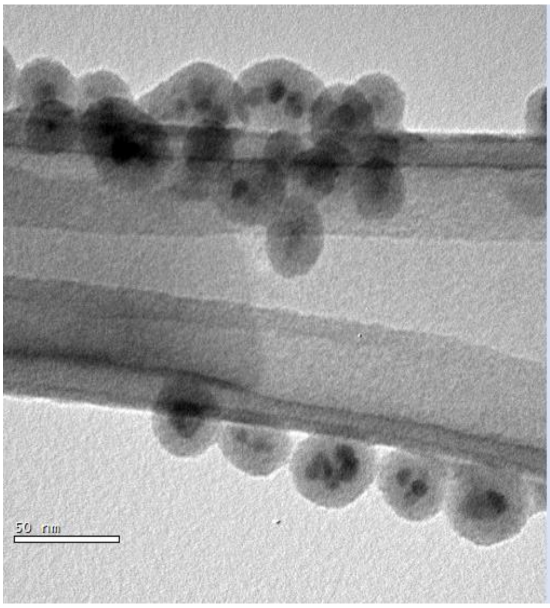

Figure S14. TEM micrograph of MNPs with the composition Fe_3O_4/CdSe/ZnS@SiO_2 recorded from fresh ethanol dispersions. Scale bar: 50 nm.

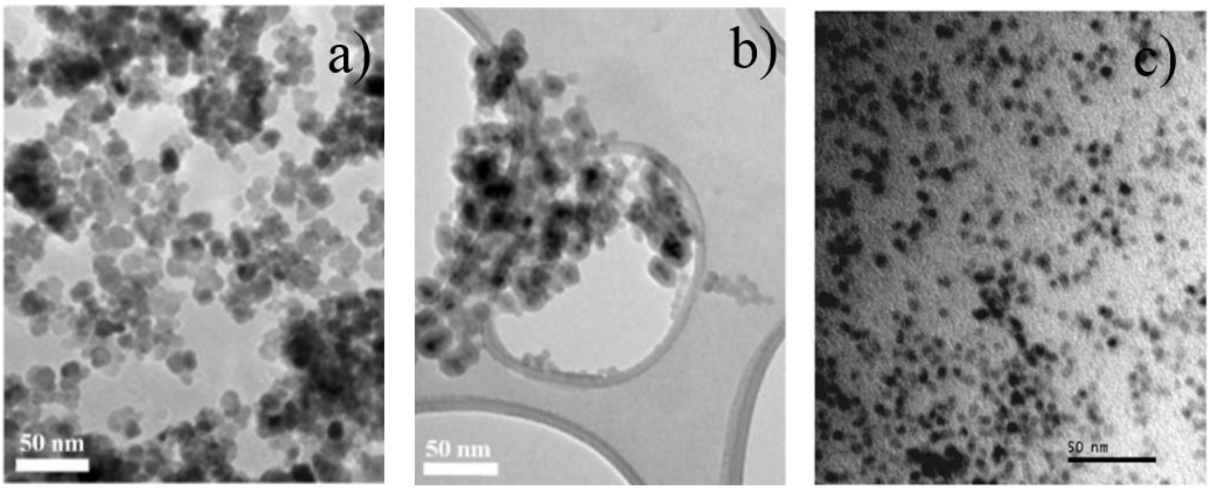

Figure S15. TEM micrographs of the building blocks: a) Fe_3O_4 nanoparticles, b) Fe_3O_4@SiO_2, and c) Fe_3O_4/$Cd_{0.1}Zn_{0.9}Se$@SiO_2. Scale bar: 50 nm.

In comparison to the unchanged reverse microemulsion method, the addition of aqueous solution of $^{64}Cu(OAc)_2$ affects the size and shape and agglomeration state of resulting nanocomposites, likely due to the loss of water/oil equilibrium in reverse microemulsion. These MNPs were imaged post-decay after a 3 weeks period in an aqueous environment. The samples **a-c** appeared to be rather aggregated. Better results were obtained when $^{64}Cu(OAc)_2$ solution was added after the pre-coating period (samples **d-f**). There is a clear difference between nanoparticles **d-f**.

The best quality nanoparticles (sample **e**) were obtained when the smallest amount of the

aqueous phase containing $^{64}Cu(OAc)_2$ stock solution was added (25 µl containing 25 MBq activity, 84% radiochemical incorporation in the ‘chelate-free’ method).

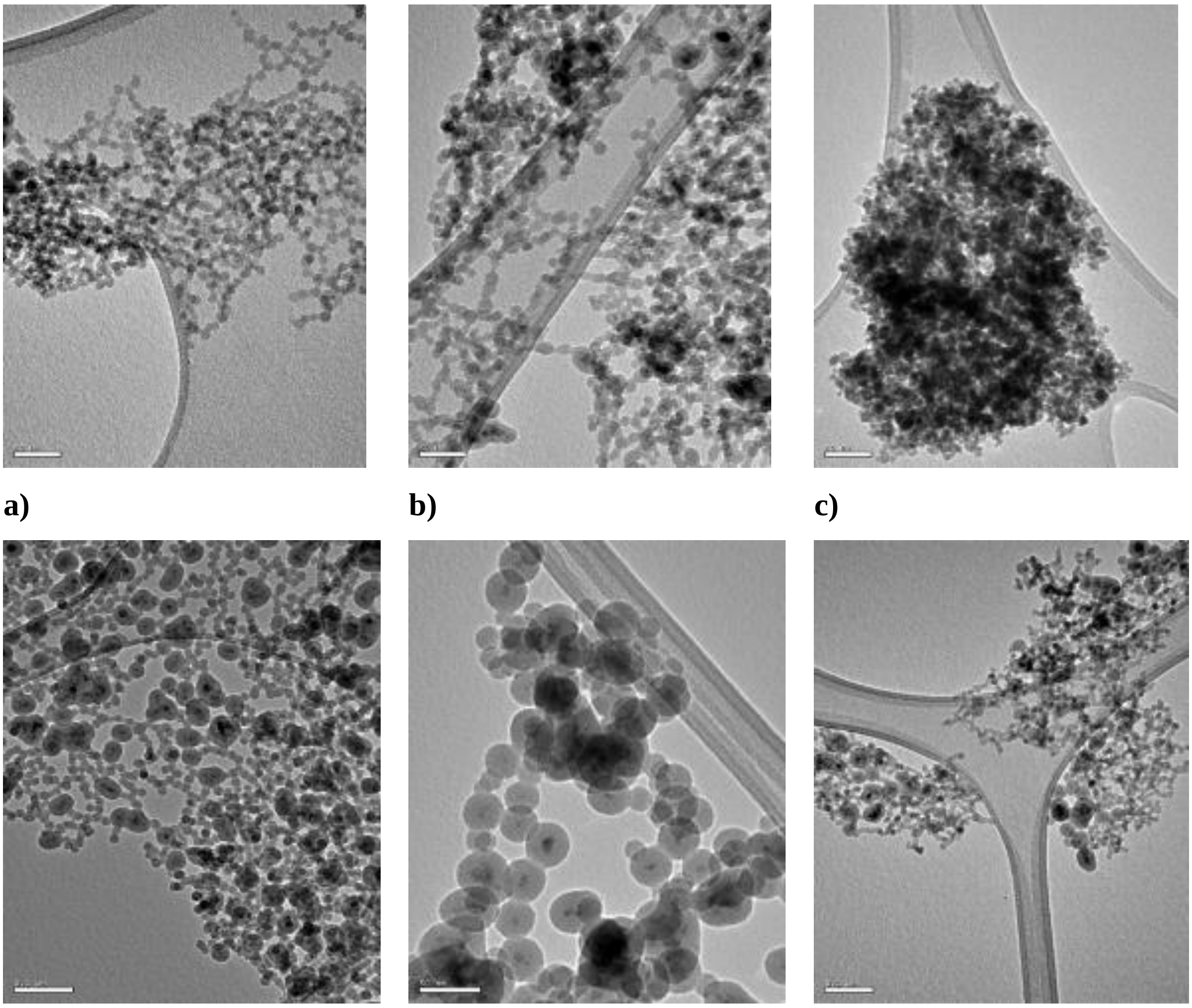

a) **b)** **c)**

d) **e)** **f)**

Figure S16. TEM micrographs of silica coated MNPs recorded post decay from samples 9a)-(f) generated with the addition of $^{64}Cu(OAc)_2$ under ‘hot’ conditions. Images were recorded 3 weeks after the self-assembly from building blocks, and post-radioactive decay.

Energy-dispersive X-ray spectroscopy (EDX) for the elemental analysis in the chemical characterization of MNPs

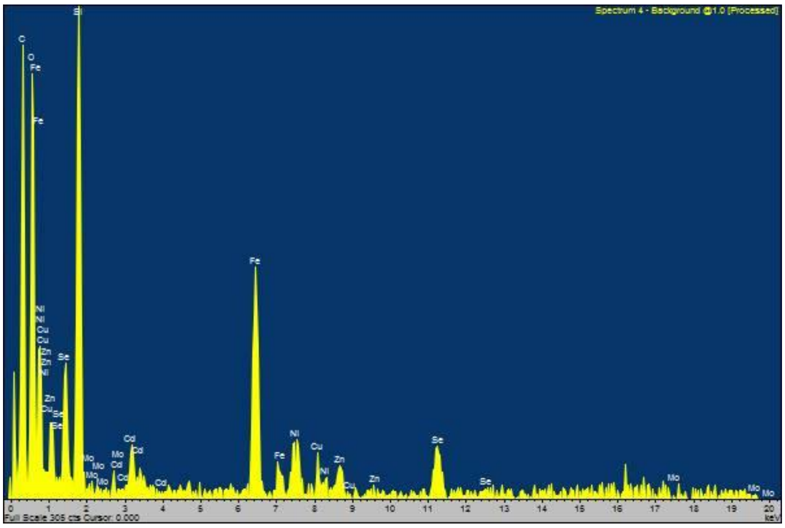

Figure S17. EDX spectrum of $Fe_3O_4/Cd_{0.1}Zn_{0.9}Se@SiO_2$ (Spectrum 1 in Table S7).

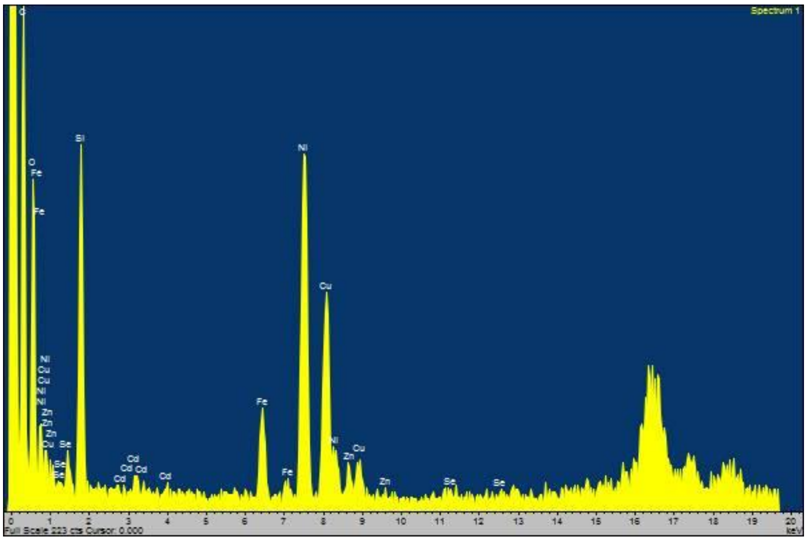

Figure S18. EDX spectrum of $Fe_3O_4/Cd_{0.1}Zn_{0.9}Se@SiO_2$ (Spectrum 2 in Table S7).

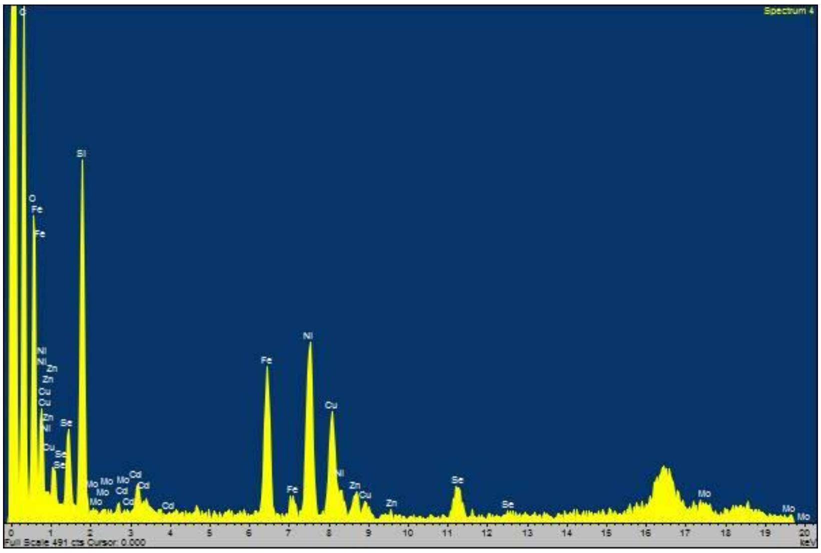

Figure S19. EDX spectrum of $Fe_3O_4/Cd_{0.1}Zn_{0.9}Se@SiO_2$ (Spectrum 3 in Table S7).

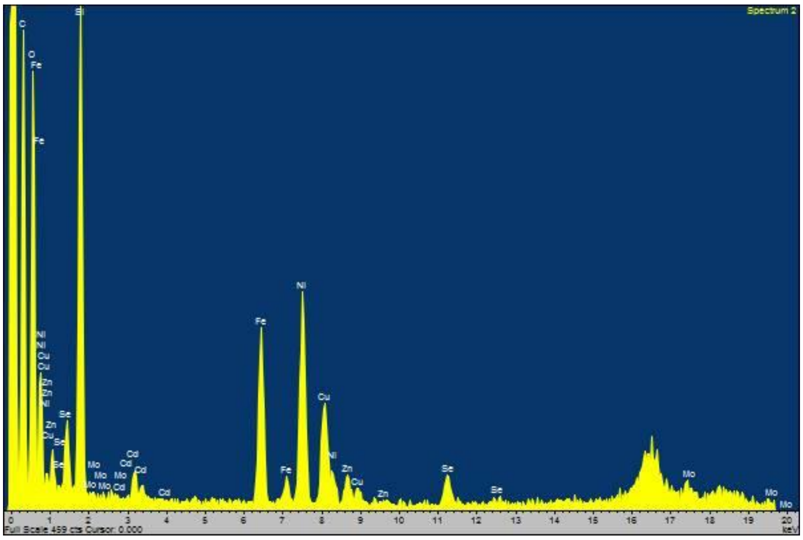

Figure S20. EDX spectrum of $Fe_3O_4/Cd_{0.1}Zn_{0.9}Se@SiO_2$ (Spectrum 4 in Table S7).

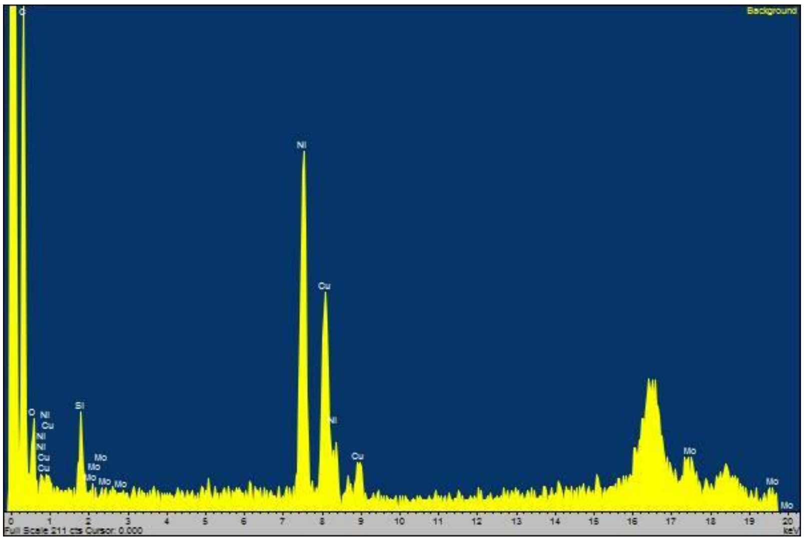

Figure S21. EDX spectrum of TEM grid background (Spectrum 5 in Table S7).

Table S7. EDX-measured atomic percentages (%) of $Fe_3O_4/Cd_{0.1}Zn_{0.9}Se@SiO_2$

Spectrum	C	O	Si	Fe	Ni	Cu	Zn	Se	Mo	Cd
1	53.59	29.53	6	5.98	1.46	0.3	1.72	1.01	0.02	0.38
2	60.39	15.53	3.65	1.52	10.47	7.23	0.94	0.14		0.14
3	59.33	21.68	4.68	3.1	5.72	3.54	1.21	0.45	0.02	0.27
4	65.21	17.33	3.52	2.84	5.53	3.57	1.16	0.57	0.04	0.23
5 (background)	73.63	4.16	0.84		12.49	8.83			0.05	

4. IR spectroscopy and magnetisation curves of representative MNPs

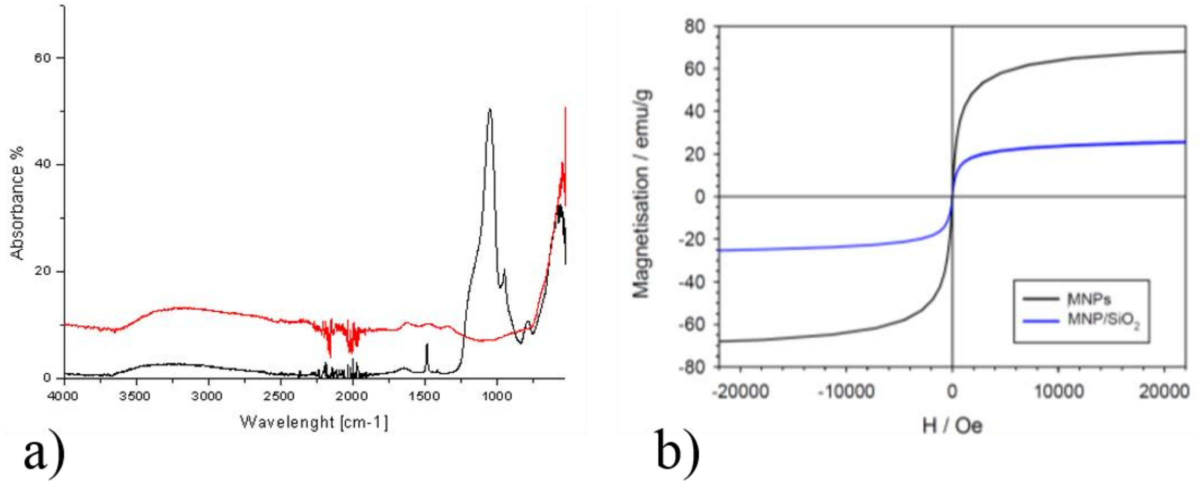

Figure S22. a) FT-IR spectroscopy: free Fe_3O_4 nanoparticles (red plot), core-shell $Fe_3O_4@SiO_2$ nanoparticles (black plot), b) Magnetization curves of Fe_3O_4 MNPs (black line) and core-shell $Fe_3O_4@SiO_2$ MNPs (blue line).

5. Dynamic Light Scattering (DLS)

DLS measurements were performed by using a Zetasizer Nano S dynamic light scattering instrument (Malvern instruments). DLA spectra were recorded at room temperature (25 ^{o}C), using a standard laser with the incident length of 633 nm and a power of 4 mW. Fe_3O_4 and $Fe_3O_4/Cd_{0.1}Zn_{0.9}Se@SiO_2$ samples were prepared in methanol and analysed in disposal semi-micro PS cuvettes (Fiscerbrand®). $Cd_{0.1}Zn_{0.9}Se$ dispersions were prepared in chloroform and analysed in a Hellma® quartz cuvette. All the particles sizes here reported are an average of diameters and relative percentage of three-time measurements (**Table S8**).

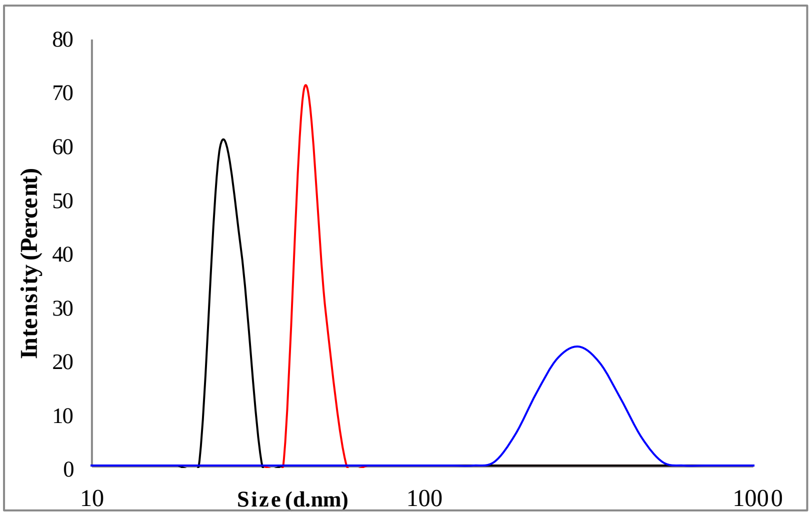

Figure S23. DLS of particles with size and perceptual distributions of Fe_3O_4 (black line), $Cd_{0.1}Zn_{0.9}Se$ (red line) and $Fe_3O_4/Cd_{0.1}Zn_{0.9}Se$ $@SiO_2$ (blue line).

Table S8. Further information on DLS-estimated particles size given as hydrodynamic diameters (nm). The estimated hydrodynamic radii in aqueous solutions appear consistently larger than those evaluated by TEM imaging (< 50 nm for $Fe_3O_4/Cd_{0.1}Zn_{0.9}Se@SiO_2$), as the presence of the stabilising polymer polyoxyethylene(5)isooctylphenyl ether at the oil-water interface cannot be discounted.

	Fe_3O_4	$Cd_{(0.1)}Zn_{(0.9)}Se$	$Fe_3O_4/Cd_{0.1}Zn_{0.9}Se@SiO_2$
1st measurement	25.9 nm	45.8 nm	303.8 nm (317.0 nm)
2nd measurement	36.7nm	144.6 nm	301.6 nm (372.4 nm)
3rd measurement	32.7 nm	76.7 nm	318.0 nm (330.0 nm)
Average size (d. nm)	31.8 nm	89.0 nm	307.8 nm (339.8 nm)

6. UV-Visible Spectroscopy

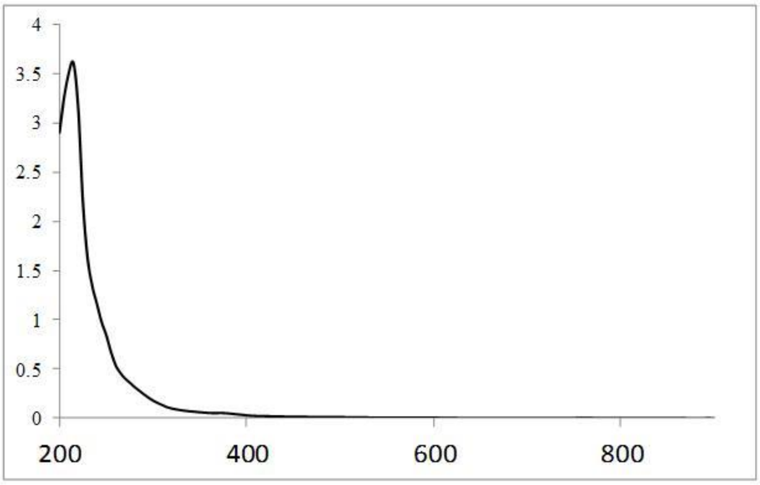

Figure S24. UV/Vis spectrum of as-made $Cd_{0.1}Zn_{0.9}Se$ quantum dots before encapsulation, (λ_{max} = 215 nm, 1 mg/ml dispersions in distilled water).

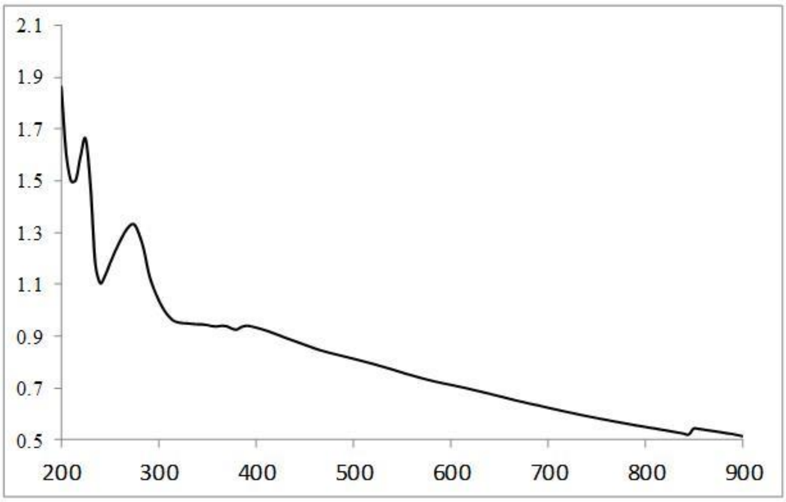

Figure S25. UV/Vis spectrum of $Fe_3O_4/Cd_{0.1}Zn_{0.9}Se@SiO_2$ (1 mg/ml dispersions in distilled water), i.e. representing absorption of $Cd_{0.1}Zn_{0.9}Se$ quantum dots when encapsulated within the NPs/silica matrix (λ_{max} = 275 nm, 225 nm and 200 nm).

7. Fluorescence Spectroscopy

The fluorescence studies were carried out using solutions at concentrations of 100 μM in HPLC-grade DMSO.

2D contour plots Compounds **2-5** were taken of in order to determine the excitation wavelength that produces the most intense fluorescence emission are shown in **Figures S26–S29**, where excitation wavelength was plotted against emission wavelength. Compound **2** was included as a way of comparing the variation in fluorescence between the free bis(thiosemicarbazone) ligand and the chelated metal complexes.

The intensity of the fluorescence for Compound **4** and **5** is greater with respect to Compound **2**, whereas the fluorescence intensity for Compound **2** has decreased. Therefore these results show chelation of zinc to the bis(thiosemicarbazone) causes a decrease in fluorescence, whereas the chelation of gallium and indium causes an increase in chelation. Overall, however, the fluorescence intensity for all the Compounds **2-5** is rather weak.

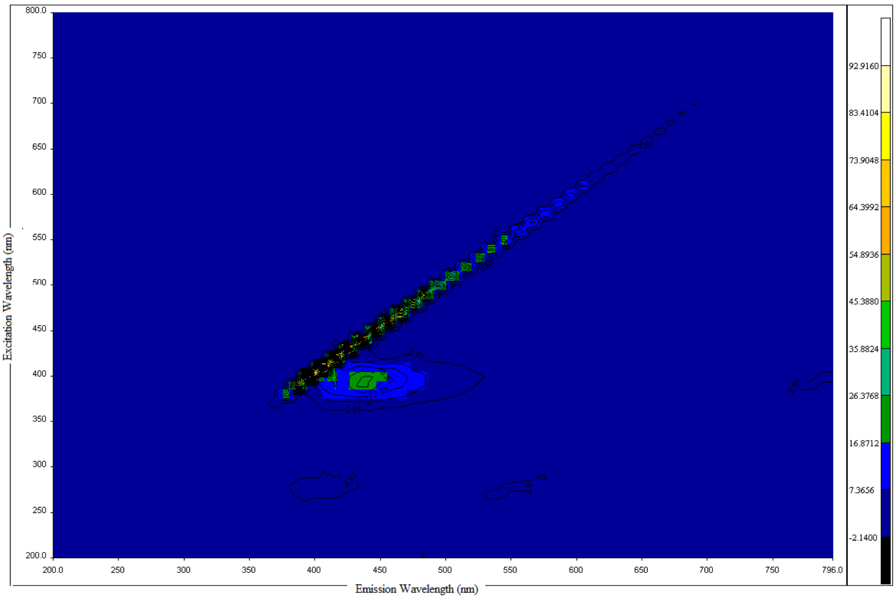

Figure S26. 2D Fluorescence plot of Compound **2**.

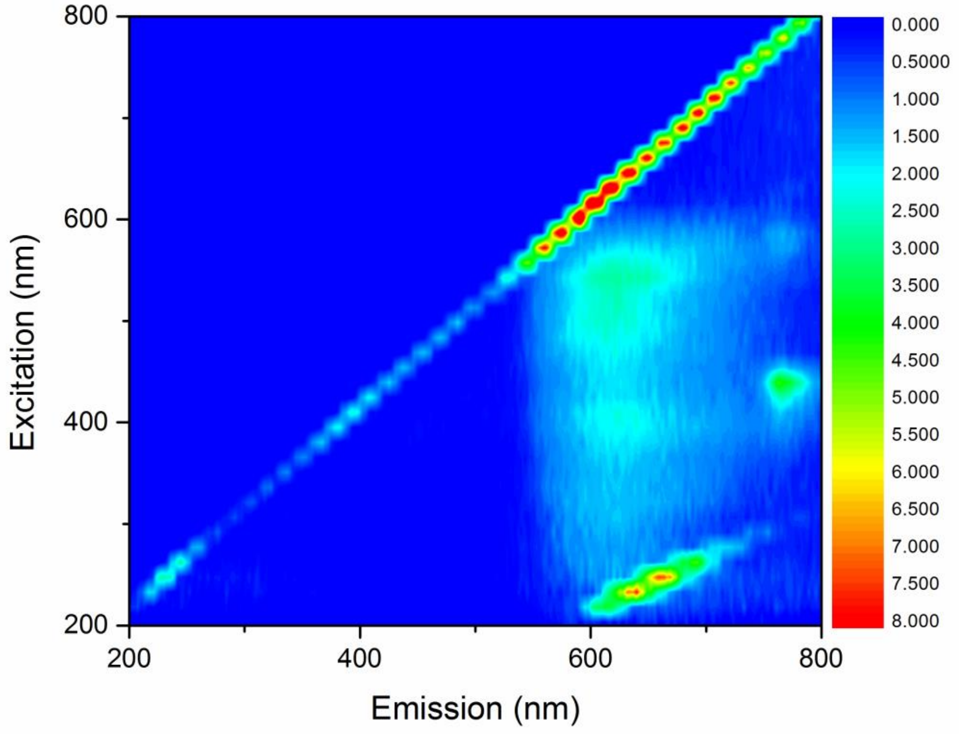

Figure S27. 2D Fluorescence plot of Compound **3**.

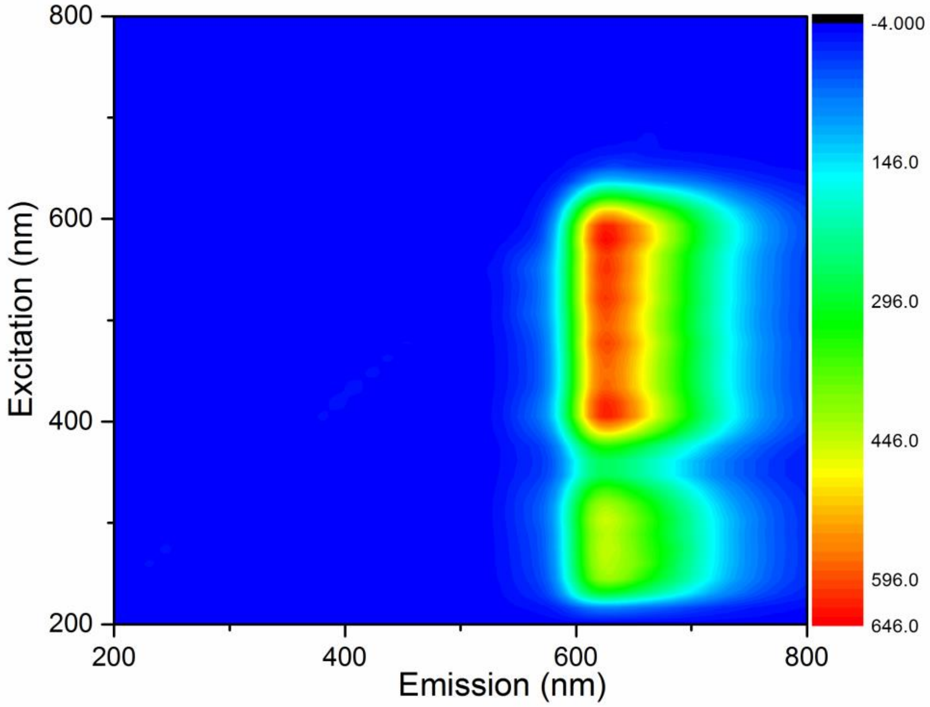

Figure S28. 2D Fluorescence plot of Compound **4**.

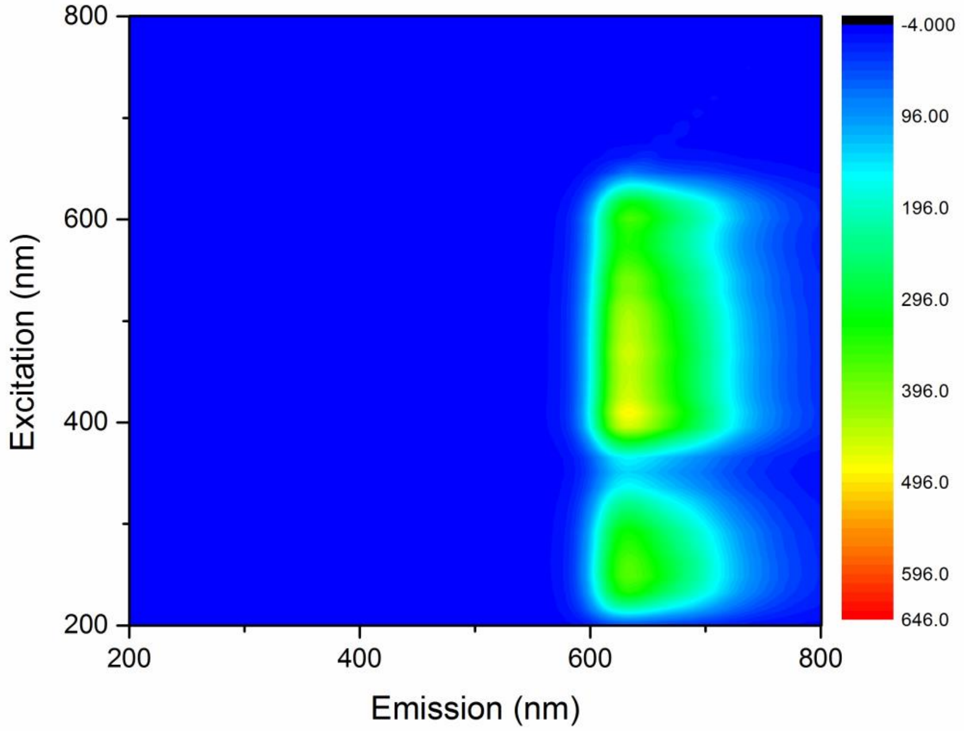

Figure S29. 2D Fluorescence plot of Compound **5**.

The plots show that each of the Compounds **2-5** has fluorescent properties that vary slightly in terms of intensity and emission wavelength, although all the compounds seem to produce the most intense fluorescence when excited with light of wavelength 390 nm.

When designing fluorescent probes it is important to ensure that the fluorescence emission of the probe is different to the excitation wavelength of any biological autofluorescence within the cell. It has generally been found that cells contain molecules that become fluorescent when excited with light in the UV/Vis region. Therefore fluorescent emission up to 500 nm is preferable. **Table S9** shows that the most intense fluorescent emissions for Compounds **2-5** all fall in the region of 500 nm, making the compounds suitable for use as fluorescent probes.

Table S9. Excitation wavelength required for the most intense fluorescence emission at the wavelength specified.

	Excitation (nm)	Emission (nm)
**2**	390	450
**3**	390	550
**4**	390	450
**5**	390	440

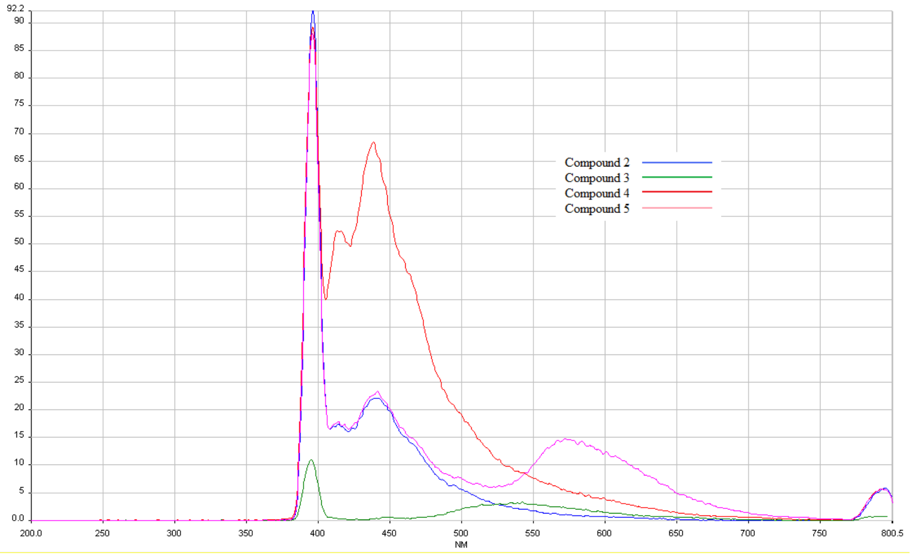

Figure S30. 1D Fluorescence plot of Compounds **2-5** for excitation at 390 nm.

It is clear from **Figure S30** that Compound **4** has the most intense fluorescence when excited with light of wavelength 400 nm. There is little difference between the fluorescence intensities of Compounds **2** and **4**, and it is again clearly shown that Compound **3** produces the weakest fluorescence.

8. Cellular Imaging and MTT assays

Cells were cultured at 37 °C in a humidified atmosphere in the air and diluted once confluence had been reached. Culture occurred in Eagle's Minimum Essential Medium (EMEM) containing 15 % foetal calf serum (FCS), 0.5 % penicillin/streptomycin and 1 % L-Glutamine. The surplus supernatant containing dead cell matter and excess protein were aspirated. The live adherent cells were then washed with 2×10 mL aliquots of phosphate buffer saline (PBS) solution to remove any remaining media containing FCS, which inactivates trypsin. Cells were re-suspended in solution by incubation in 3 mL of trypsin–PBS solution (0.25 % trypsin) for 5 min at 37 °C. After trypsinisation, 5 mL of medium containing serum was added to inactivate the trypsin and the solution was centrifuged for 5 min (1000 rpm, 25 °C) to remove any remaining dead cell matter. The supernatant liquid was aspirated and 5 mL of medium was added to the cell matter left behind. Cells were counted using a haemocytometer and then seeded as 0.15 million cells in absence of indicator dyes such as phenol red in cell medium (15 % FCS), for 48 h in poly-D-lysine coated dishes. Cells were washed in serum-free medium three times prior to addition of $Fe_3O_4/Cd_{0.1}Zn_{0.9}Se@SiO_2$ NPs, and excited using a wavelength of 460-500 nm, on a Nikon Eclipse TE2000 Epi-fluorescence microscope and a Zeiss LSM510META laser scanning confocal microscope. For each experiment images of cells were taken prior to addition, which indicate that cells were healthy, suitable for nanoparticle addition, given this initial perfectly undamaged outer cellular membranes and low background fluorescence.

In vitro fluorescence imaging of Zn[ATSM]/A

Figure S31 shows the fluorescence image of Zn[ATSM]/A in cells, a brightfield image and then an overlay of the two. The fluorescence image indicates where the compound has accumulated within the cell sample. The brightfield image captures the physical health of the cells. The overlay image allows Zn[ATSM]/A to be viewed within the cell mixture. The images show that after two hours the compound had been taken up by the cytoplasm of the cell, as seen in **Figure S31**. There was no precipitation and it appeared that Zn[ATSM]/A had accumulated in liposomes as seen by the formation of physically organised luminous spots. It can be seen in the brightfield image in **Figure S32** that there has been some cell death meaning that the compound is toxic to the cells after 72 h exposure.

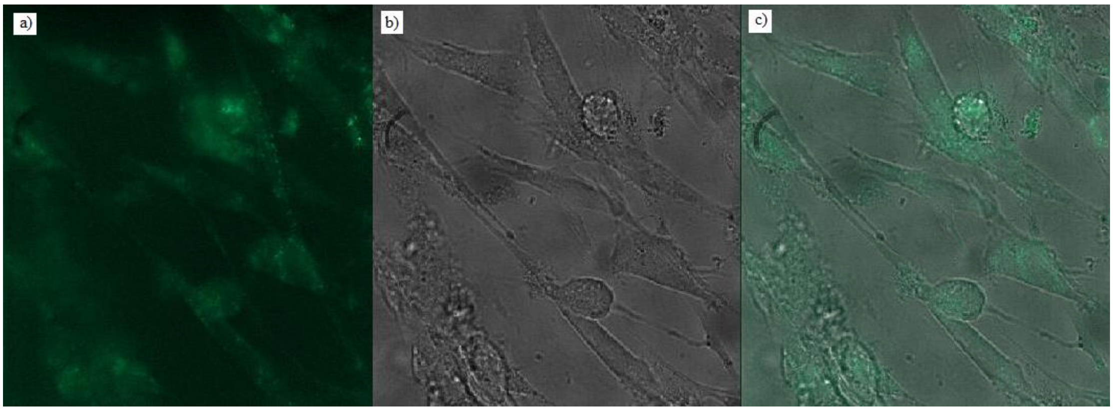

Figure S31 a) Epifluorescence emission (green), b) brightfield and c) overlay images of the fluorescence of Zn[ATSM]/A in cells after 2 hours. Scale bar: 20 µm.

Further images were taken 72 hours after Zn[ATSM]/A had been introduced to the cells, as seen in **Figure S32**. The false colour fluorescent image shows that over time there is a stronger uptake of this complex in the cells. It can be seen that the compound has now accumulated in the nucleus as well as the cytoplasm. The bright field image still shows the cells to look fairly healthy, although again, around the edges of the image, it appears to be some distortion of the cells suggesting cell death.

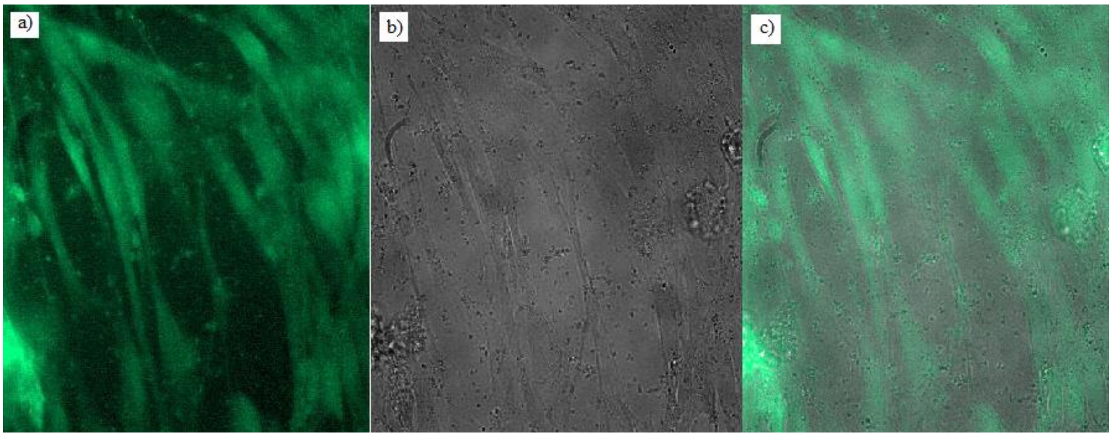

Figure S32. a) Epi-fluorescence emission (green), b) brightfield and c) overlay images of the fluorescence of Zn[ATSM]/A in cells after 72 h, 37 °C. Scale bar: 20 µm.

In vitro fluorescence imaging of $Fe_3O_4/Cd_{0.1}Zn_{0.9}Se@SiO_2$

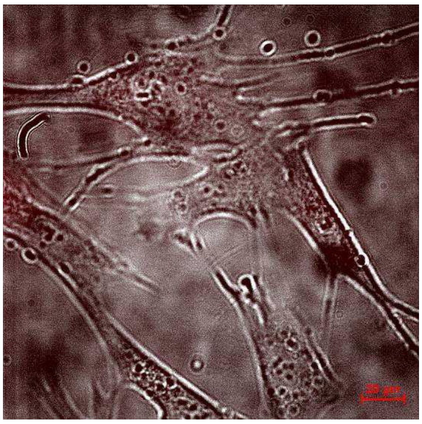

Figure S33. Epifluorescence micrograph for a control experiment showing living cells of FEK-4. Scale bar: 20 μm.

Figures 34 and 35 show epifluorescence micrograms of FEK-4 cells incubated 30 minutes with high and low concentrations $Fe_3O_4/Cd_{0.1}Zn_{0.9}Se@SiO_2$ (**C**) respectively. Fields of view were recorded before washing the cells with PBS. Although the particles were homogeneously distributed within the plate, no cellular uptake was observed. Thus, the incubation time was prolonged to one hour. However, after 60 minutes, no internalization or membrane accumulation was seen. These experiments suggest that $Fe_3O_4/Cd_{0.1}Zn_{0.9}Se@SiO_2$ (**C**) slowly cross the membrane of FEK-4 cells and do not alter their morphology, suggesting a low level of cytotoxicity within the timescale considered.

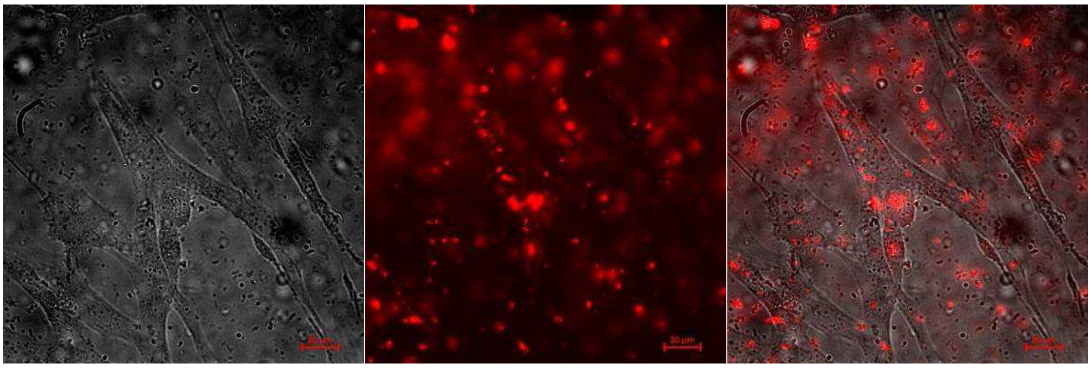

a) **b)** **c)**

Figure S34. Epifluorescence micrograph of $Fe_3O_4/Cd_{0.1}Zn_{0.9}Se@SiO_2$ incubated for 30 minutes, recorded prior to PBS washing, where a) is bright-field image, b) fluorescence channel, λ_{ex} = 460-500 nm and c) is an overlay of (a) and (b). Scale bar: 20 μm.

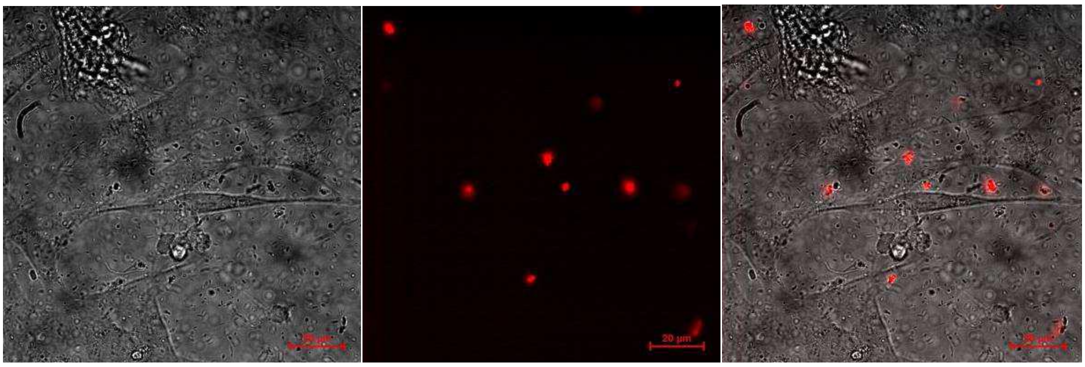

a) b) c)

Figure S35. Epifluorescence micrograph of $Fe_3O_4/Cd_{0.1}Zn_{0.9}Se@SiO_2$ incubated for 30 minutes, recorded prior to PBS washing, where a) is bright-field image, b) fluorescence channel, λ_{ex} = 460-500 nm and c) is an overlay of (a) and (b). Scale bar: 20 µm.

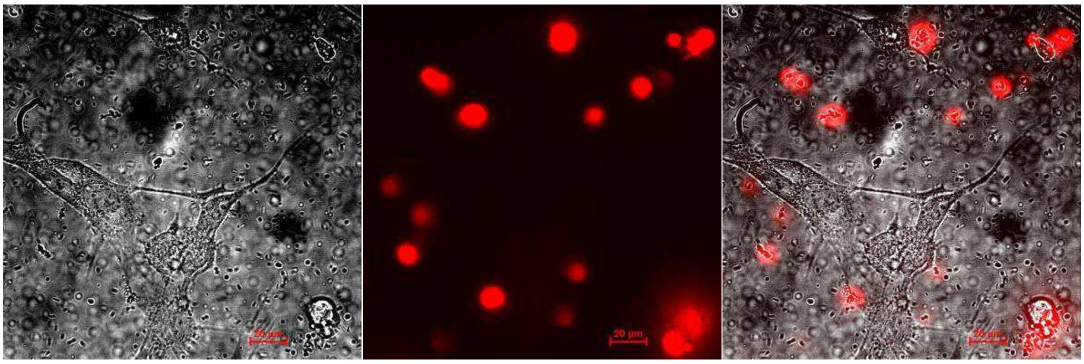

a) b) c)

Figure S36. Epifluorescence micrograph of $Fe_3O_4/Cd_{0.1}Zn_{0.9}Se@SiO_2$ incubated in FEK-4 cells for 60 minutes, recorded prior to PBS washing, where a) is bright-field image, b) fluorescence channel, λ_{ex} = 460-500 nm and c) is an overlay of (a) and (b). Scale bar: 20 µm.

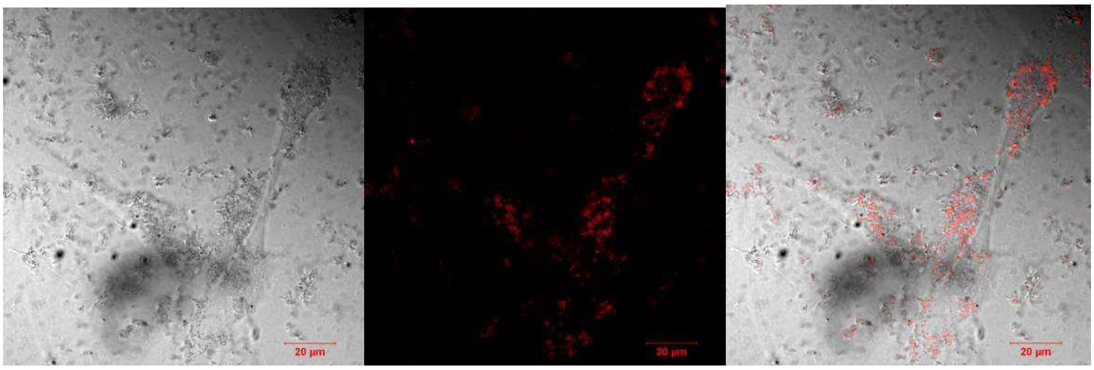

a) b) c)

Figure S37. Confocal micrograph of $Fe_3O_4/Cd_{0.1}Zn_{0.9}Se@SiO_2$ incubated in FEK-4 cells for 4 hours, washed twice with PBS and returned to serum-free medium, where a) is DIC image, b) fluorescence channel, λ_{ex} = 488 nm and c) is an overlay of (a) and (b). Scale bar: 20 µm.

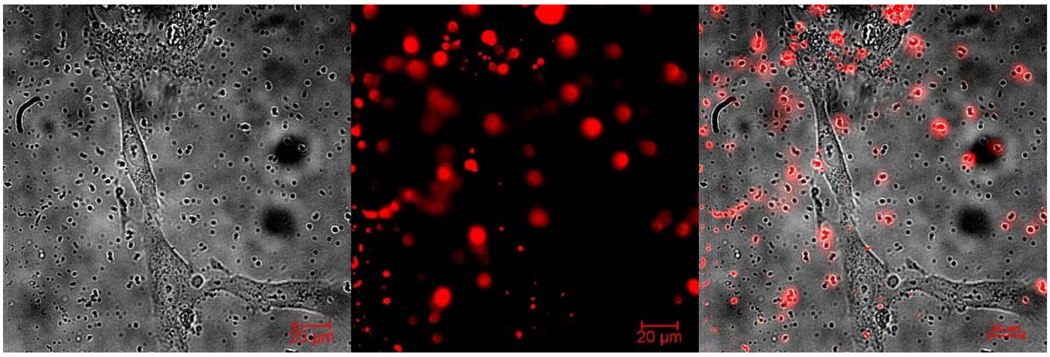

a) **b)** **c)**

Figure S38. Epifluorescence micrograph of $Fe_3O_4/Cd_{0.1}Zn_{0.9}Se@SiO_2$ incubated in FEK-4 cells for 6 hours, where a) is bright-field image, b) fluorescence channel, λ_{ex} = 460-500 nm and c) is an overlay of (a) and (b). Scale bar: 20 µm.

Confocal Imaging

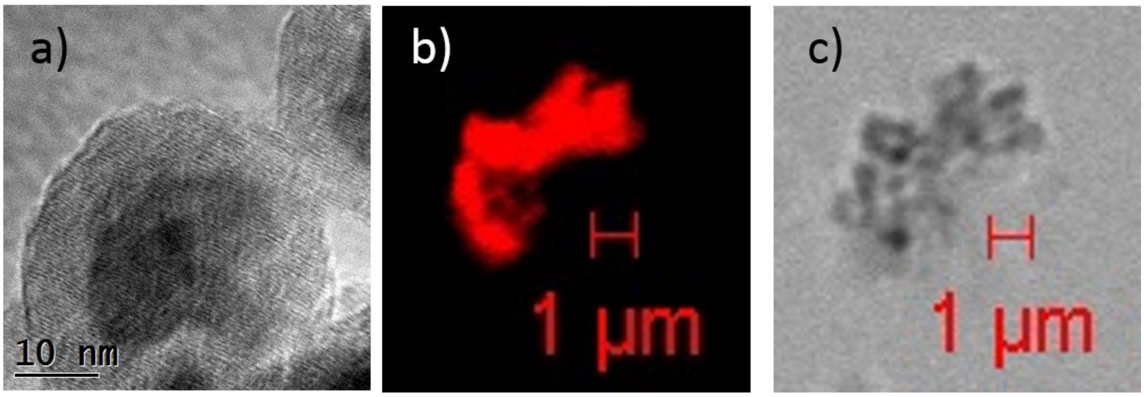

Figure S39. a) HRTEM micrograph of $Fe_3O_4/Cd_{0.1}Zn_{0.9}Se@SiO_2$, b) representative fluorescence image of $Fe_3O_4/Cd_{0.1}Zn_{0.9}Se@SiO_2$ in aqueous media, λ_{ex} = 488 nm, and c) its differential interference contrast (DIC) channel.

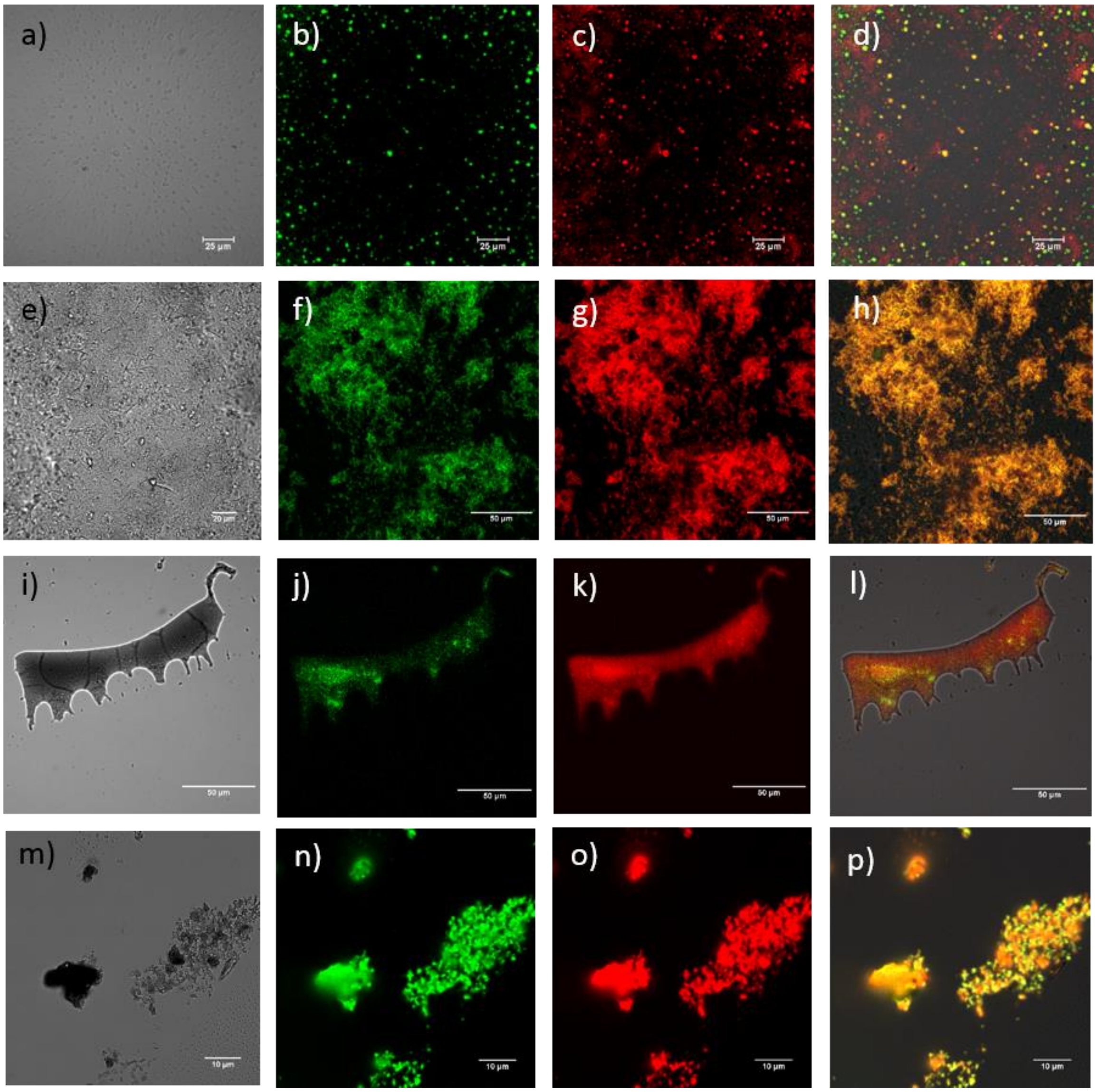

Figure S40. Single-photon laser-scanning confocal microscopy of thin films drying out on a horizontal surface of a-d) CdSe, λ_{ex} = 488 nm; e-h) $Cd_{0.1}Zn_{0.9}Se$, λ_{ex} = 488 nm; i-l) $Fe_3O_4/Cd_{0.1}Zn_{0.9}Se@SiO_2$, λ_{ex} = 488 nm; m-p) $Fe_3O_4@SiO_2@Zn$[ATSM]/A; λ_{ex} = 488 nm; a, e, i, m) DIC channel; b, f, j, n) green channel (λ_{em} = 500-550 nm); c, g, k, o) red channel (λ_{em} = 570-750 nm); d, h, l, p) overlay of the DIC-green-red channels. Scale bar: a-d) 25 μm; e-l) 50 μm; m-p) 10 μm.

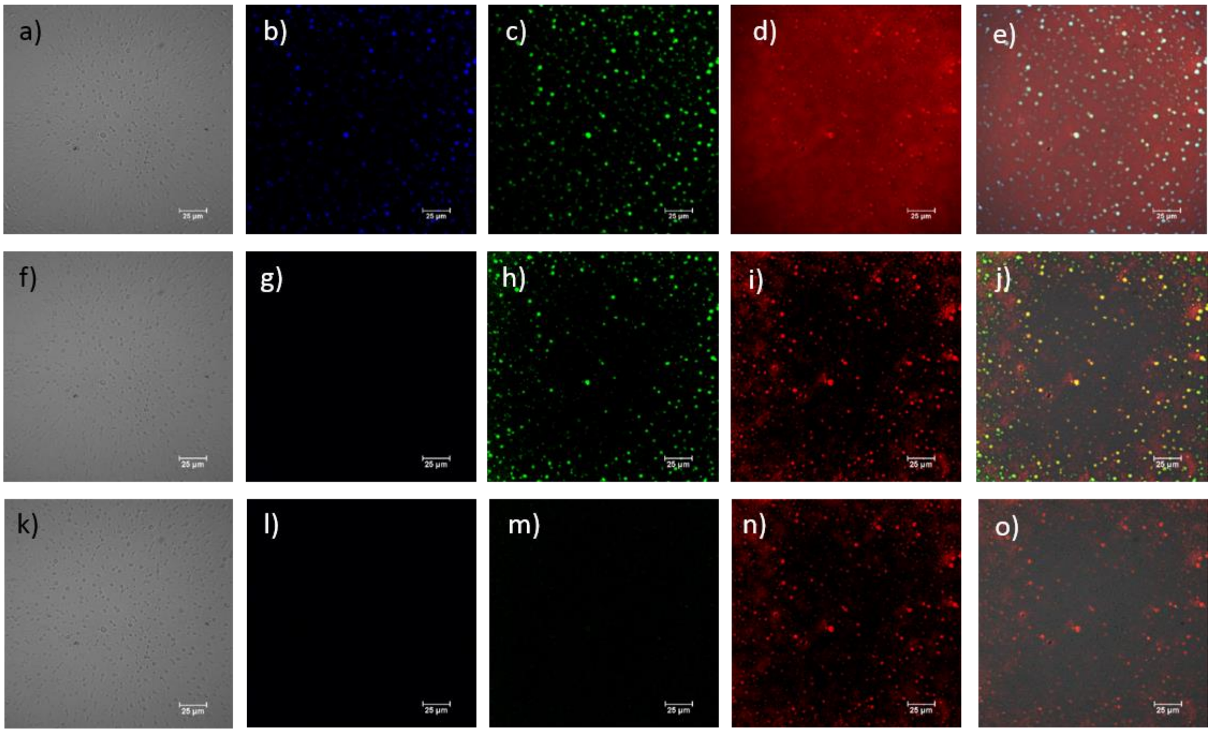

Figure S41. Complete overview of single-photon laser-scanning confocal microscopy of CdSe thin film of a chloroform suspension (0.1 mmol in 3 mL of $CHCl_3$) drying out on a horizontal surface using 3 different lasers. a-e) λ_{ex} = 405.0 nm; f-j) λ_{ex} = 488.0 nm; k-o) λ_{ex} = 561.0 nm. a, f, k) DIC channel; b, g, l) blue channel (λ_{em} = 417-477 nm); c, h, m) green channel (λ_{em} = 500-550 nm); d, i, n) red channel (λ_{em} = 570-750 nm). e, j, o) overlay of the blue-green-red channels. Scale bar: 25 μm.

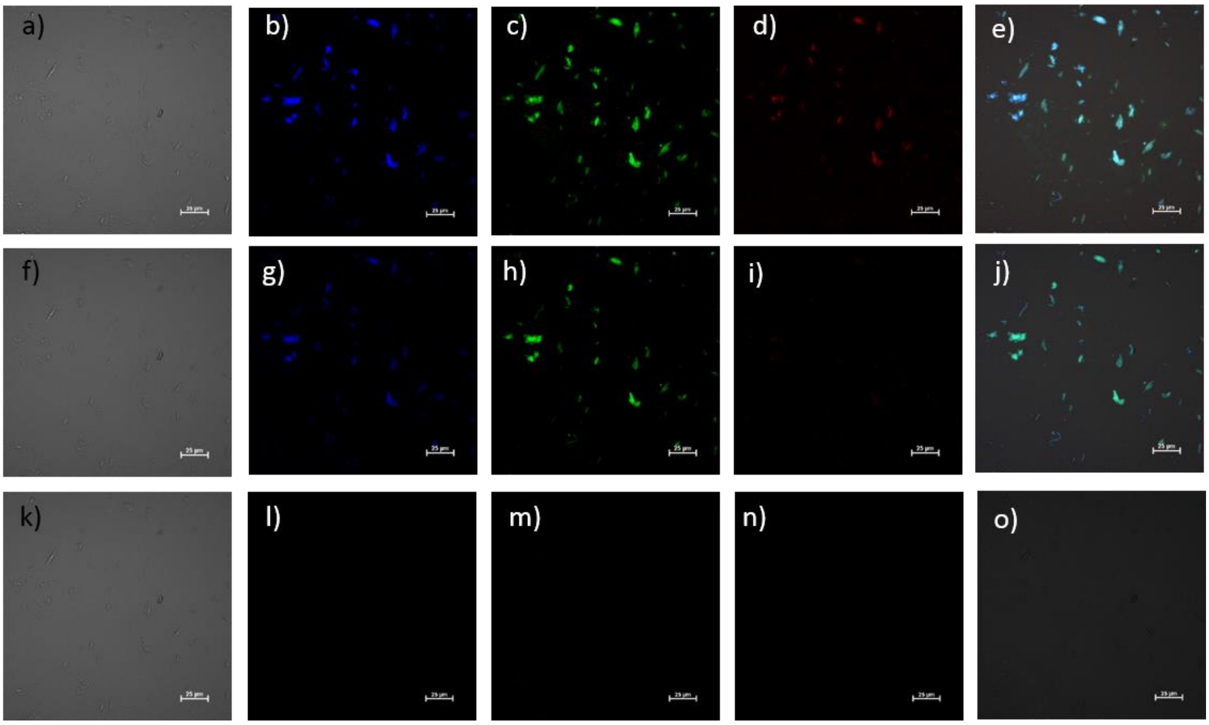

Figure S42. Complete overview of single-photon laser-scanning confocal microscopy of Lumidots^{TM} CdSe/ZnS 480 nm thin film of a toluene suspension drying out on a horizontal surface using 3 different lasers. a-e) λ_{ex} = 405.0 nm; f-j) λ_{ex} = 488.0 nm; k-o) λ_{ex} = 561.0 nm. a, f, k) DIC channel; b, g, l) blue channel (λ_{em} = 417-477 nm); c, h, m) green channel (λ_{em} = 500-550 nm); d, i, n) red channel (λ_{em} = 570-750 nm). e, j, o) overlay of the blue-green-red channels. Scale bar: 25 µm.

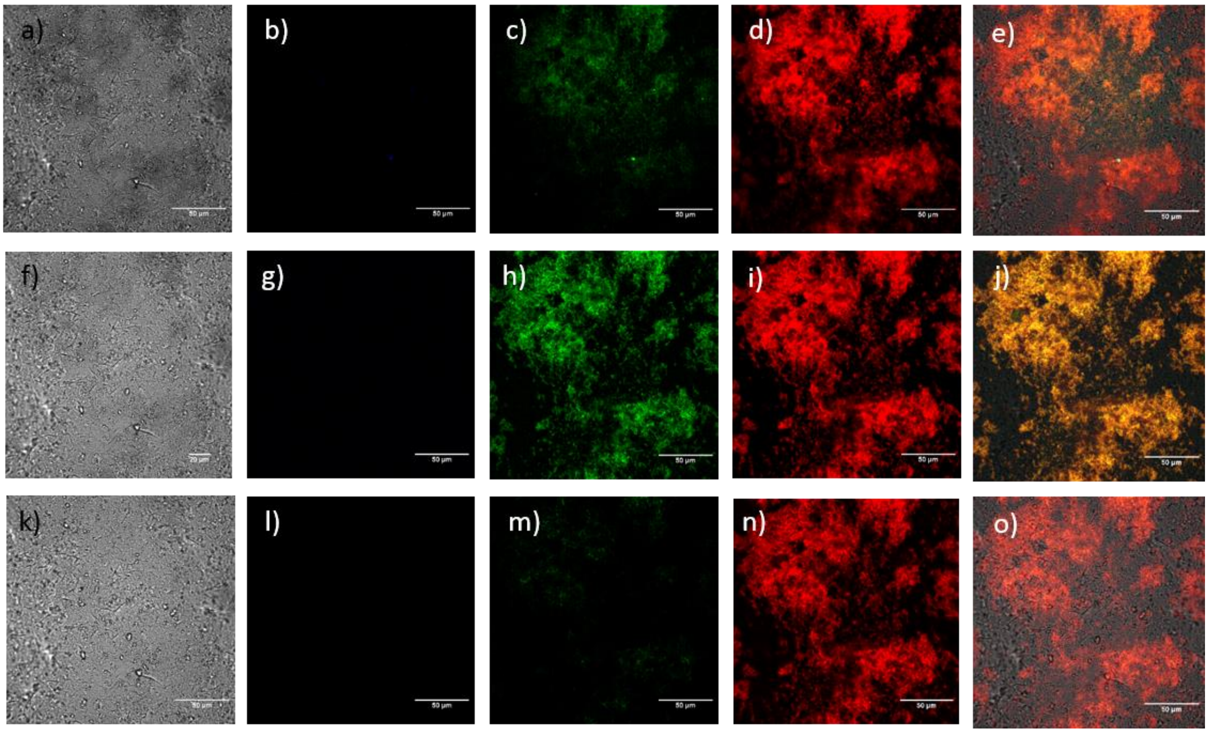

Figure S43. Complete overview of single-photon laser-scanning confocal microscopy of $Cd_{0.1}Zn_{0.9}Se$ thin film of a methanol suspension drying out on a horizontal surface using 3 different lasers. a-e) λ_{ex} = 405.0 nm; f-j) λ_{ex} = 488.0 nm; k-o) λ_{ex} = 561.0 nm. a, f, k) DIC channel; b, g, l) blue channel (λ_{em} = 417-477 nm); c, h, m) green channel (λ_{em} = 500-550 nm); d, i, n) red channel (λ_{em} = 570-750 nm). e, j, o) overlay of the blue-green-red channels. Scale bar: 50 μm.

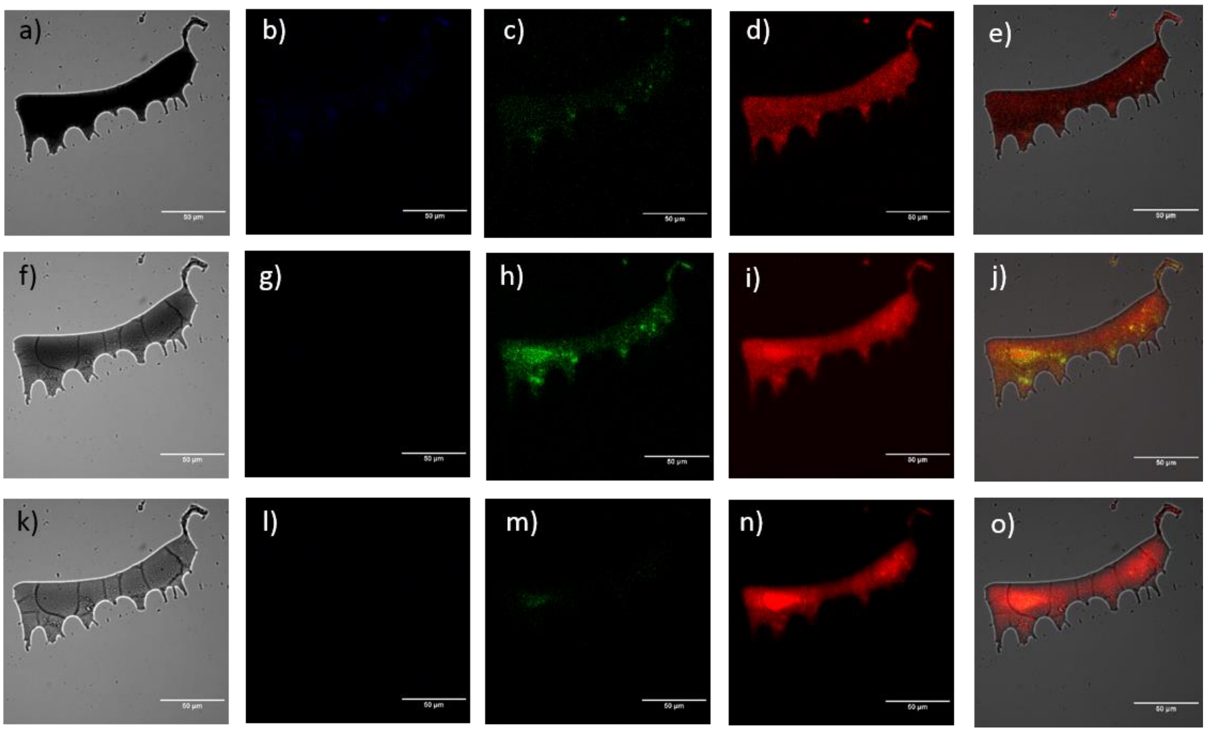

Figure S44. Complete overview of single-photon laser-scanning confocal microscopy of $Fe_3O_4/Cd_{0.1}Zn_{0.9}Se@SiO_2$ thin film of a methanol suspension drying out on a horizontal surface using 3 different lasers. a-e) λ_{ex} = 405.0 nm; f-j) λ_{ex} = 488.0 nm; k-o) λ_{ex} = 561.0 nm. a, f, k) DIC channel; b, g, l) blue channel (λ_{em} = 417-477 nm); c, h, m) green channel (λ_{em} = 500-550 nm); d, i, n) red channel (λ_{em} = 570-750 nm). e, j, o) overlay of the blue-green-red channels. Scale bar: 50 μm.

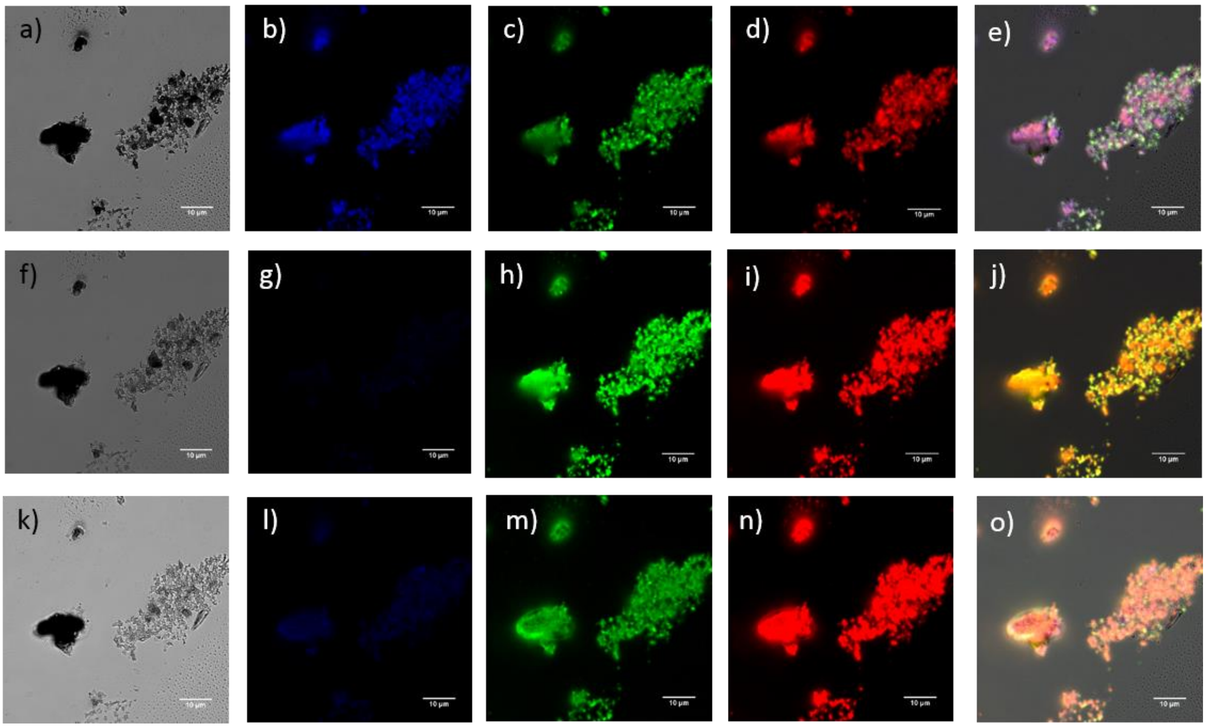

Figure S45. Complete overview of single-photon laser-scanning confocal microscopy of Fe_3O_4@SiO_2@Zn[ATSM]/A thin film of a methanol suspension drying out on a horizontal surface using 3 different lasers. a-e) λ_{ex} = 405.0 nm; f-j) λ_{ex} = 488.0 nm; k-o) λ_{ex} = 561.0 nm. a, f, k) DIC channel; b, g, l) blue channel (λ_{em} = 417-477 nm); c, h, m) green channel (λ_{em} = 500-550 nm); d, i, n) red channel (λ_{em} = 570-750 nm). e, j, o) overlay of the blue-green-red channels. Scale bar: 10 μm.

9. Further *in vitro* epifluorescence and confocal imaging

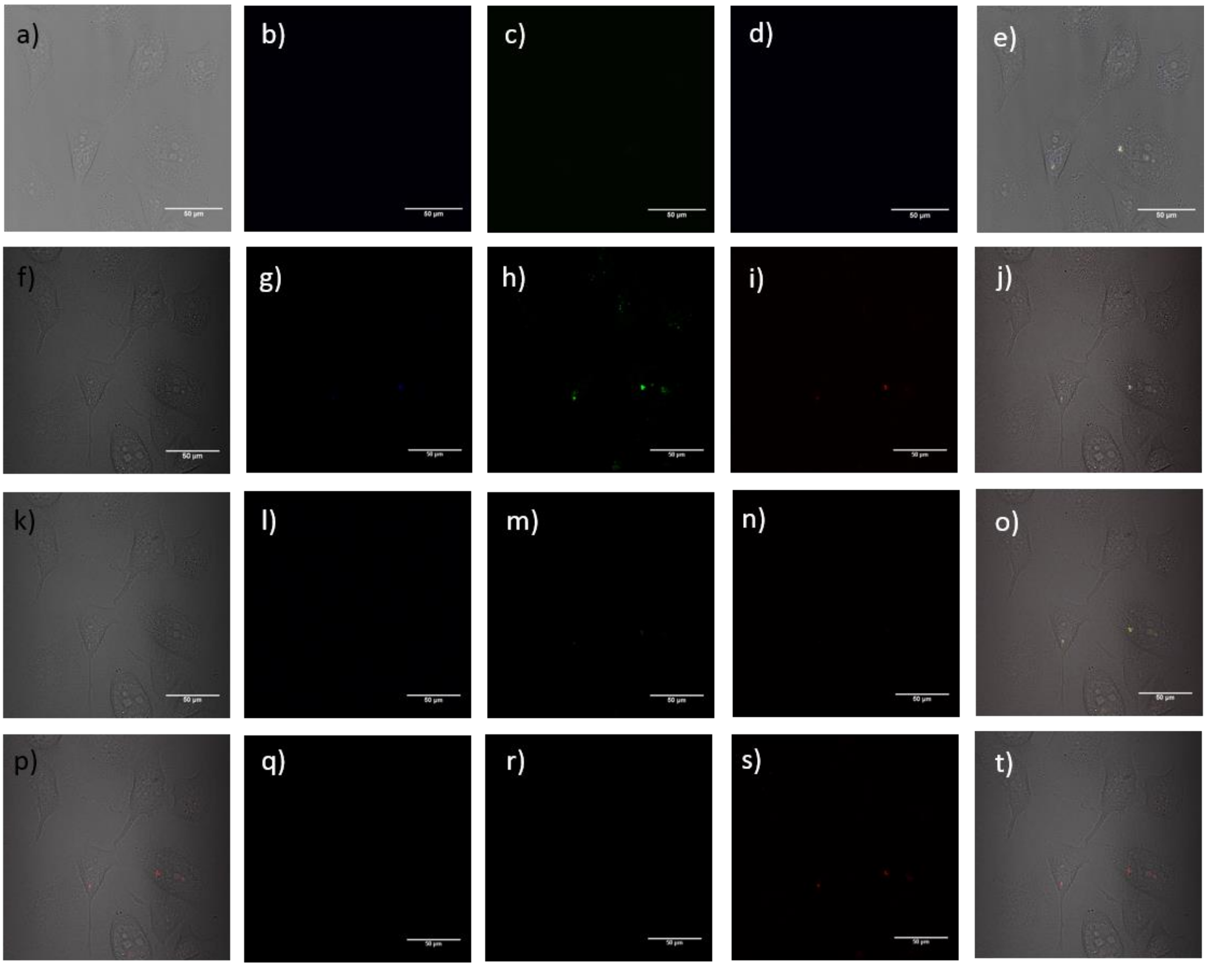

Figure S46. a-e) Epi-fluorescence adquisition and f-t) single-photon laser-scanning confocal microscopy of PC-3 cells incubated for 15 min 37 °C. f-j) λ_{ex} = 405.0 nm; k-o) λ_{ex} = 488.0 nm; p-t) λ_{ex} = 561.0 nm a, f, k, p) DIC channel; b, g, l, q) blue channel (λ_{em} = 417-477 nm); c, h, m, r) green channel (λ_{em} = 500-550 nm); d, i, n, s) red channel (λ_{em} = 570-750 nm). e, j, o, t) overlay of the blue-green-red channels. Scale bar: 50 μm.

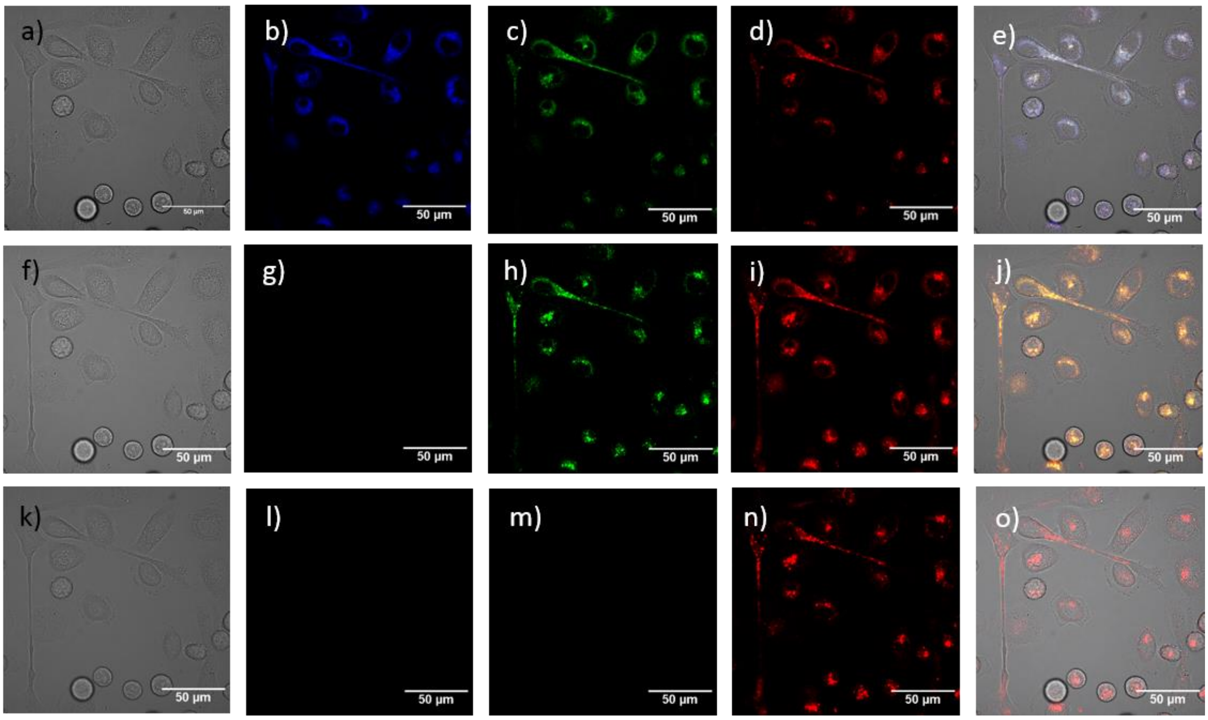

Figure S47. Single-photon laser-scanning confocal microscopy of PC-3 cells incubated for 15 min with $Fe_3O_4/Cd_{0.1}Zn_{0.9}Se@SiO_2$. Final concentrations: 10 µg/mL in 1 : 99 DMSO : serum free medium at 37 °C. a-e) λ_{ex} = 405.0 nm; f-j) λ_{ex} = 488.0 nm; k-o) λ_{ex} = 561.0 nm. a, f, k) DIC channel; b, g, l) blue channel (λ_{em} = 417-477 nm); c, h, m) green channel (λ_{em} = 500-550 nm); d, i, n) red channel (λ_{em} = 570-750 nm). e, j, o) overlay of the blue-green-red channels. Scale bar: 50 µm.

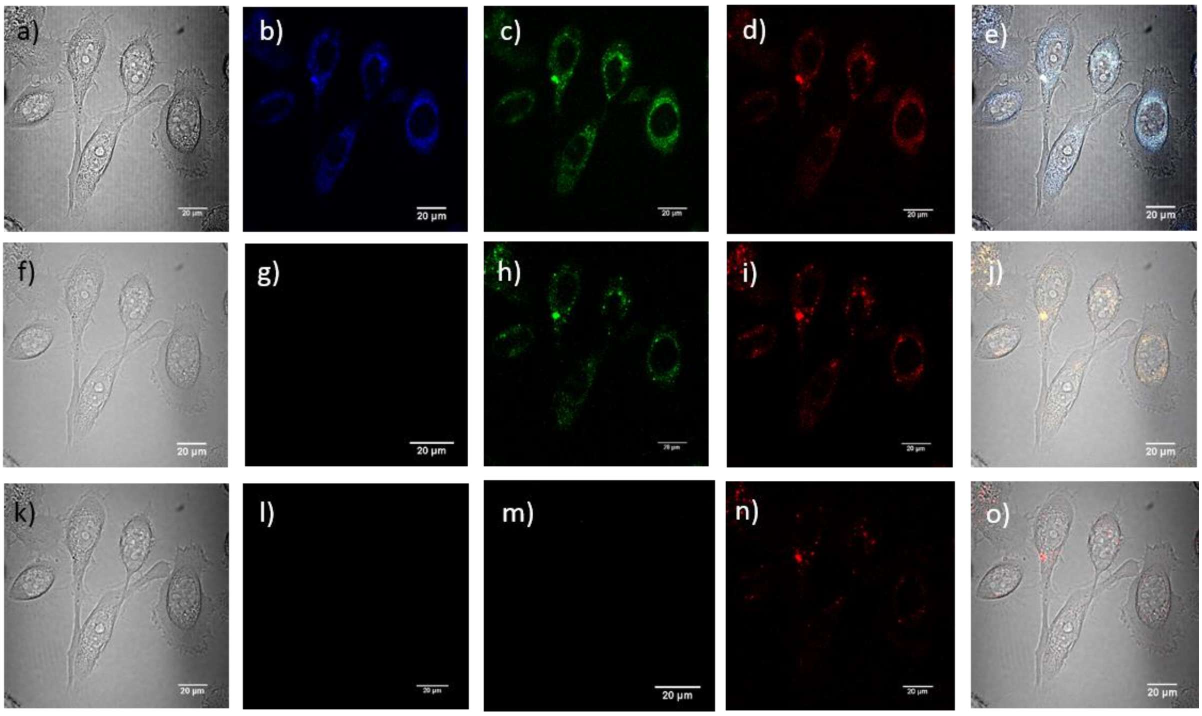

Figure S48. Single-photon laser-scanning confocal microscopy of PC-3 cells incubated for 15 min with $Fe_3O_4/Cd_{0.1}Zn_{0.9}Se@SiO_2$. Final concentrations: 10 µg/mL in 1 : 99 DMSO : serum free medium at 37 °C. a-e) λ_{ex} = 405.0 nm; f-j) λ_{ex} = 488.0 nm; k-o) λ_{ex} = 561.0 nm. a, f, k) DIC channel; b, g, l) blue channel (λ_{em} = 417-477 nm); c, h, m) green channel (λ_{em} = 500-550 nm); d, i, n) red channel (λ_{em} = 570-750 nm). e, j, o) overlay of the blue-green-red channels. Scale bar: 20 µm.

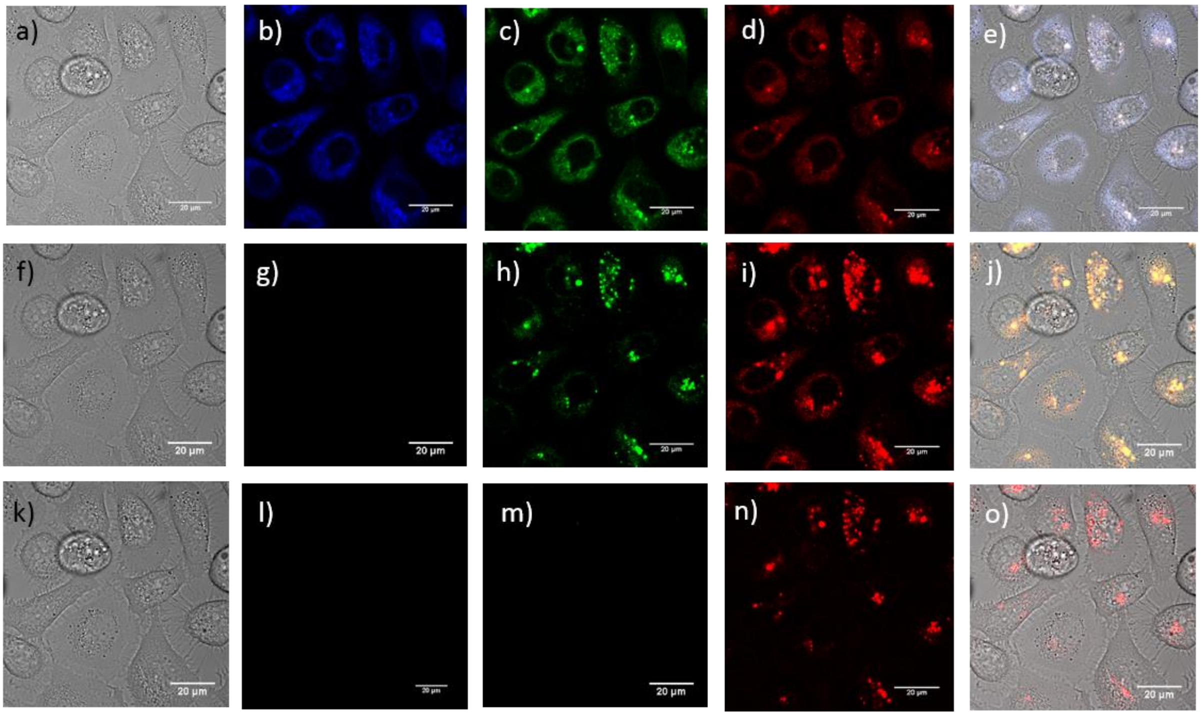

Figure S49. Single-photon laser-scanning confocal microscopy of PC-3 cells incubated for 15 min with $Fe_3O_4/Cd_{0.1}Zn_{0.9}Se@SiO_2$. Final concentration: 10 µg/mL in 1 : 99 DMSO : serum free medium at 37 °C. a-e) λ_{ex} = 405.0 nm; f-j) λ_{ex} = 488.0 nm; k-o) λ_{ex} = 561.0 nm. a, f, k) DIC channel; b, g, l) blue channel (λ_{em} = 417-477 nm); c, h, m) green channel (λ_{em} = 500-550 nm); d, i, n) red channel (λ_{em} = 570-750 nm). e, j, o) overlay of the blue-green-red channels. Scale bar: 20 µm.

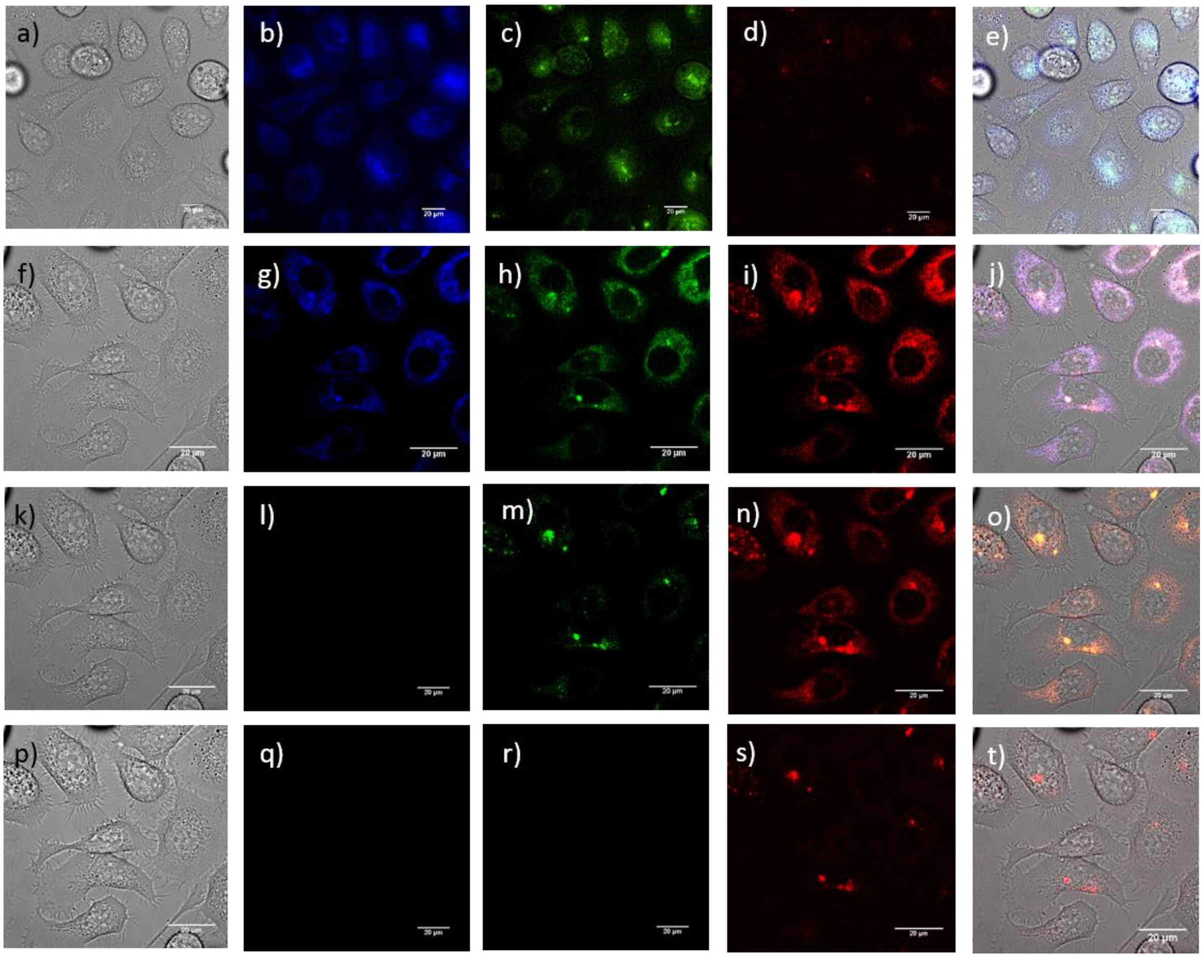

Figure S50. a-e) Epi-fluorescence adquisition and f-t) single-photon laser-scanning confocal microscopy of PC-3 cells incubated for 15 min with $Fe_3O_4/Cd_{0.1}Zn_{0.9}Se@SiO_2$; of $Fe_3O_4/Cd_{0.1}Zn_{0.9}Se@SiO_2$. Final concentration: 10 µg/mL in 1 : 99 DMSO : serum free medium at 37 °C.f-j) λ_{ex} = 405.0 nm; k-o) λ_{ex} = 488.0 nm; p-t) λ_{ex} = 561.0 nm a, f, k, p) DIC channel; b, g, l, q) blue channel (λ_{em} = 417-477 nm); c, h, m, r) green channel (λ_{em} = 500-550 nm); d, i, n, s) red channel (λ_{em} = 570-750 nm). e, j, o, t) overlay of the blue-green-red channels. Scale bar: 20 µm.

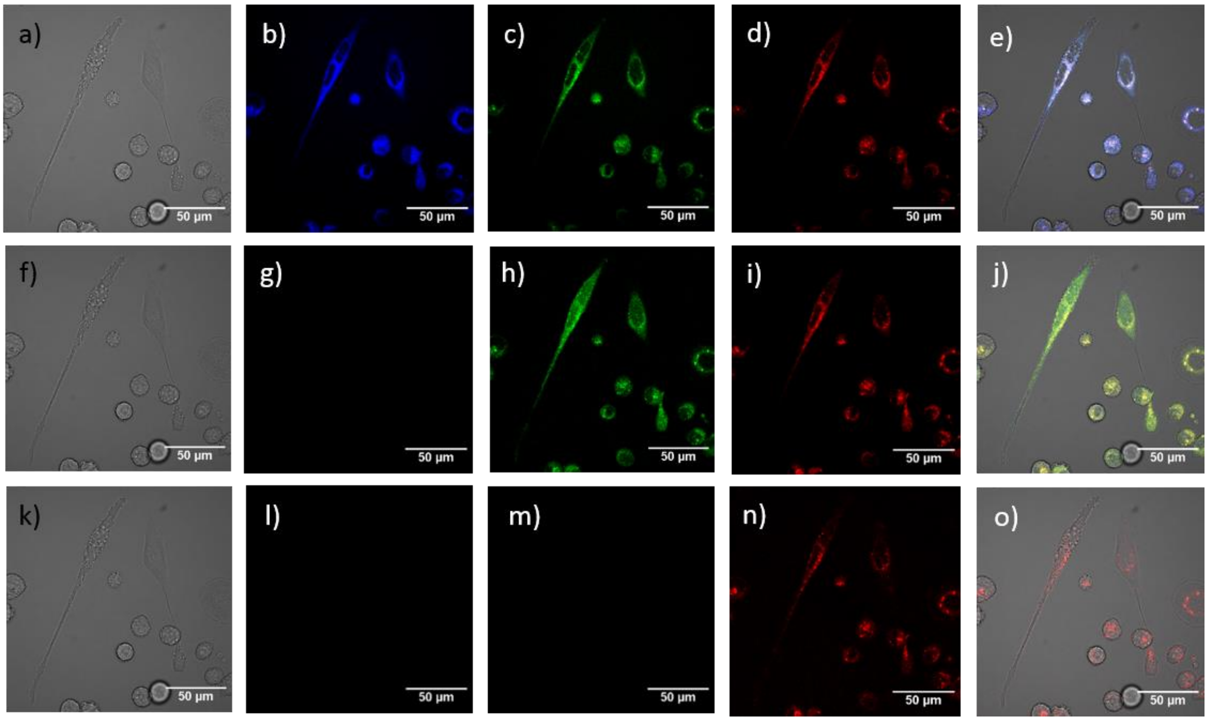

Figure S51. Single-photon laser-scanning confocal microscopy of PC-3 cells incubated for 15 min with Fe_3O_4@SiO_2@Zn[ATSM]/A NPs. Final concentration: 10 µg/mL in 1 : 99 DMSO : serum free medium at 37 °C. a, f, k) DIC channel; b, g, l) blue channel (λ_{em} = 417-477 nm); c, h, m) green channel (λ_{em} = 500-550 nm); d, i, n); red channel (λ_{em} = 570-750 nm); e, j, o) overlay of the blue-green-red channels. a-e) λ_{ex} = 405.0 nm; f-j) λ_{ex} = 488.0 nm; k-o) λ_{ex} = 561.0 nm. Scale bar: 20 µm.

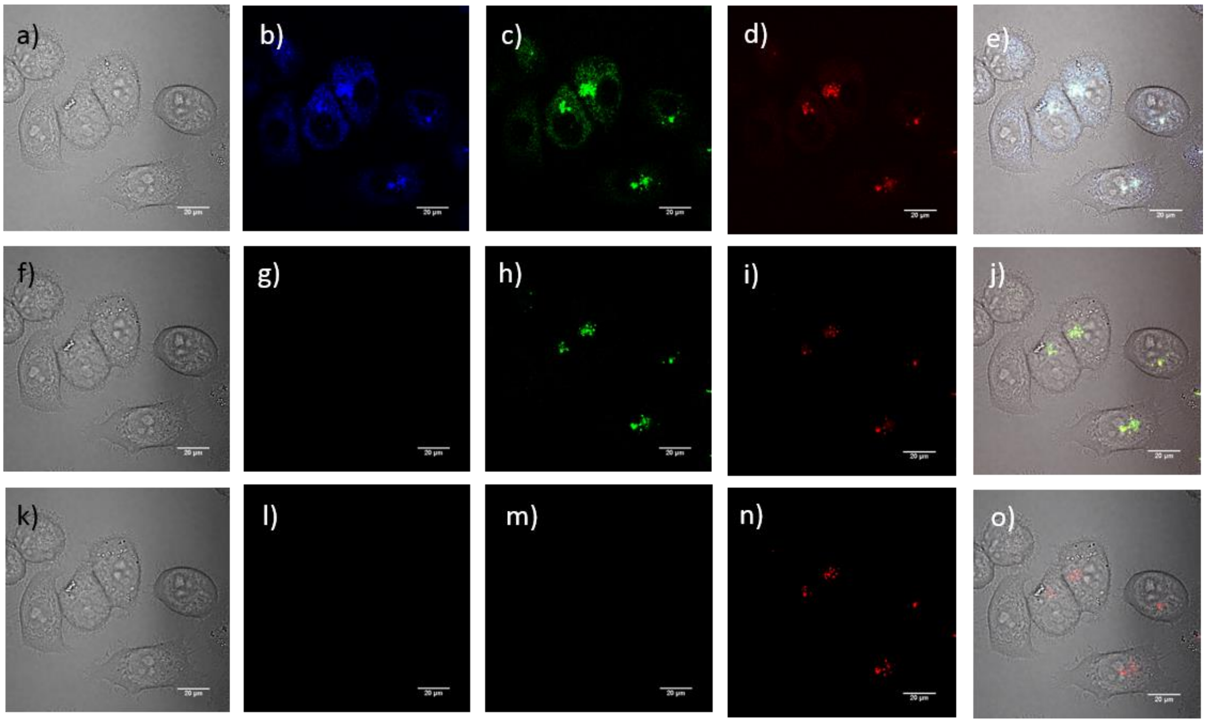

Figure S52. Single-photon laser-scanning confocal microscopy of PC-3 cells incubated for 15 min with Fe_3O_4@SiO_2@Zn[ATSM]/A NPs. Final concentration: 10 µg/mL in 1 : 99 DMSO : serum free medium at 37 °C. a, f, k) DIC channel; b, g, l) blue channel (λ_{em} = 417-477 nm); c, h, m) green channel (λ_{em} = 500-550 nm); d, i, n); red channel (λ_{em} = 570-750 nm); e, j, o) overlay of the blue-green-red channels. a-e) λ_{ex} = 405.0 nm; f-j) λ_{ex} = 488.0 nm; k-o) λ_{ex} = 561.0 nm. Scale bar: 20 µm.

10. Fluorescence-lifetime imaging microscopy (FLIM)

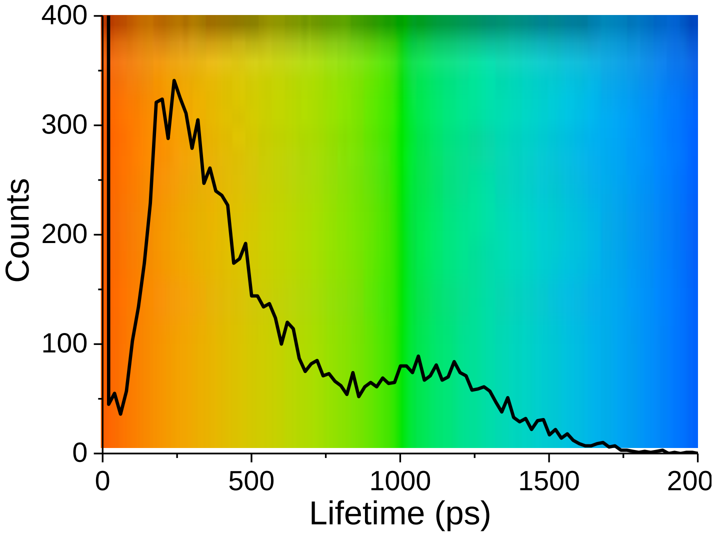

Figure S53. Fluorescence lifetime distribution curve of cells treated with 10 μL Zn[ATSM]/A with 1% DMSO in PC3 cells. λex = 810 nm, laser power was 3.7 mW.

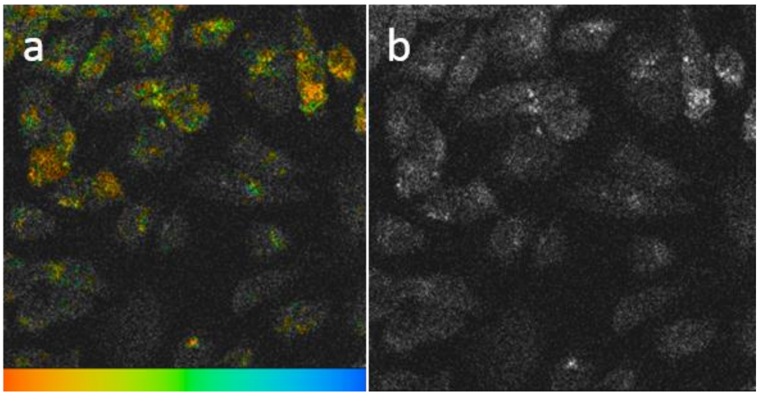

Figure S54. Fluorescence lifetime imaging microscopy (FLIM) micrographs of cells treated with 10 μL Zn[ATSM]/A with 1% DMSO, cells were incubated at 37 °C for 15 min, where **a)** the fluorescence lifetime mapping and scale bar of Zn[ATSM]/A in PC3 cells, and **b)** two photon fluorescence intensity diagram of Zn[ATSM]/A in PC3 cells.

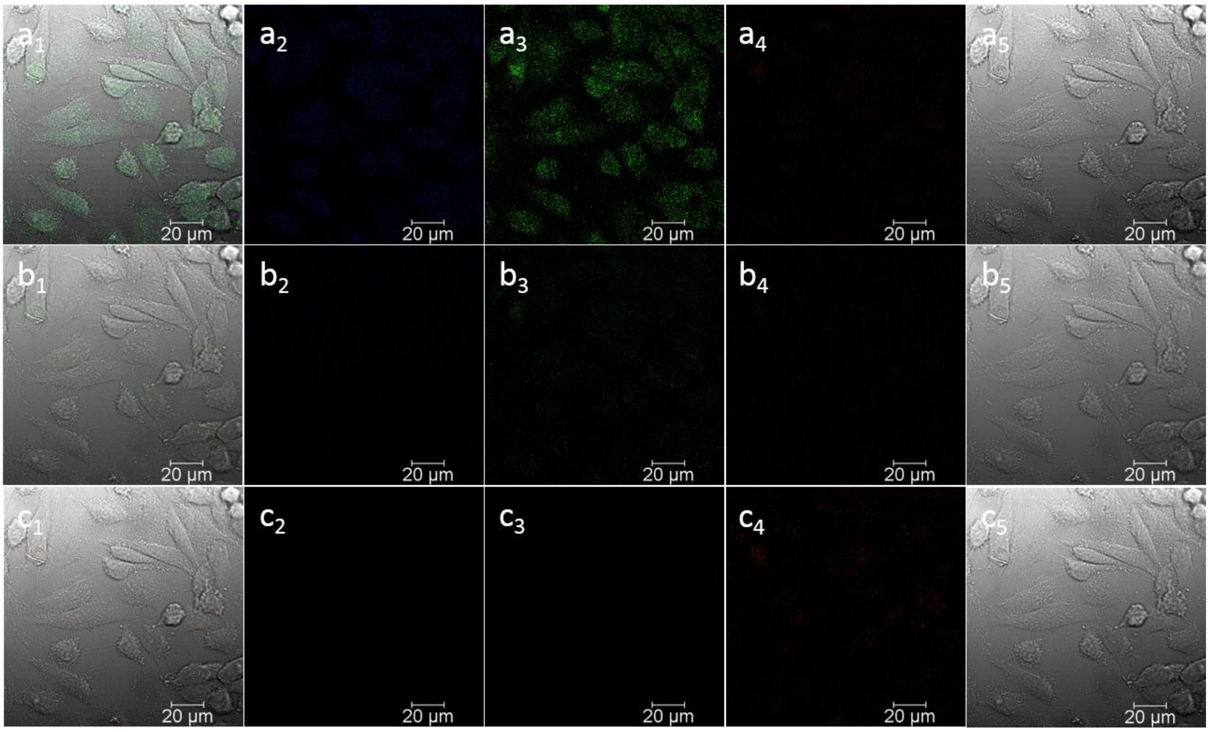

Figure S55. Two photon laser scanning confocal imaging of cancerous (PC3) cells incubated at 37 °C with 100 µl Zn[ATSM]/A in 1% DMSO (1:99% DMSO:RPMI) – 15 min incubation showing DIC-blue-green-red channel overlay (a_1,b_1 and c_1), blue channel (420 nm) (a_2, b_2 and c_2), green channel (515-530 nm) (a_3, b_3 and c_3) and red channel (605-675 nm) (a_4, b_4 and c_4) and DIC (a_5, b_5 and c_5). Scale bar: 20 µm.

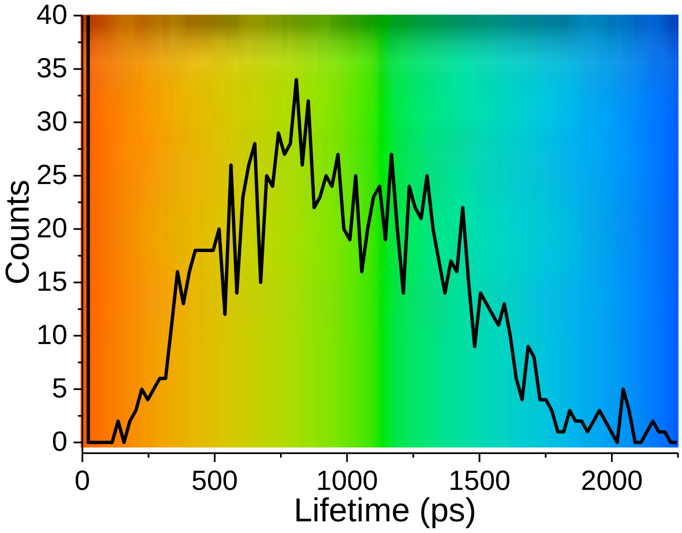

Figure S56. Fluorescence lifetime distribution curve of cells treated with 10 µL of Fe_3O_4@SiO_2@Zn[ATSM]/A with 1% DMSO in PC3 cells. λex = 810 nm, laser power was 3.6 mW.

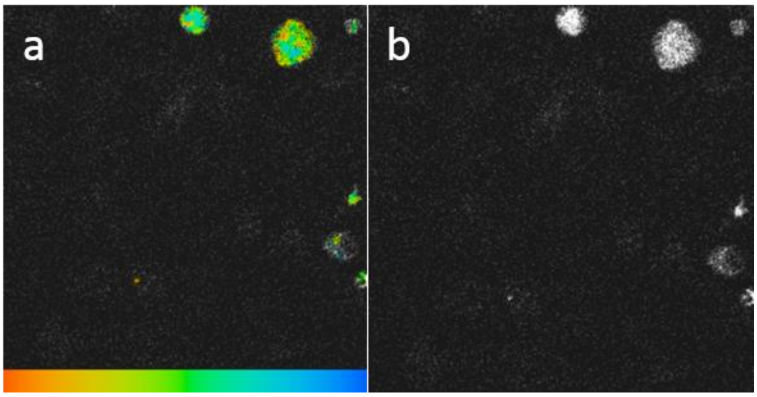

Figure S57. Fluorescence lifetime imaging microscopy (FLIM) micrographs of cells treated with 10 µL of Fe_3O_4@SiO_2@Zn[ATSM]/A with 1% DMSO, cells were incubated at 37 ºC for 15 min, where **a)** the fluorescence lifetime mapping and scale bar of Fe_3O_4@SiO_2@Zn[ATSM]/A in PC3 cells, and **b)** two-photon fluorescence intensity diagram of Fe_3O_4@SiO_2@Zn[ATSM]/A in PC3 cells.

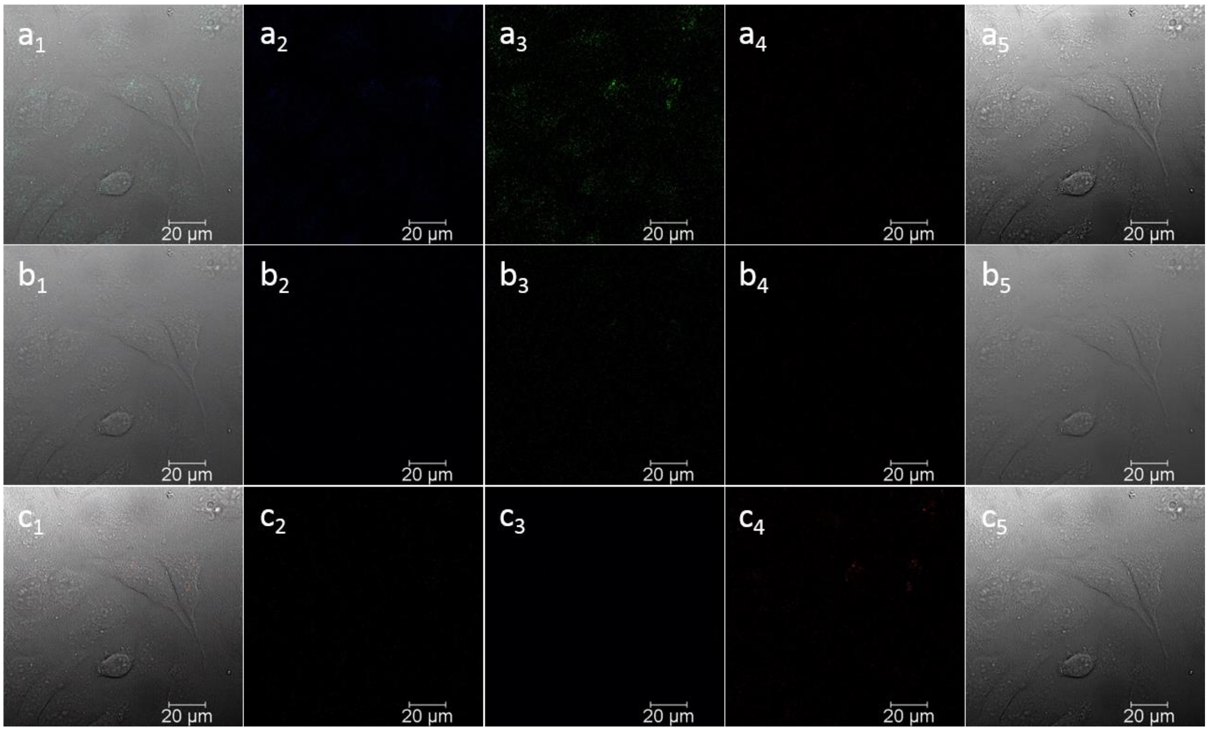

Figure S58. Confocal imaging of cancerous (PC3) cells incubated at 37 °C with 100 µl $Fe_3O_4@SiO_2@Zn[ATSM]/A$ in 1% DMSO (1:99% DMSO:RPMI) – 15 min incubation showing DIC-blue-green-red channel overlay (a_1,b_1 and c_1), blue channel (420 nm) (a_2, b_2 and c_2), green channel (515-530 nm) (a_3, b_3 and c_3) and red channel (605-675 nm) (a_4, b_4 and c_4) and DIC (a_5, b_5 and c_5). Scale bar: 20 µm.

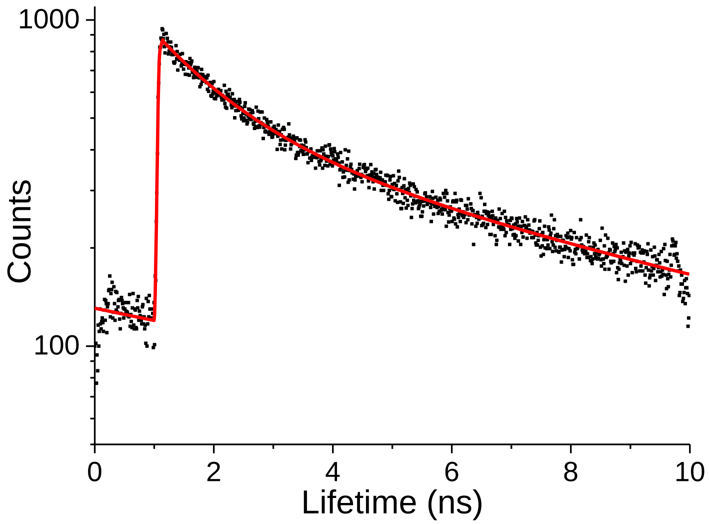

	χ^2	τ_1 (ps)	a_1 (%)	τ_2 (ps)	a_2 (%)	τ_m
CdSe	1.00	1243	54.1	6883.8	45.9	3831.1

Figure S59. Two-photon fluorescence lifetime decay traces and TCSPC data for CdSe quantum dots 1 mg/mL in $CHCl_3$. λ_{ex} = 810 nm; laser power: 1.3 mW.

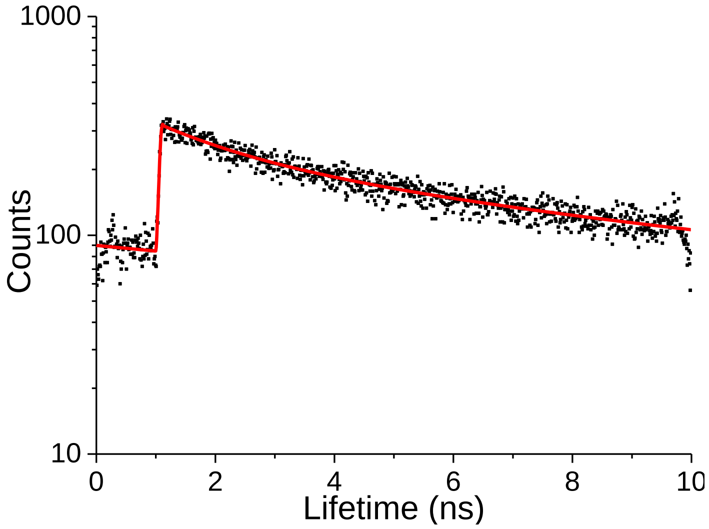

	χ^2	τ_1 (ps)	a_1 (%)	τ_2 (ps)	a_2 (%)	τ_m
$Cd_{(0.1)}Zn_{(0.9)}Se$	1.12	1471	40.3	8212.8	59.7	5497.8

Figure S60. Two-photon fluorescence lifetime decay traces and TCSPC data for CdSe quantum dots 1 mg/mL in $CHCl_3$. λ_{ex} = 810 nm; laser power: 1.2 mW.

11. Kinetic stability tests

Samples of each of the nanomaterials: $Fe_3O_4/Cd_{0.1}Zn_{0.9}Se@SiO_2@^{68}Ga$ (**G**), $Fe_3O_4@SiO_2@Zn[ATSM]/A@^{68}Ga$ (**H**) as emerging from radiochemistry experiments described above were incubated in PBS, and mouse plasma at 37 °C for 1 h, at a concentration of ca. 1 mg/mL. The mixtures were separated by using a 300 kDa MWCO filter and centrifuged at 14.000 r/min. The radioactivity left on the filters (NPs) was measured against the solvent filtrated and centrifugated (waste). Tables S10 and S11 summarise the results of these experiments. Although the samples corresponding to free ^{68}Ga(III) appeared to measure a radioactivity close to zero, it is worth noticing that the behaviour of ion-releasing nanocomposites in the aqueous media considered hereby does not necessarily reflect the *in vivo* release, and further studies are necessary to confirm that prior to animal experiments.

Table S10. Stability test of labelled $Fe_3O_4/Cd_{0.1}Zn_{0.9}Se@SiO_2$ with ^{68}Ga(III) (method 1)

Solutions	Incubation time	Total Activity (MBq)	Activity of wastes (MBq)	% of free ^{68}Ga(III)	Activity of the filter (MBq)	% of free labelled compound
**PBS**	1 h	0.074	0	0	0.074	>99.9 %
**Mouse plasma**	1 h	0.111	0	0	0.111	>99.9%

Table S11. Stability test of labelled $Fe_3O_4@SiO_2@Zn[ATSM]/A$ with ^{68}Ga(III) (method 3)

Solutions	Incubation time	Total Activity (MBq)	Activity of wastes (MBq)	% of free ^{68}Ga(III)	Activity of the filter (MBq)	% of free labelled compound
**PBS**	1 h	0.111	0	0	0.111	>99.9 %
**Mouse plasma**	1 h	0.148	0	0	0.148	>99.9%
